# Sunlight-powered kHz rotation of a hemithioindigo-based molecular motor

**DOI:** 10.1038/ncomms9406

**Published:** 2015-09-28

**Authors:** Manuel Guentner, Monika Schildhauer, Stefan Thumser, Peter Mayer, David Stephenson, Peter J. Mayer, Henry Dube

**Affiliations:** 1 Department of Chemistry, Ludwig-Maximilians-Universität München, Butenandtstrasse 5-13, 81377 München, Germany

## Abstract

Photodriven molecular motors are able to convert light energy into directional motion and hold great promise as miniaturized powering units for future nanomachines. In the current state of the art, considerable efforts have still to be made to increase the efficiency of energy transduction and devise systems that allow operation in ambient and non-damaging conditions with high rates of directional motions. The need for ultraviolet light to induce the motion of virtually all available light-driven motors especially hampers the broad applicability of these systems. We describe here a hemithioindigo-based molecular motor, which is powered exclusively by nondestructive visible light (up to 500 nm) and rotates completely directionally with kHz frequency at 20 °C. This is the fastest directional motion of a synthetic system driven by visible light to date permitting materials and biocompatible irradiation conditions to establish similarly high speeds as natural molecular motors.

## Abstract (web summary)

Although photodriven molecular motors—capable of converting light into unidirectional motion—hold promise for many applications, these typically require ultraviolet light. Here, the authors design and analyse a motor that can operate at high speeds (kHz) under visible light at ambient temperature.

## Graphical abstract

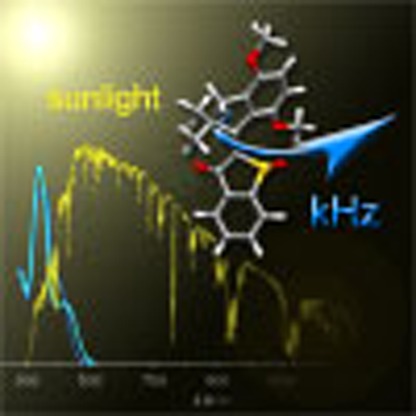

Synthetic photo-driven molecular motors^1^,^2^,^3^,^4^,^5^ allow harnessing light energy and converting it into directional motion against the equilibrating force of the ‘Brownian storm'^6^,^7^. They can be used to effectively drive a molecular system away from equilibrium and thus represent the essential component to power future nanomachinery^8^,^9^,^10^,^11^. Since Koumura *et al*.^1^ and Kelly *et al*.^12^ reported the first synthetic molecular motors in 1999, intriguing applications have been put forward^13^,^14^,^15^ demonstrating unique functions that cannot be established at the molecular scale in any other way^16^. However, to compete with the high efficiency and versatility of natural molecular motors^17^,^18^,^19^ considerable efforts have still to be made for synthetic systems^20^,^21^,^22^. Most light-powered molecular motors require damaging ultraviolet light to perform their task, which is a major drawback for biological or smart materials applications. At present, only a few motor systems are available that undergo unidirectional 360° rotation using visible light, but the speed of their motion is slow, which again impedes applications in (heat) sensitive environments^23^,^24^. In the following, we report on a novel molecular motor **1**, that performs a full (360°) rotation powered by visible light (up to 500 nm, that is, at the maximum wavelength of sunlight) with >95% unidirectionality and at a very fast rate (1 kHz at 20 °C). Thus, our new molecular motor system enables for the first time to power fast unidirectional rotation under ambient and non-damaging conditions, representing the next crucial step towards developing highly efficient nanomachines that work at materials and biocompatible conditions.

Our new molecular motor is based on the hemithioindigo (HTI) chromophore^25^, an emerging photoswitch^26^,^27^,^28^,^29^,^30^,^31^,^32^,^33^ that can be operated exclusively by visible light in both switching directions. HTI consists of a thioindigo and a stilbene fragment, which are connected via a central double bond. On irradiation, HTI undergoes efficient and reversible *Z*/*E* or *E*/*Z* photoisomerization^34^,^35^,^36^,^37^. To confine light-induced rotations around the central double bond to one direction and exclude unwanted back-movements, we implemented additional stereochemical elements to the HTI framework: the sulfur atom was oxidized to the corresponding sulfoxide, introducing a sulfur-based stereocentre (*R*- or *S*-configuration), and sterical crowding at the ring-fused stilbene fragment led to helical twisting (*P*- or *M*-helicity) around the central double bond. Combined with its *Z* and *E* isomeric forms, motor **1** can thus assume four different diastereomeric structures for each configuration of the stereogenic sulfur centre, for example, *Z*-(*S*)-(*P*), *Z*-(*S*)-(*M*), *E*-(*S*)-(*M*) and *E*-(*S*)-(*P*), which can be distinguished by conventional spectroscopic methods. This molecular set-up is related to the Feringa motor system consisting of a sterically overcrowded double bond in combination with carbon-based stereogenic centres^1^,^38^. In our case, considerably smaller sterical hindrance between the thioindigo and stilbene fragment is needed to warrant unidirectional rotation.

## Results

### Synthesis

**Figure 1:**
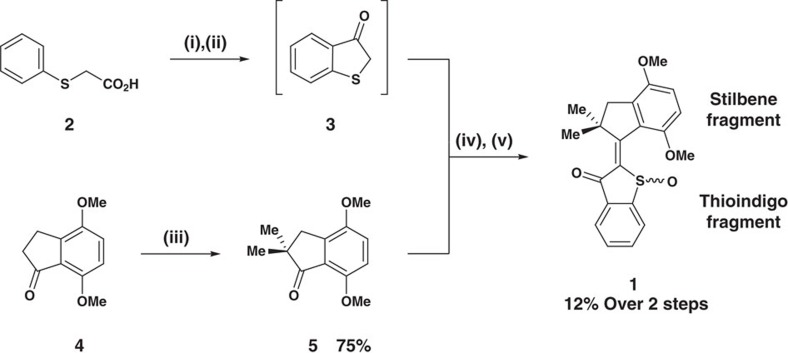
Synthesis of molecular motor 1. (i) SOCl_2_, DMF (cat.), 75 °C, 1 h. (ii) AlCl_3_, (CH_2_Cl)_2_, 0–23 °C, 1 h. (iii) NaH, (CH_2_OMe)_2_, 84 °C, 45 min, then MeI, 0–23 °C, 24 h. (iv) BF_3_·OEt_2_, CH_2_Cl_2_, 23 °C, 2 d. (v) H_2_O_2_, AcOH, 23 °C, 2 h.

**Figure 2:**
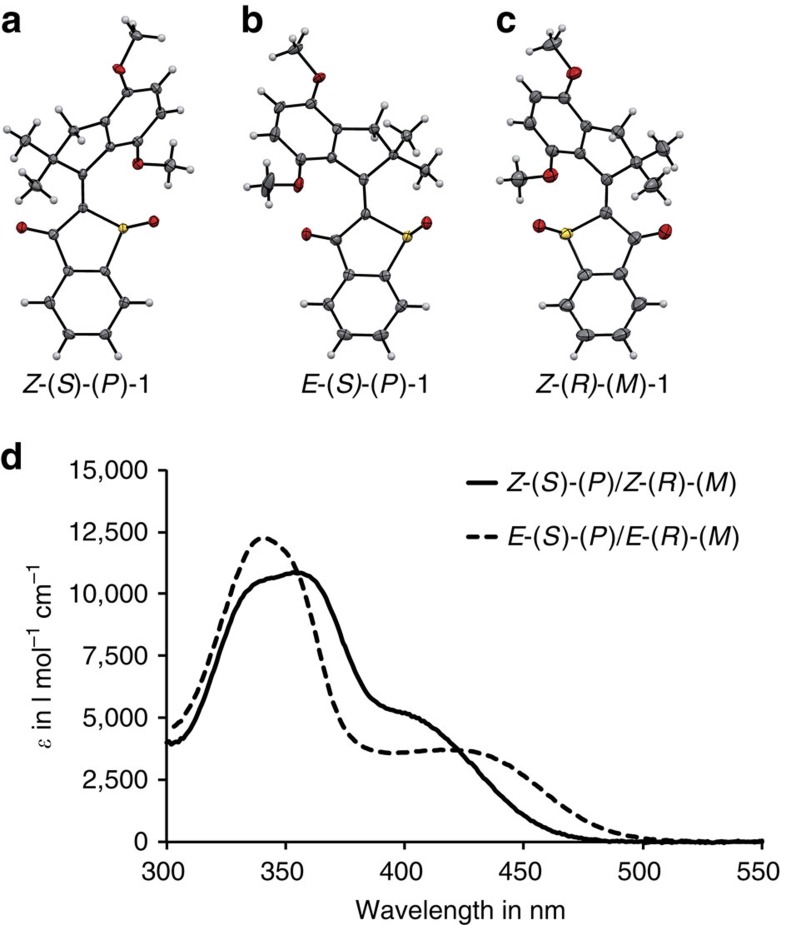
Molecular structure and absorption characteristics of motor 1. (**a**) Crystal structure of racemic *Z*-(*S*)-(*P*)/*Z*-(*R*)-(*M*) isomers, only the *Z*-(*S*)-(*P*) isomer is shown (CCDC 1061969). (**b**) Crystal structure of racemic *E*-(*S*)-(*P*)/*E*-(*R*)-(*M*) isomers, only the *E*-(*S*)-(*P*) isomer is shown (CCDC 1061970). (**c**) Crystal structure of enantiomerically pure *Z*-(*R*)-(*M*) isomer (CCDC 1406625). (**d**) Extinction coefficients of the *Z*-(*S*)-(*P*)/*Z*-(*R*)-(*M*) isomers (—) and the *E*-(*S*)-(*P*)/*E*-(*R*)-(*M*) isomers (–––) in CH_2_Cl_2_.

Motor **1** was synthesized in three steps starting from known, easily accessible precursors (Fig. 1). Phenylthioacetic acid **2** (ref. 39) was converted into the corresponding acid chloride, which subsequently underwent cyclization via intramolecular Friedel–Crafts acylation to give benzothiophenone **3**. Because of its instability and high tendency to dimerize, benzothiophenone **3** was used in the next synthetic step in crude form. Commercially available 4,7-dimethoxy-1-indanone (**4**) was methylated twice in the 2-position using sodium hydride and methyl iodide to give indanone **5**. Benzothiophenone **3** was subsequently condensed with indanone **5** using boron trifluoride diethyl etherate, after which a final oxidation with hydrogen peroxide furnished sulfoxide motor **1**. Crystals suitable for X-ray analysis were obtained for two isomeric forms of racemic motor **1**, the *Z*-(*S*)-(*P*)/*Z*-(*R*)-(*M*) (CCDC 1061969) and the *E*-(*S*)-(*P*)/*E*-(*R*)-(*M*) (CCDC 1061970) isomers, enantiomerically pure *Z*-(*R*)-(*M*) (CCDC 1406625) isomer (Fig. 2a–c), as well as for both *Z* and *E* isomers of the corresponding unoxidized HTI (CCDC 1408096 and CCDC 1408097, see Supplementary Figs 37 and 38 and Supplementary Tables 2 and 3).

### Conformational analysis

The (*S*) and (*R*) enantiomeric forms of motor **1** are stable (also under irradiation conditions as shown in Supplementary Fig. 14) and could easily be separated using chiral high-performance liquid chromatography (HPLC). For the following spectroscopic studies however, the racemic mixture was analysed for convenience reasons. The *Z*-(*S*)-(*P*)/*Z*-(*R*)-(*M*) isomers are the thermodynamically stable form of motor **1**, which can be obtained in 75% yield by heating a toluene-*d*_*8*_ solution of **1** to 100 °C for 8 h, as analysed by nuclear magnetic resonance (NMR) spectroscopy. Under these conditions also 25% of the *E*-(*S*)-(*P*)/*E*-(*R*)-(*M*) isomers are obtained. When a solution of **1** in xylene-*d*_*10*_ is heated to 130 °C for 12 h, 73% of the *Z*-(*S*)-(*P*)/*Z*-(*R*)-(*M*) isomers and 27% of the *E*-(*S*)-(*P*)/*E*-(*R*)-(*M*) isomers are obtained (Supplementary Fig. 22). This thermal behaviour translates into an energy difference of 0.81 kcal mol^−1^ between the *Z* and *E* diastereomeric pairs according to the relation of the change of Gibbs free energy and the equilibrium constant–Δ*G*=*RT*ln *K*. First-order kinetic analysis of thermal conversion from the *E*-(*S*)-(*P*)/*E*-(*R*)-(*M*) to the *Z*-(*S*)-(*P*)/*Z*-(*R*)-(*M*) isomers provided an energy barrier of 29.54 kcal mol^−1^ for the process (Supplementary Fig. 21). With such high thermal stability, that is, a half-life of 16 years for the metastable *E*-(*S*)-(*P*)/*E*-(*R*)-(*M*) isomers at 25 °C, the two *Z* and *E* diastereomers could conveniently be separated by conventional chromatography and studied individually. Direct assignment of the two separated species to the corresponding diastereomers was straightforward in solution, as NOESY NMR spectra showed specific cross-signals between the thioindigo and the stilbene part of the molecule (Supplementary Figs 6 and 11). In addition, crystal structure analysis unambiguously confirmed our assignment of the species and their solution spectra. We were able to grow crystals suitable for structure analysis from the second fraction obtained after chromatographic separation, which could be assigned to the *Z*-(*S*)-(*P*)/*Z*-(*R*)-(*M*) isomers (CCDC 1061969). The ^1^H NMR and ultraviolet/visible (ultraviolet/vis) spectra of the same crystal batch were recorded, showing only one single species to be present in solution. Thus, we could directly assign the corresponding NMR signals as well as the ultraviolet/vis spectrum to the *Z*-(*S*)-(*P*)/*Z*-(*R*)-(*M*) isomers (see also Supplementary Figs 1–6 and Supplementary Fig. 12, respectively).

In a similar way, the first fraction obtained after chromatographic separation and corresponding NMR and ultraviolet/vis solution spectra could be assigned to the *E*-(*S*)-(*P*)/*E*-(*R*)-(*M*) isomeric form (see Supplementary Figs 7–11 for details). The absorption spectral behaviour of motor **1** (Fig. 2b)—hypsochromic absorption of the *Z* (up to 470 nm) and bathochromic absorption of the *E* isomer (up to 505 nm)—is similar to typical HTI photoswitches^40^.

The crystal structures of the *Z*-(*S*)-(*P*) and *Z*-(*R*)-(*M*) isomers (CCDC 1061969) represent the global thermodynamic minimum of motor **1**; the structure of the *Z*-(*S*)-(*P*) isomer is shown in Fig. 2a. In this structure, substituents at the double bond are twisted out of planarity, leading to a helical arrangement, in which the methoxy-substituted aromatic ring of the fused stilbene fragment is oriented behind the sulfoxide oxygen atom. On the other side of the double bond, the two methyl substituents of the five-membered aliphatic ring are bisected by the carbonyl oxygen atom of the thioindigo fragment. The helical twist is clearly a result of steric repulsion between the substituents on the stilbene and the thioindigo fragment, which leads to a SC=CC(Ar) dihedral angle of 12.8°. This value is considerably larger than that observed in sterically unrestrained HTIs. The bond length of the central double bond is 1.358 Å and only slightly longer compared to planar HTIs^40^. The mirror image of that structure is reproduced in the crystal structure of the enantiomerically pure *Z*-(*R*)-(*M*) isomer (Fig. 2c, CCDC 1406625).

In solution, less structural information is available but several details could be inferred from NMR analysis. The NOESY NMR spectrum (Supplementary Fig. 6) allowed not only to confirm the *Z* configuration of the double bond in solution, but also to obtain relative distances between protons of the thioindigo fragment and the methyl and methoxy groups of the stilbene fragment. On the basis of relative signal intensities, the shortest H–H distance between the thioindigo and the stilbene fragment is found for one methyl group and the aromatic proton in *ortho*-position to the carbonyl group. On the other side of the double bond the methoxy group located behind the sulfoxide is slightly farther away, but an NOE coupling to the proton in *ortho*-position of the sulfur atom is still visible. The second methyl group shows no NOE couplings to protons of the thioindigo fragment. This relative order of distances is confirmed exactly by crystal structure analysis ,where the methyl group pointing towards the sulfur oxygen atom is closest to the thioindigo fragment, followed by the aforementioned methoxy group. The second methyl group is located at the third-closest distance from the thioindigo fragment. Both methoxy groups show additional strong NOE couplings to the neighbouring aromatic protons of the stilbene fragment, but no NOE couplings to the aliphatic CH_2_ signals are seen. Again this corresponds very well with the conformation seen in the crystal structure, where both methoxy groups point towards the aromatic protons of the stilbene fragment. A Karplus cross-peak analysis of the aliphatic part of the HMBC NMR spectrum (Supplementary Fig. 4) established that the conformation of the five-membered ring of the stilbene fragment in solution is also very similar to that observed in the crystalline state. Two HMBC signals are not observed, which is due to 90.0° dihedral angles between the corresponding proton and carbon atoms. Dihedral angles of 93.6° and 96.8° are indeed found in the crystal structure between the very same atoms. From this qualitative NMR analysis, we conclude that the conformation of *Z*-(*S*)-(*P*)/*Z*-(*R*)-(*M*)-**1** in solution is very similar to the one found in the crystal.

Among the metastable forms of motor **1**, the *E*-(*S*)-(*P*)/*E*-(*R*)-(*M*) isomers represent the most stable configuration. The crystal structure of the *E*-(*S*)-(*P*) isomer (CCDC 1061970) is shown in Fig. 2b. The geometry is again helical, with a dihedral angle C(carbonyl)C=CC(Ar) of 16.2°. The two methyl groups of the fused five-membered ring of the stilbene fragment are now bisected by the oxygen atom of the sulfoxide group. At the same time, the aryl ring of the stilbene fragment is oriented to the same side as the sulfoxide oxygen atom.

An NMR analysis similar to the one conducted for the *Z*-(*S*)-(*P*)/*Z*-(*R*)-(*M*) isomers (Supplementary Figs 7–11) showed again very close resemblance between the solution conformation of *E*-(*S*)-(*P*)/*E*-(*R*)-(*M*)-**1** and the one found in the crystal.

### Behaviour under irradiation conditions

**Figure 3:**
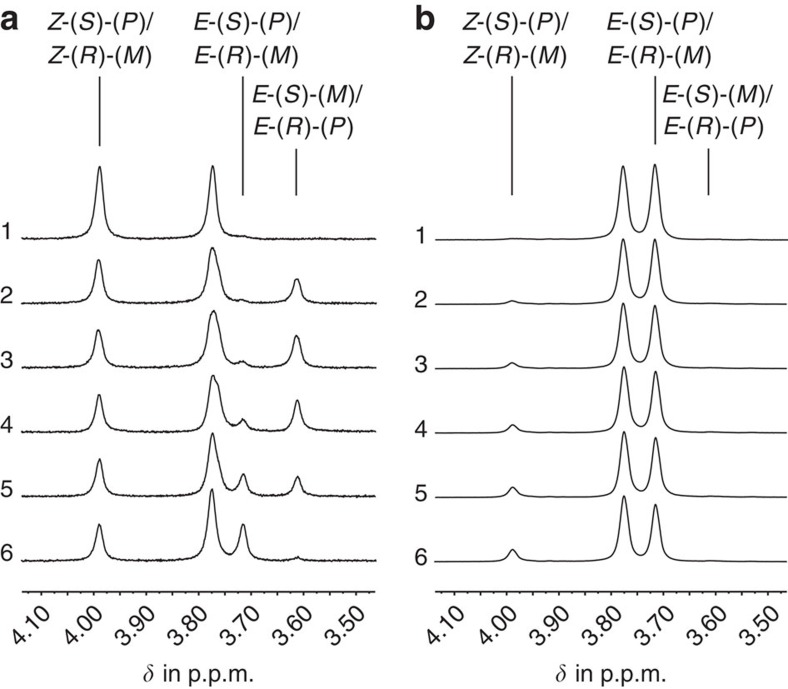
^1^H NMR spectra of motor 1 recorded at –90 °C (CD_2_Cl_2_, 400 MHz). Signals of the methoxy groups are shown. All spectra are normalized to the intensity of the residual solvent signal. (**a**) Spectra acquired during 460 nm irradiation of the *Z*-(*S*)-(*P*)/*Z*-(*R*)-(*M*) isomers of motor **1** (spectra 1–3) and during thermal isomerization of the intermediate *E*-(*S*)-(*M*)/*E*-(*R*)-(*P*) isomers (spectra 4–6). 1: ^1^H NMR spectrum of *Z*-(*S*)-(*P*)/*Z*-(*R*)-(*M*) isomers before irradiation. 2: After 2 min of 460 nm irradiation. 3: After 4 min of 460 nm irradiation the PSS is reached. 4: After reaching the PSS the light is turned off for 2 min. 5: Light turned off after reaching the PSS for 40 min. 6: Light turned off after reaching the PSS for 60 min. (**b**) Spectra acquired during 460 nm irradiation of the *E*-(*S*)-(*P*)/*E*-(*R*)-(*M*) isomers of motor **1** in 2 min time intervals. Only the *Z*-(*S*)-(*P*)/*Z*-(*R*)-(*M*) isomers are seen but no *E*-(*S*)-(*M*)/*E*-(*R*)-(*P*) isomers.

When irradiating a solution of **1** in CD_2_Cl_2_ with 405 nm light at 23 °C, a photostationary state (PSS) consisting of 22% *Z*-(*S*)-(*P*)/*Z*-(*R*)-(*M*) and 78% *E*-(*S*)-(*P*)/*E*-(*R*)-(*M*) isomers is formed, as judged by integration of indicative signals in the ^1^H NMR spectrum. Irradiation with 490 nm light leads to a PSS of 83% *Z*-(*S*)-(*P*)/*Z*-(*R*)-(*M*) and 17% *E*-(*S*)-(*P*)/*E*-(*R*)-(*M*) isomers. The quantum yields for photoisomerization at ambient temperature are 30% for the *Z* to *E* isomerization and 12% for the *E* to *Z* isomerization. Thus, at 23 °C only two different diastereomeric forms are observed under irradiation conditions (Supplementary Fig. 23). However, irradiating the solution at −90 °C allowed us to follow the isomerization process of the double bond in greater detail as shown in Fig. 3. On cooling the ^1^H NMR spectrum of *Z*-(*S*)-(*P*)/*Z*-(*R*)-(*M*)-**1** did not change significantly, which preserved signal identity (Supplementary Fig. 26). When a solution of pure *Z*-(*S*)-(*P*)/*Z*-(*R*)-(*M*)-**1** in CD_2_Cl_2_ was irradiated with 460 nm light at –90 °C, a new set of signals was observed in up to 45% signal intensity. (Fig. 3a, see also Supplementary Figs 27 and 28 for the full spectra). The chemical shifts of the new signals are more similar to those of the *E*-(*S*)-(*P*)/*E*-(*R*)-(*M*) isomers than to those of the *Z*-(*S*)-(*P*)/*Z*-(*R*)-(*M*) isomers. Switching off irradiation leads to the decrease of the new set of signals and at the same time to a similar increase of only the *E*-(*S*)-(*P*)/*E*-(*R*)-(*M*) isomer signals. After 1 h, the new set of signals had completely disappeared. An additional annealing experiment also demonstrated the complete thermal conversion of these signals to the *E*-(*S*)-(*P*)/*E*-(*R*)-(*M*) isomer signals (Supplementary Fig. 31). With these experiments, we were able to unambiguously prove that photoirradiation of *Z*-(*S*)-(*P*)/*Z*-(*R*)-(*M*)-**1** leads exclusively to the population of an intermediate *E* isomeric state before a thermal step converts it completely (>95%) into the *E*-(*S*)-(*P*)/*E*-(*R*)-(*M*) isomers. We therefore assign the new set of signals to the *E*-(*S*)-(*M*)/*E*-(*R*)-(*P*) isomeric form. An energy barrier ΔG* of 13.1 kcal mol^−1^ was found for the thermal conversion between the two *E* isomers by first-order kinetic analysis (Supplementary Fig. 30). To test if the intermediate *E*-(*S*)-(*M*)/*E*-(*R*)-(*P*) isomeric state is populated at higher temperatures, a CD_2_Cl_2_ solution of *E*-(*S*)-(*P*)/*E*-(*R*)-(*M*)-**1** at 22 °C was shock-frozen in liquid N_2_ and allowed to warm to –90 °C to record a ^1^H NMR spectrum at this temperature immediately after thawing. No signals of the *E*-(*S*)-(*M*)/*E*-(*R*)-(*P*) isomers were observed in this experiment, showing that also at 22 °C the *E*-(*S*)-(*P*)/*E*-(*R*)-(*M*) isomers are present in >95% (Supplementary Fig. 32). This gives an energy difference between the two *E* isomers of at least 1.73 kcal mol^−1^ according to the relation of the change of Gibbs free energy and the equilibrium constant –Δ*G*=*RT*ln *K*.

Complementary experiments were performed with a CD_2_Cl_2_ solution of pure *E*-(*S*)-(*P*)/*E*-(*R*)-(*M*)-**1** at –90 °C (Supplementary Fig. 33), using again 460 nm light for the irradiation (Fig. 3b, see also Supplementary Fig. 34 for the full spectra). In this case no new signals were observed, but the signals of *Z*-(*S*)-(*P*)/*Z*-(*R*)-(*M*)-**1** were found to build up immediately after irradiation. At higher power of irradiation also the *E*-(*S*)-(*M*)/*E*-(*R*)-(*P*) isomers were observed, but only as a photoproduct of the *Z*-(*S*)-(*P*)/*Z*-(*R*)-(*M*) isomers after the latter were already present (Supplementary Fig. 35b). An irradiation experiment in diethyl ether-*d*_*10*_ at –100 °C also did not furnish any new signals of a fourth isomeric form (Supplementary Fig. 36). Thus, the intermediate *Z*-(*S*)-(*M*)/*Z*-(*R*)-(*P*) isomers were not found at the NMR-accessible temperature range. The barrier for the thermal conversion of *Z*-(*S*)-(*M*)/*Z*-(*R*)-(*P*)-**1** to *Z*-(*S*)-(*P*)/*Z*-(*R*)-(*M*)-**1** must therefore be <12.0 kcal mol^−1^.

Although we could not directly observe the *Z*-(*S*)-(*M*)/*Z*-(*R*)-(*P*) intermediate, our experiments established that under the same irradiation conditions (460 nm) the *Z*-(*S*)-(*P*)/*Z*-(*R*)-(*M*) and *E*-(*S*)-(*P*)/*E*-(*R*)-(*M*) isomers are interconverted via two different pathways. *Z*-(*S*)-(*P*)/*Z*-(*R*)-(*M*)-**1** is first converted photochemically into the *E*-(*S*)-(*M*)/*E*-(*R*)-(*P*) isomers. From this intermediate state a thermal step leads to complete formation of *E*-(*S*)-(*P*)/*E*-(*R*)-(*M*)-**1**, which breaks microscopic reversibility. In contrast, photoirradiation of *E*-(*S*)-(*P*)/*E*-(*R*)-(*M*)-**1** leads to *Z*-(*S*)-(*P*)/*Z*-(*R*)-(*M*)-**1**, omitting the *E*-(*S*)-(*M*)/*E*-(*R*)-(*P*) isomeric state, which otherwise would have been observable in our experiments. Since each conversion constitutes a 180° rotation around the central double bond, the observed two different pathways are only consistent with a unidirectional 360° rotation. As the thermal step converting the *E* isomeric forms proceeds to >95% and photoirradiation of the *E*-(*S*)-(*P*)/*E*-(*R*)-(*M*) isomers did not populate any of the intermediate *E*-(*S*)-(*M*)/*E*-(*R*)-(*P*) state, we conclude that **1** rotates with >95% in one direction. This finding gives indirect evidence that an intermediate *Z*-(*S*)-(*M*)/*Z*-(*R*)-(*P*) isomeric state must exist, which is at least 1.1 kcal mol^−1^ higher in energy than the *Z*-(*S*)-(*P*)/*Z*-(*R*)-(*M*) state.

As the photoisomerization steps are very fast and cannot be followed by NMR spectroscopy, the rate-determining step of rotation for motor **1** is the thermal *E*-(*S*)-(*M*)/*E*-(*R*)-(*P*) to *E*-(*S*)-(*P*)/*E*-(*R*)-(*M*) conversion. With the established energy barrier of 13.1 kcal mol^−1^, a maximum rotation frequency at 20 °C of 1 kHz can be realized, which is the fastest rate for a molecular motor powered by visible light to date. Photoisomerization of the *Z*-(*S*)-(*P*)/*Z*-(*R*)-(*M*) isomers with a 505 nm diode also resulted in the formation of *E*-(*S*)-(*P*)/*E*-(*R*)-(*M*) isomers (Supplementary Fig. 24), showing that motor **1** can be operated at a wide range of wavelengths in the visible part of the spectrum. Separate irradiation of both pure *Z*-(*S*)-(*P*)/*Z*-(*R*)-(*M*) and *E*-(*S*)-(*P*)/*E*-(*R*)-(*M*) isomers with direct sunlight for 20 min resulted in a photostationary state composed of 47% of the former and 53% of the latter isomers showing that motor **1** can efficiently be powered using sunlight (Supplementary Fig. 25).

### Theoretical description

**Figure 4:**
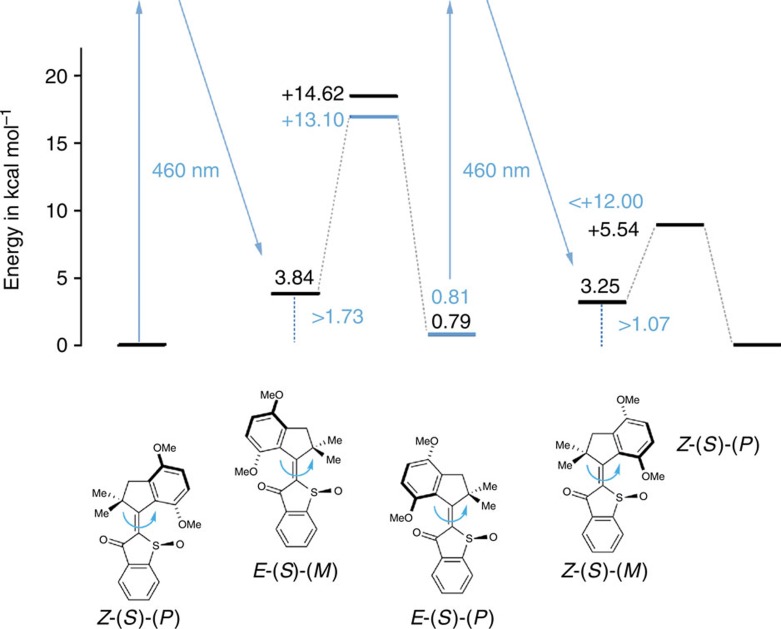
Energy profile describing visible light-powered unidirectional rotation of motor 1. Only the S-enantiomers are shown for clarity. Energies labelled in black are derived from the theoretical description of motor **1** at the DFT MPW1K level of theory using the 6–31+G(d,p) basis set. Energies labelled in blue were obtained experimentally. In the first step, the thermodynamically stable *Z*-(*S*)-(*P*) isomer undergoes clockwise (seen from the thioindigo side) rotation to the metastable *E*-(*S*)-(*M*) isomer on irradiation with visible light (400–505 nm). A thermal clockwise (seen from the thioindigo side) rotation fully converts the *E*-(*S*)-(*M*) isomer to the more stable *E*-(*S*)-(*P*) isomer. Another irradiation step leads again to clockwise rotation and the metastable *Z*-(*S*)-(*M*) isomer is formed. A final thermal clockwise rotation regenerates the *Z*-(*S*)-(*P*) isomeric state.

To gain deeper insight into the function of our new motor system, we performed a theoretical analysis of the ground-state energy profile at the DFT MPW1K level of theory using the 6–31+G(d,p) basis set (Supplementary Fig. 39). The structures of *Z*-(*S*)-(*P*) and *E*-(*S*)-(*P*) isomers were optimized starting from the crystal analytical data of the *Z*-(*S*)-(*P*) isomer. The optimized geometries and the geometries obtained experimentally from crystal structure analysis agree very well (see Supplementary Table 4 for selected bond lengths and angles). The structures of the *Z*-(*S*)-(*M*) and *E*-(*S*)-(*M*) isomers were optimized starting from the helical inverted structures of *Z*-(*S*)-(*P*) and *E*-(*S*)-(*P*) isomers, which were then allowed to fully relax. Transition states were then obtained starting from the partially restricted *Z*-(*S*)-(*M*) and *E*-(*S*)-(*M*) minimum structures, which were optimized fully relaxed at a second stage of the calculations (Supplementary Figs 40 and 41). All optimized structures were confirmed to be stationary points by frequency analysis. No imaginary modes were found for the minima structures and only one imaginary mode was present for the transition states, proving the latter to be first order saddle points on the hyper-potential energy surface. The calculated ground-state energy profile is shown in Fig. 4, together with the experimentally obtained data. The theoretically found energies are qualitatively in complete agreement with our experimental findings. Moreover, all experimentally quantified energies match those obtained theoretically exceptionally well^41^. The *Z*-(*S*)-(*P*) isomer is found indeed as a global minimum structure in the theoretical description, while the *E*-(*S*)-(*P*) isomer is only 0.8 kcal mol^−1^ higher in energy. This finding is perfectly confirmed experimentally by the 75:25 and 73:27 ratio of *Z*-(*S*)-(*P*) and *E*-(*S*)-(*P*) isomers observed in solution after heating to 100 °C and 130 °C, respectively (corresponding to an energy difference of 0.81 kcal mol^−1^). The *E*-(*S*)-(*M*) isomer is 3.03 kcal mol^−1^ higher in energy compared with the *E*-(*S*)-(*P*) isomer in the theoretical description, which explains why the *E*-(*S*)-(*M*) isomer is completely converted (that is, >95%, given the accuracy of NMR spectroscopy) to the *E*-(*S*)-(*P*) isomer in the thermal step. For this conversion, the theoretical description gives a barrier of 14.62 kcal mol^−1^, a value that again agrees remarkably well with the experimentally measured 13.10 kcal mol^−1^. Interestingly, the calculated energy barrier of the corresponding thermal conversion of *Z*-(*S*)-(*M*) to *Z*-(*S*)-(*P*) is considerably smaller with only 5.54 kcal mol^−1^. Thus, a half-life of 0.75 μs would be found for this process at –90 °C—a time range that is not accessible with conventional low-temperature NMR or ultraviolet/vis spectroscopic techniques. The theoretically found energy difference between the *Z*-(*S*)-(*M*) and *Z*-(*S*)-(*P*) isomers is 3.26 kcal mol^−1^, which is sufficiently high to convert the *Z*-(*S*)-(*M*) isomer completely in the thermal step. Again, this is in good agreement with our experiments, as we observe only *Z*-(*S*)-(*P*)/*Z*-(*R*)-(*M*) to *E*-(*S*)-(*M*)/*E*-(*R*)-(*P*) and not *Z*-(*S*)-(*P*)/*Z*-(*R*)-(*M*) to *E*-(*S*)-(*P*)/*E*-(*R*)-(*M*) photoisomerization when irradiating the *Z*-(*S*)-(*P*)/*Z*-(*R*)-(*M*) isomers of motor **1**.

On the basis of the theoretical minimum structures, we also calculated the corresponding ^1^H NMR chemical shifts at the DFT MPW1K level of theory using the 6–31+G(d,p) basis set. A detailed comparison of the chemical shifts obtained experimentally at –90 °C in CD_2_Cl_2_ solution and the theoretical values is given in Supplementary Table 1. Again, the theoretical results compare remarkably well with the experimentally obtained values (Supplementary Fig. 29). The observed change of signals during operation of motor **1** are perfectly reproduced in the theoretical description, which further supports our assignment of the thermally unstable intermediate to the *E*-(*S*)-(*M*)/*E*-(*R*)-(*P*) isomers of motor **1**. In addition, the extinction coefficients and circular dichroism spectra of all four diastereomers were calculated using the same level of theory but an increased 6–311++G(d,p) basis set (Supplementary Figs 13 and 20).

### Separation of enantiomers

To harness unidirectional rotation of motor **1** in applications, the enantiomers have to be separated, which was conveniently achieved using chiral HPLC. The separation of all four diastereomeric forms, *Z*-(*S*)-(*P*), *Z*-(*R*)-(*M*), *E*-(*S*)-(*P*) and *E*-(*R*)-(*M*), was possible in one single run using a CHIRALPAK IC column from Diacel. Circular dichroism spectra of these four isomers were measured (Supplementary Figs 15–19) and could be assigned to the corresponding absolute configuration of the sulfoxide stereocenter, by comparison with the theoretically obtained circular dichroism spectra calculated at the DFT MPW1K level of theory using the 6–311++G(d,p) basis set (Supplementary Fig. 20). These assignments were experimentally proven by crystal structure analysis of enantiomerically pure *Z*-(*R*)-(*M*) isomer (CCDC 1406625, Fig. 2c) obtained after semipreparative chiral HPLC separation (Supplementary Figs 15 and 37).

## Discussion

We have developed a novel HTI-based molecular motor **1** that is fuelled by visible light at wavelengths up to 500 nm. To unambiguously prove unidirectionality of the full 360° double-bond rotation, we have established the exact order by which different diastereomeric forms of **1** are interconverted under irradiation conditions. The maximum speed of rotation was obtained from kinetic analysis of the slowest interconversion step, which is the thermal helix inversion of the *E* isomer. According to our analysis, motor **1** is capable of kHz rotation at ambient temperatures (that is, 20 °C) with >95% unidirectionality, which sets a new standard to the performance and sustainability of synthetic molecular motors. With these exceptional properties, motor **1** can be used as a highly efficient molecular power unit for applications in sensitive environments that do not tolerate ultraviolet light or high temperatures. We believe that especially in the fields of material sciences and biology, our new motor system will be of great advantage and we will direct our future efforts towards the implementation of motor **1** into more complex nanomachinery and biological systems.

## Methods

### General

Reagents and solvents were obtained from Acros, Aldrich, Fluka, Merck or Sigma-Aldrich in the qualities puriss., p.a. or purum and used as received unless stated otherwise. Technical solvents were distilled before use for column chromatography and extraction on a rotary evaporator (Hei-VAP Value). Thin-layer chromatography (TLC) was conducted on Merck Silica 60 F254 TLC plates and visualization conducted with a ultraviolet lamp (254 or 366 nm). Deuterated solvents were obtained from Cambridge Isotope Laboratories and were used without further purification. Thiophenol and 4,7-dimethoxy-1-indanone were purchased at reagent grade from Sigma-Aldrich and were used as received. Boron trifluoride diethyl etherate was purchased from ABCR and freshly distilled before use. Column chromatography was performed with SiO_2_ 60 (Merck, particle size 0.063–0.200 mm) and distilled technical solvents. HPLC was performed on a Shimadzu HPLC system consisting of an LC-20AP solvent delivery module, a CTO-20A column oven, a SPD-M20A photodiode array ultraviolet/vis detector and a CBM-20A system controller using a semipreparative CHIRALPAK IC column (particle size 5 μm) from Diacel and HPLC grade solvents (2-PrOH and *n*-heptane) from Sigma-Aldrich and ROTH. ^1^H NMR and ^13^C NMR spectra were measured on a JEOL ECX 400 (400 MHz), Varian VNMRS 400 (400 MHz), Varian VNMRS 600 (600 MHz) or Bruker AVANCE III HD 800 (800 MHz) NMR spectrometer. Chemical shifts (*δ*) are reported in p.p.m. relative to the signal of tetramethylsilane. Residual solvent signals in the ^1^H and ^13^C NMR spectra were used as internal reference (CDCl_3_: *δ*_H_=7.260 p.p.m., *δ*_C_=77.16 p.p.m.; CD_2_Cl_2_: *δ*_H_=5.320 p.p.m., *δ*_C_=54.00 p.p.m.; toluene-*d*_*8*_: *δ*_H_=2.080 p.p.m., *δ*_C_=20.43 p.p.m.; *o*-xylene-*d*_10_: *δ*_H_=2.083 p.p.m., diethyl ether-*d*_10_: *δ*_H_=1.110 p.p.m.). The resonance multiplicity is indicated as s (singlet), d (doublet) or t (triplet). The coupling constant values (*J*) are given in hertz (Hz). Electron impact mass spectra were measured on a Finnigan MAT95 mass spectrometer. The most important signals are reported in *m/z* units with M as the molecular ion. Elemental analyses were performed in the micro analytical laboratory of the LMU Department of Chemistry on an Elementar Vario EL apparatus. Infrared spectra were recorded on a Perkin Elmer Spectrum BX-FT-IR instrument equipped with a Smith DuraSamplIR II ATR-device. Transmittance values are qualitatively described by wavenumber (cm^–1^) as very strong (*vs*), strong (*s*), medium (*m*), weak (*w*) and very weak (*vw*). Ultraviolet/vis spectra were measured on a Varian Cary 50 spectrophotometer. The spectra were recorded in CH_2_Cl_2_ in a quartz cuvette (1 cm). Absorption wavelengths (*λ*) are reported in nm and the extinction coefficients (*ɛ*) in l mol^–1 ^cm^–1^. Melting points (m.p.) were measured on a Büchi B-540 melting point apparatus in open capillaries.

### Photoisomerization experiments

Continuous irradiation of the solutions was conducted in NMR tubes in different deuterated solvents (CD_2_Cl_2_, diethyl ether-d10, toluene-*d*_*8*_ or *o*-xylene-*d*_*10*_). Irradiations at 23 °C were conducted using light-emitting diodes (LEDs) from Roithner Lasertechnik GmbH (405, 420, 470, 490, 505 and 515 nm). For low-temperature studies a Prizmatix Mic-LED-365 high-power collimated LED (365 nm), a Prizmatix Mic-LED-415 high-power collimated LED (415 nm) or a Prizmatix UHP-Mic-LED-460 ultra high-power collimated LED (460 nm) was used as light source, and the light beam was guided by a fibre-optic cable (0.39 NA, one SMA, one blank end) and pointed directly into the NMR tube during NMR measurements. The quantum yields of *Z*-(*S*)-(*P*)/*Z*-(*R*)-(*M*) to *E*-(*S*)-(*P*)/*E*-(*R*)-(*M*) photoisomerization and vice versa were measured at 22 °C in CH_2_Cl_2_. A solution of the respective isomer was irradiated at 420 nm for a certain time (typically 20 s) and the absorption changes were recorded. Using the Beer–Lambert law at suitable wavelengths the molar changes could be calculated. The amounts of absorbed photons were determined using 2-(4-methoxybenzylidene)-1-benzothiophen-3(*2H*)-one as calibration standard with known photoisomerization quantum yield^40^. A solution of this standard with the same optical density at 420 nm as the solutions of *Z*-(*S*)-(*P*)/*Z*-(*R*)-(*M*) or *E*-(*S*)-(*P*)/*E*-(*R*)-(*M*) isomers was used in the measurements to ensure equal amounts of absorbed photons in each case.

### Building block syntheses

2-Phenylthioacetic acid (**2**) was prepared according to a literature procedure^39^ from a commercially available thiophenol. 4,7-Dimethoxy-2,2-dimethyl-1-indanone (**5**) was prepared from a commercially available 4,7-dimethoxy-1-indanone based on an altered literature procedure^42^.

### 1-Benzothiophen-3(2*H*)-one (**3**)

To 2-phenylthioacetic acid (1.00 g, 5.90 mmol), thionylchloride (1.30 ml, 17.80 mmol) and dimethyl formamide (3 drops) were added, and the solution was stirred under reflux for 1 h. After evaporation of residual thionylchloride at 50 °C, the remaining 2-phenylthioacetic acid chloride was dissolved in 1,2-dichloroethane (5 ml) and subsequently cooled to 0 °C. Aluminium chloride (4.00 g, 29.70 mmol) was added slowly over a period of 2 min, followed by stirring for 5 min at 0 °C. The reaction mixture was allowed to warm to 23 °C and, after stirring for 1 h at this temperature, was poured on ice/water (150 ml). The aqueous phase was extracted with CH_2_Cl_2_ (3 × 150 ml), the combined organic phases were dried over sodium sulfate and the solvent was removed *in vacuo*. For the following synthetic step, crude benzothiophenone **3** was used without further purification.

### 4,7-Dimethoxy-2,2-dimethyl-1-indanone (**5**)

A solution of 4,7-dimethoxy-2,3-dihydro-*1H*-inden-1-one (1.00 g, 5.20 mmol) in glyme (5 ml) was added under argon atmosphere to a solution of sodium hydride (0.80 g, 60% in mineral oil, 20.80 mmol) in glyme (2 ml). After stirring the resulting solution under reflux for 45 min, the mixture was cooled to 0 °C and methyl iodide (1.30 ml, 2.90 g, 20.80 mmol) was added dropwise followed by stirring for 24 h at 23 °C. After addition of a saturated aqueous solution of ammonium chloride (100 ml), the mixture was extracted with ethyl acetate (3 × 150 ml). The combined organic phases were dried over sodium sulfate and the volatiles were removed *in vacuo*. The crude product was purified by flash column chromatography (SiO_2_, *i*Hex:EtOAc, 8:2 v/v) to afford indanone **5** (859 mg, 3.90 mmol, 75%) as a colourless liquid. TLC (SiO_2_, *i*Hex:EtOAc, 8:2 v/v): *R*_f_=0.15; ^1^H-NMR (600 MHz, CDCl_3_): *δ* 6.98 (d, *J*(H,H)=8.7 Hz, 1H, H-C(2)), 6.73 (d, *J*(H,H)=8.7 Hz 1H, H-C(1)), 3.89 (s, 3H, H-C(16)), 3.84 (s, 3H, H-C(15)), 2.85 (s, 2H, H-C(7)), 1.21 (s, 6H, H-C(13)), H-C(14)); ^13^C-NMR (150 MHz, CDCl_3_): *δ* 209.6 (C(9)), 152.4 (C(6)), 150.7 (C(3)), 143.1 (C(4)), 124.7 (C(5)), 116.9 (C(2)), 109.7 (C(1)), 56.2 (C(15), C(16)), 45.8 (C(8)), 39.5 (C(7)), 25.8 (C(13), C(14)); high-resolution electron ionization-MS (HREI-MS) (*m/z*): [M]^+^ calcd. for C_13_H_16_O, 220.1099; found, 220.1089.

### 2-(4,7-Dimethoxy-2,2-dimethyl-2,3-dihydro-1*H*-inden-1-ylidene)benzo[*b*]thiophen-3(2*H*)-one 1-oxide (**1**)

To a solution of **3** (278 mg, 1.90 mmol) and **5** (380 mg, 1.70 mmol) in CH_2_Cl_2_ (2.50 ml) boron trifluoride diethyl etherate (1.10 ml, 1.30 g, 9.20 mmol) was added and stirred for 24 h at 23 °C. Boron trifluoride diethyl etherate (1.00 ml, 1.20 g, 8.10 mmol) was added again and the resulting solution was stirred for another 24 h at 23 °C. The reaction mixture was then quenched with water (50 ml), extracted with ethyl acetate (3 × 50 ml) and dried over sodium sulfate. The solvent was removed *in vacuo* and the crude product was dissolved in a mixture of acetic acid (14 ml) and hydrogen peroxide (5.60 ml, 33% in H_2_O). After 2 h of stirring at 23 °C, the reaction mixture was neutralized with a saturated aqueous solution of sodium bicarbonate (150 ml). After extraction with ethyl acetate (3 × 150 ml), the combined organic layers were dried over sodium sulfate and the solvent was removed *in vacuo*. The crude mixture was purified by flash column chromatography (SiO_2_, *i*Hex:EtOAc, 6:4 v/v) to afford motor **1** (75 mg, 0.20 mmol, 12% based on **5**) as a yellow solid.

*Z*-(*S*)-(*P*)/*Z*-(*R*)-(*M*) isomers: m.p: 215 °C; TLC (SiO_2_, *i*Hex:EtOAc, 6:4 v/v): *R*_f_=0.08; ^1^H NMR (800 MHz; CD_2_Cl_2_): *δ* 7.99 (dd, *J*(H,H)=7.7 Hz, 1H, H-C(6)), 7.95 (dd, *J*(H,H)=7.7 Hz, 1H, H-C(3)), 7.83 (td, *J*(H,H)=7.5, 1.2 Hz, 1H, H-C(5)), 7.69 (td, *J*(H,H)=7.4, 1.0 Hz, 1H, H-C(4)), 7.05 (d, *J*(H,H)=8.8 Hz, 1H, H-C(14)), 6.85 (d, *J*(H,H)=8.8 Hz, 1H, H-C(13)), 4.03 (s, 3H, H-C(12)), 3.83 (s, 3H, H-C(16)), 2.99 (d, *J*(H,H)=16.4 Hz, 1H, H-C(18)), 2.91 (d, *J*(H,H)=16.4 Hz, 1H, H-C(18′)), 1.54 (s, 3H, H-C(20)), 1.52 (s, 3H, H-C(21)); ^13^C-NMR (201 MHz, CD_2_Cl_2_): *δ* 185.4 (C(1)), 170.7 (C(9)), 152.0 (C(11)), 151.1 (C(7)), 150.6 (C(15)), 144.0 (C(8)), 139.3 (C(17)), 136.3 (C(2)), 135.6 (C(5)), 132.2 (C(4)), 127.0 (C(10)), 126.8 (C(6)), 125.0 (C(3)), 116.9 (C(14)), 110.0 (C(13)), 56.5 (C(16)), 55.5 (C(12)), 52.4 (C(19)), 47.6 (C(18)), 28.6 (C(20)), 25.7 (C(21)); IR (): 3425*w*, 2940*w*, 2663*w*, 2476*vw*, 2004*vw*, 1673*s*, 1586*m*, 1541*s*, 1488*s*, 1451*s*, 1381*w*, 1360*w*, 1334*m*, 1319*w*, 1292*m*, 1194*m*, 1171*m*, 1113*m*, 1099*m*, 1063*vs*, 1035*vs*, 1004*m*, 991*m*, 955*s*, 938*m*, 916*m*, 879*m*, 821*m*, 806*s*, 790*m*, 759*s*, 748*s*, 713*s*, 696*m*, 669*m*, 689*m*, 660*s *cm^–1^; ultraviolet/vis (CH_2_Cl_2_): *λ*_max_ (*ɛ*) 400 (5,200 l mol^–1 ^cm^–1^), 355 (10,800 l mol^–1 ^cm^–1^), 341 (10,500 l mol^–1 ^cm^–1^) nm; HREI-MS (*m/z*): [M]^+^ calcd. C_21_H_20_O_4_S, 368.1082; found, 368.1070; analysis (calcd., found for C_21_H_20_O_4_S): C (68.46, 68.31), H (5.47, 5.47), S (8.70, 9.02).

*E*-(*S*)-(*P*)/*E*-(*R*)-(*M*) isomers: m.p: 186 °C; TLC (SiO_2_, *i*Hex:EtOAc, 6:4 v/v): *R*_f_=0.26; ^1^H NMR (800 MHz; CD_2_Cl_2_): *δ* 8.06 (d, *J*(H,H)=7.7 Hz, 1H, H-C(6)), 7.98 (d, *J*(H,H)=7.5 Hz, 1H, H-C(3)), 7.85 (td, *J*(H,H)=7.5, 1.3 Hz, 1H, H-C(5)), 7.73 (td, *J*(H,H)=7.4, 1.0 Hz, 1H, H-C(4)), 6.99 (d, *J*(H,H)=8.9 Hz, 1H, H-C(14)), 6.76 (d, *J*(H,H)=8.8 Hz, 1H, H-C(13)), 3.84 (s, 3H, H-C(16)), 3.76 (s, 3H, H-C(12)), 3.07 (d, *J*(H,H)=15.4 Hz, 1H, H-C(18)), 2.90 (d, *J*(H,H)=15.4 Hz, 1H, H-C(18′)), 1.95 (s, 3H, H-C(21)), 1.38 (s, 3H, H-C(20)); ^13^C-NMR (201 MHz, CD_2_Cl_2_): *δ* 182.7 (C(1)), 169.2 (C(9)), 153.0 (C(11)), 149.8 (C(15)), 149.2 (C(7)), 140.0 (C(8)), 137.4 (C(17)), 135.5 (C(2)), 134.7 (C(5)), 132.3 (C(4)), 128.7 (C(10)), 126.8 (C(6)), 124.8 (C(3)), 115.7 (C(14)), 109.6 (C(13)), 56.0 (C(16)), 55.1 (C(12)), 52.3 (C(19)), 46.3 (C(18)), 28.1 (C(21)), 25.8 (C(20)); IR (): 3419*m*, 2954*m*, 2834*vw*, 2780*vw*, 2666*w*, 2568*vw*, 2512*vw*, 2471*vw*, 2155*vw*, 1675*vs*, 1588*m*, 1548*m*, 1491*s*, 1465*s*, 1451*s*, 1437*m*, 1412*m*, 1384*m*, 1363*w*, 1334*m*, 1317*m*, 1262*s*, 1215*s*, 1172*m*, 1163*m*, 1137*w*, 1118*m*, 1097*m*, 1070*s*, 1037*s*, 1002*s*, 959*s*, 863*m*, 797*m*, 783*m*, 753*vs*, 711*s*, 668*s*, 668*vs* cm^–1^; ultraviolet/vis (CH_2_Cl_2_): *λ*_max_ (*ɛ*) 420 (3,700 l mol^–1 ^cm^–1^), 341 (12,200 l mol^–1 ^cm^–1^) nm. For further experimental details and characterization data, see Supplementary Methods.

### Calculations

The details of the theoretical description of motor **1** are given in the Supplementary Methods and all calculated stationary points are provided as Supplementary cif-files. The geometries of the *Z*-(*S*)-(*P*)/*Z*-(*R*)-(*M*) and *E*-(*S*)-(*P*)/*E*-(*R*)-(*M*) isomers found in the corresponding crystal structures are compared to the theoretically obtained geometries in Supplementary Table 4.

## Additional information

**Accession codes:** The X-ray crystallographic coordinates for structures reported in this study have been deposited at the Cambridge Crystallographic Data Centre (CCDC), under CCDC numbers 1061969, 1061970, 1406625, 1408096 and 1408097. These data can be obtained free of charge from the Cambridge Crystallographic Data Centre via www.ccdc.cam.ac.uk/data_request/cif.

**How to cite this article:** Guentner, M. *et al*. Sunlight-powered kHz rotation of a hemithioindigo-based molecular motor. *Nat. Commun*. 6:8406 doi: 10.1038/ncomms9406 (2015).

## Supplementary Material

Supplementary Information Supplementary Figures 1-41, Supplementary Tables 1-4, Supplementary Methods and Supplementary References.

Supplementary Dataset 1 MPW1K_Minimum_ESM_Isomer.cif = minimum structure of the E-(S)-(M) isomer of motor 1 calculated at the MPW1K level of theory using a 6-31+G(d,p) basis set

Supplementary Dataset 2 MPW1K_Minimum_ESP_Isomer.cif = minimum structure of the E-(S)-(P) isomer of motor 1 calculated at the MPW1K level of theory using a 6-31+G(d,p) basis set

Supplementary Dataset 3 MPW1K_Minimum_ZSM_Isomer.cif = minimum structure of the Z-(S)-(M) isomer of motor 1 calculated at the MPW1K level of theory using a 6-31+G(d,p) basis set

Supplementary Dataset 4 MPW1K_Minimum_ZSP_Isomer.cif = minimum structure of the Z-(S)-(P) isomer of motor 1 calculated at the MPW1K level of theory using a 6-31+G(d,p) basis set

Supplementary Dataset 5 MPW1K_TransitionState_ESM_ESP.cif = transition state structure of the conversion from E-(S)-(M) to E-(S)-(P) isomer of motor 1 calculated at the MPW1K level of theory using a 6-31+G(d,p) basis set

Supplementary Dataset 6 MPW1K_TransitionState_ZSM_ZSP.cif = transition state structure of the conversion from Z-(S)-(M) to Z-(S)-(P) isomer of motor 1 calculated at the MPW1K level of theory using a 6-31+G(d,p) basis set

## References

[b1] Koumura N., Zijlstra R. W. J., van Delden R. A. & Feringa B. L. Light-driven monodirectional molecular rotor. Nature 401, 152–155 (1999). 10490022 10.1038/43646

[b2] Hernandez J. V., Kay E. R. & Leigh D. A. A reversible synthetic rotary molecular motor. Science 306, 1532–1537 (2004). 15567858 10.1126/science.1103949

[b3] Greb L. & Lehn J. M. Light-driven molecular motors: imines as four-step or two-step unidirectional rotors. J. Am. Chem. Soc. 136, 13114–13117 (2014). 25211628 10.1021/ja506034n

[b4] Ragazzon G., Baroncini M., Silvi S., Venturi M. & Credi A. Light-powered autonomous and directional molecular motion of a dissipative self-assembling system. Nat. Nanotechnol. 10, 70–75 (2015). 25420035 10.1038/nnano.2014.260

[b5] Kopelman R. Racing with nature: artificial nanomachines that keep running on light, both left and right. ACS Nano 6, 7553–7555 (2012). 22974388 10.1021/nn304119h

[b6] Astumian R. D. Thermodynamics and kinetics of a Brownian motor. Science 276, 917–922 (1997). 9139648 10.1126/science.276.5314.917

[b7] Mandl C. P. & König B. Chemistry in motion—unidirectional rotating molecular motors. Angew. Chem. Int. Ed. 43, 1622–1624 (2004). 10.1002/anie.200301697 15038026

[b8] Kay E. R., Leigh D. A. & Zerbetto F. Synthetic molecular motors and mechanical machines. Angew. Chem. Int. Ed. 46, 72–191 (2007). 10.1002/anie.200504313 17133632

[b9] Balzani V., Credi A. & Venturi M. Molecular Devices and Machines—Concepts and Perspectives for the Nanoworld Wiley-VCH (2008).

[b10] Browne W. R. & Feringa B. L. Making molecular machines work. Nat. Nanotechnol. 1, 25–35 (2006). 18654138 10.1038/nnano.2006.45

[b11] Coskun A., Banaszak M., Astumian R. D., Stoddart J. F. & Grzybowski B. A. Great expectations: can artificial molecular machines deliver on their promise? Chem. Soc. Rev. 41, 19–30 (2012). 22116531 10.1039/c1cs15262a

[b12] Kelly T. R., De Silva H. & Silva R. A. Unidirectional rotary motion in a molecular system. Nature 401, 150–152 (1999). 10490021 10.1038/43639

[b13] Wang J. & Feringa B. L. Dynamic control of chiral space in a catalytic asymmetric reaction using a molecular motor. Science 331, 1429–1432 (2011). 21310964 10.1126/science.1199844

[b14] Li Q. . Macroscopic contraction of a gel induced by the integrated motion of light-driven molecular motors. Nat. Nanotechnol. 10, 161–165 (2015). 25599191 10.1038/nnano.2014.315

[b15] Zhao D., Neubauer T. M. & Feringa B. L. Dynamic control of chirality in phosphine ligands for enantioselective catalysis. Nat. Commun. 6, 6652 (2015). 25806856 10.1038/ncomms7652 PMC4389239

[b16] Eelkema R. . Molecular machines: nanomotor rotates microscale objects. Nature 440, 163 (2006). 16525460 10.1038/440163a

[b17] Kinosita K. Jr., Adachi K. & Itoh H. Rotation of F1-ATPase: how an ATP-driven molecular machine may work. Annu. Rev. Biophys. Biomol. Struct. 33, 245–268 (2004). 15139813 10.1146/annurev.biophys.33.110502.132716

[b18] Boyer P. D. Energy, life, and ATP (Nobel Lecture). Angew. Chem. Int. Ed. 37, 2296–2307 (1998). 10.1002/(SICI)1521-3773(19980918)37:17<2296::AID-ANIE2296>3.0.CO;2-W 29710952

[b19] Walker J. E. ATP synthesis by rotary catalysis (Nobel lecture). Angew. Chem. Int. Ed. 37, 2308–2319 (1998). 10.1002/(SICI)1521-3773(19980918)37:17<2308::AID-ANIE2308>3.0.CO;2-W 29710950

[b20] Klok M. . MHz unidirectional rotation of molecular rotary motors. J. Am. Chem. Soc. 130, 10484–10485 (2008). 18636709 10.1021/ja8037245

[b21] Vachon J. . An ultrafast surface-bound photo-active molecular motor. Photochem. Photobiol. Sci. 13, 241–246 (2014). 24096390 10.1039/c3pp50208b

[b22] Michl J. & Sykes E. C. H. Molecular rotors and motors: recent advances and future challenges. ACS Nano 3, 1042–1048 (2009). 19845364 10.1021/nn900411n

[b23] van Delden R. A., Koumura N., Schoevaars A., Meetsma A. & Feringa B. L. A donor–acceptor substituted molecular motor: unidirectional rotation driven by visible light. Org. Biomol. Chem. 1, 33–35 (2003). 12929386 10.1039/b209378b

[b24] Cnossen A. . Driving unidirectional molecular rotary motors with visible light by intra- and intermolecular energy transfer from palladium porphyrin. J. Am. Chem. Soc. 134, 17613–17619 (2012). 23036108 10.1021/ja306986g

[b25] Friedländer P. Ueber schwefelhaltige Analoga der Indigogruppe. Chem. Ber. 39, 1060–1066 (1906).

[b26] Wiedbrauk S. & Dube H. Hemithioindigo—an emerging photoswitch. Tetrahedron Lett. 56, 4266–4274 (2015).

[b27] Seki T., Tamaki T., Yamaguchi T. & Ichimura K. Photochromism of hemithioindigo derivatives. II. Photochromic behavior in bilayer membranes and related systems. Bull. Chem. Soc. Jpn. 65, 657–663 (1992).

[b28] Eggers K., Fyles T. M. & Montoya-Pelaez P. J. Synthesis and characterization of photoswitchable lipids containing hemithioindigo chromophores. J. Org. Chem. 66, 2966–2977 (2001). 11325261 10.1021/jo0056848

[b29] Lougheed T., Borisenko V., Hennig T., Rück-Braun K. & Woolley G. A. Photomodulation of ionic current through hemithioindigo-modified gramicidin channels. Org. Biomol. Chem. 2, 2798–2801 (2004). 15455152 10.1039/B408485C

[b30] Regner N. . Light-switchable hemithioindigo-hemistilbene-containing peptides: ultrafast spectroscopy of the Z—>E isomerization of the chromophore and the structural dynamics of the peptide moiety. J. Phys. Chem. B 116, 4181–4191 (2012). 22423868 10.1021/jp300982a

[b31] Herre S. . Photoactivation of an inhibitor of the 12/15-lipoxygenase pathway. ChemBioChem 7, 1089–1095 (2006). 16755628 10.1002/cbic.200600082

[b32] Tanaka K., Kohayakawa K., Iwata S. & Irie T. Application of 2-pyridyl-substituted hemithioindigo as a molecular switch in hydrogen-bonded porphyrins. J. Org. Chem. 73, 3768–3774 (2008). 18429634 10.1021/jo800091d

[b33] Dube H. & Rebek J. Jr. Selective guest exchange in encapsulation complexes using light of different wavelenghts. Angew. Chem. Int. Ed. 51, 3207–3210 (2012). 10.1002/anie.201108074 22337589

[b34] Cordes T., Schadendorf T., Priewisch B., Rück-Braun K. & Zinth W. The Hammett relationship and reactions in the excited electronic state: hemithioindigo *Z*/*E*-photoisomerization. J. Phys. Chem. A 112, 581–588 (2008). 18177026 10.1021/jp077472l

[b35] Cordes T., Schadendorf T., Rück-Braun K. & Zinth W. Chemical control of hemithioindigo-photoisomerization—substituent-effects on different molecular parts. Chem. Phys. Lett. 455, 197–201 (2008).

[b36] Izmail'skii V. A. & Mostoslavskii M. A. Absorption spectra of 3-oxo-2,3-dihydrothianaphthene and its derivatives. II. Isomerism of 2-benzylidene-3-oxo-2,3-dihydrothionaphthene. Ukr. Khem. Zh. 27, 234–237 (1961).

[b37] Reamonn L. S. S. & O'Sullivan W. I. Configuration of 2-arylmethylene-2,3-di hydro-5-methyl benzo[*b*]thiophen-3-ones. J. Chem. Soc. Perkin Trans. 1, 1009–1012 (1977).

[b38] Pollard M. M., Klok M., Pijper D. & Feringa B. L. Rate acceleration of light-driven rotary molecular motors. Adv. Funct. Mater. 17, 718–729 (2007).

[b39] Zarei M. & Mohamadzadeh M. 3- Thiolated 2-azetidinones: synthesis and in vitro antibacterial and antifungal activitie. Tetrahedron 67, 5832–5840 (2011).

[b40] Maerz B. . Making fast photoswitches faster—using Hammett analysis to understand the limit of donor-acceptor approaches for faster hemithioindigo photoswitches. Chem. Eur. J. 20, 13984–13992 (2014). 25214477 10.1002/chem.201403661

[b41] Cnossen A., Kistemaker J. C., Kojima T. & Feringa B. L. Structural dynamics of overcrowded alkene-based molecular motors during thermal isomerization. J. Org. Chem. 79, 927–935 (2014). 24410498 10.1021/jo402301j

[b42] Morin F. G., Horton W. J., Grant D. M. & Pugmire R. J. Carbon-13 magnetic resonance of hydroaromatics. 3. Conformation of 1,2,3,4-tetrahydrophenanthrene and 9,10-dihydrophenanthrene and their methyl derivatives. J. Org. Chem. 50, 3380–3388 (1985).

