# Supplementary material for: Sunlight-powered kHz rotation of a hemithioindigo-based molecular motor
Source: Nat Commun. 2015 Sep 28;6:8406. doi: 10.1038/ncomms9406 (PMC4598625; doi:10.1038/ncomms9406)
Supplement: Supplementary Information — Supplementary Figures 1-41, Supplementary Tables 1-4, Supplementary Methods and Supplementary References. [file ncomms9406-s1.pdf]

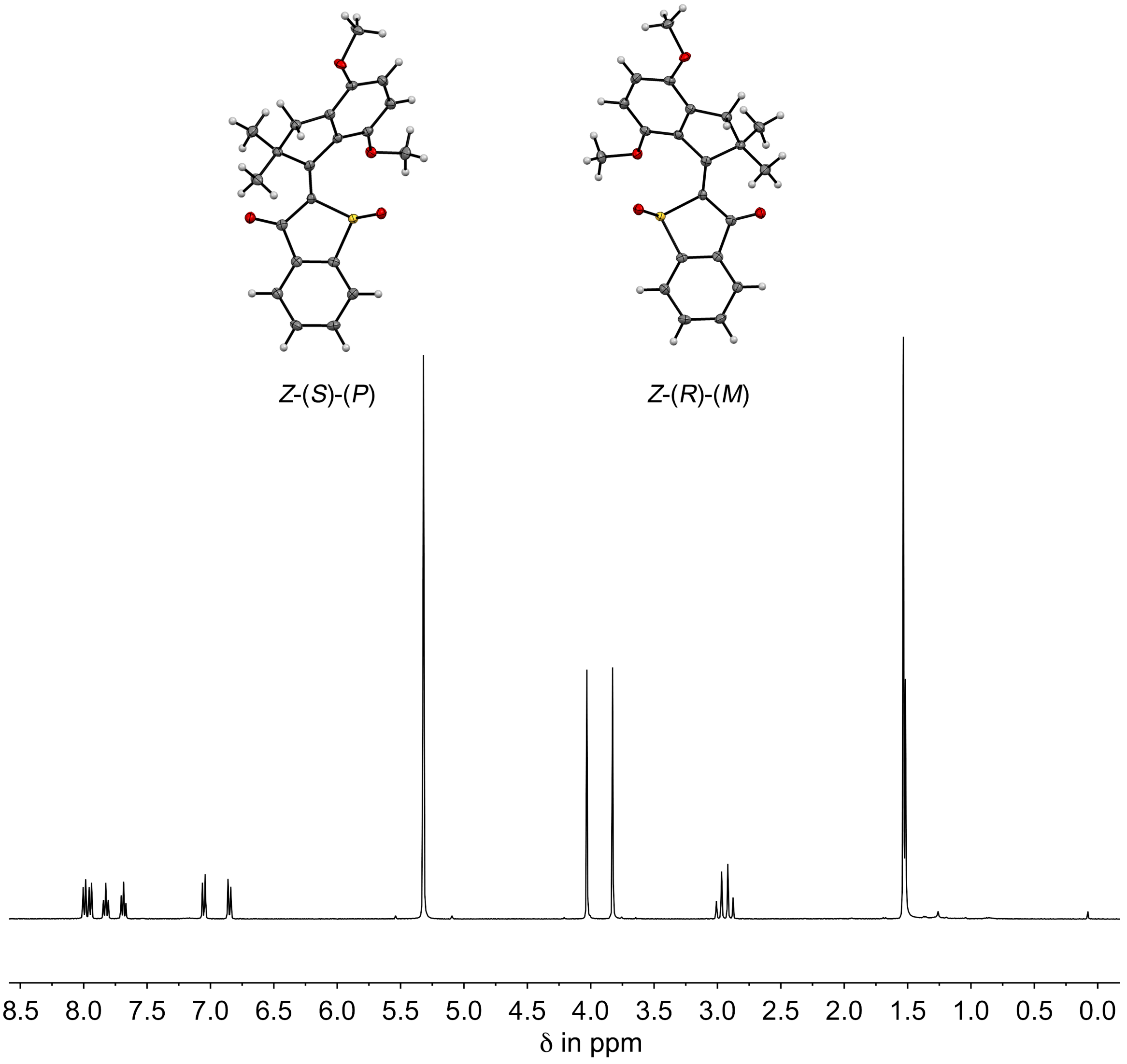

Supplementary Figure 1 | Crystal structures of the racemic *Z*-(*S*)-(*P*) and *Z*-(*R*)-(*M*) isomers of motor 1 and the corresponding ^{1}H NMR spectrum (CD_2Cl_2, 400 MHz, 27 °C) of the same crystal batch. The signals of only one single species are observed in the ^{1}H NMR spectrum, which could thus be directly assigned to the *Z*-(*S*)-(*P*)/*Z*-(*R*)-(*M*) isomers.

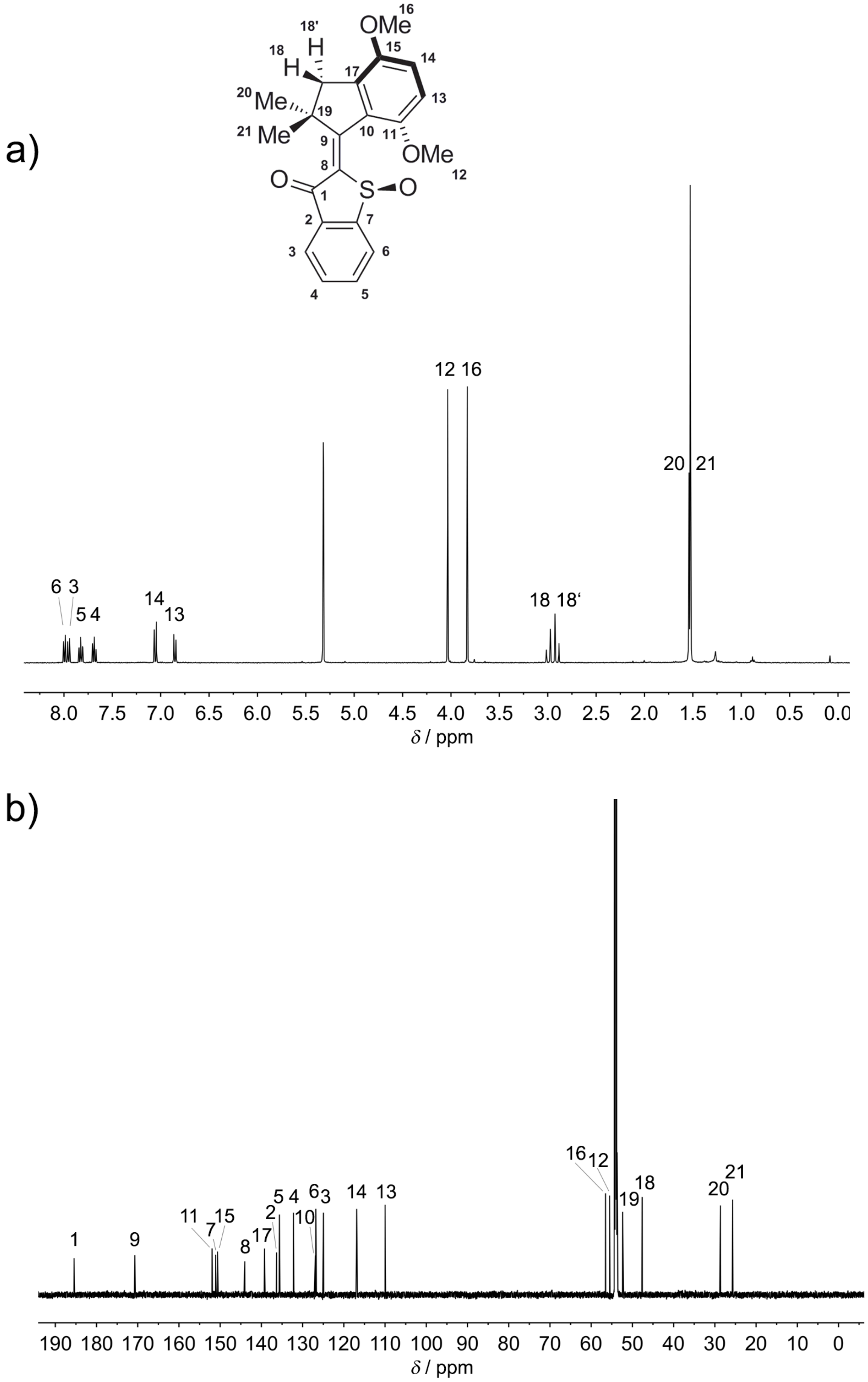

Supplementary Figure 2 | NMR spectra of the *Z*-(*S*)-(*P*)/*Z*-(*R*)-(*M*) isomers of motor 1 (CD_2Cl_2, 800 MHz, 27 °C) and assignments of the signals to the molecular structure of the *Z*-(*S*)-(*P*) isomer. a) ^{1}H NMR spectrum. b) ^{13}C NMR spectrum.

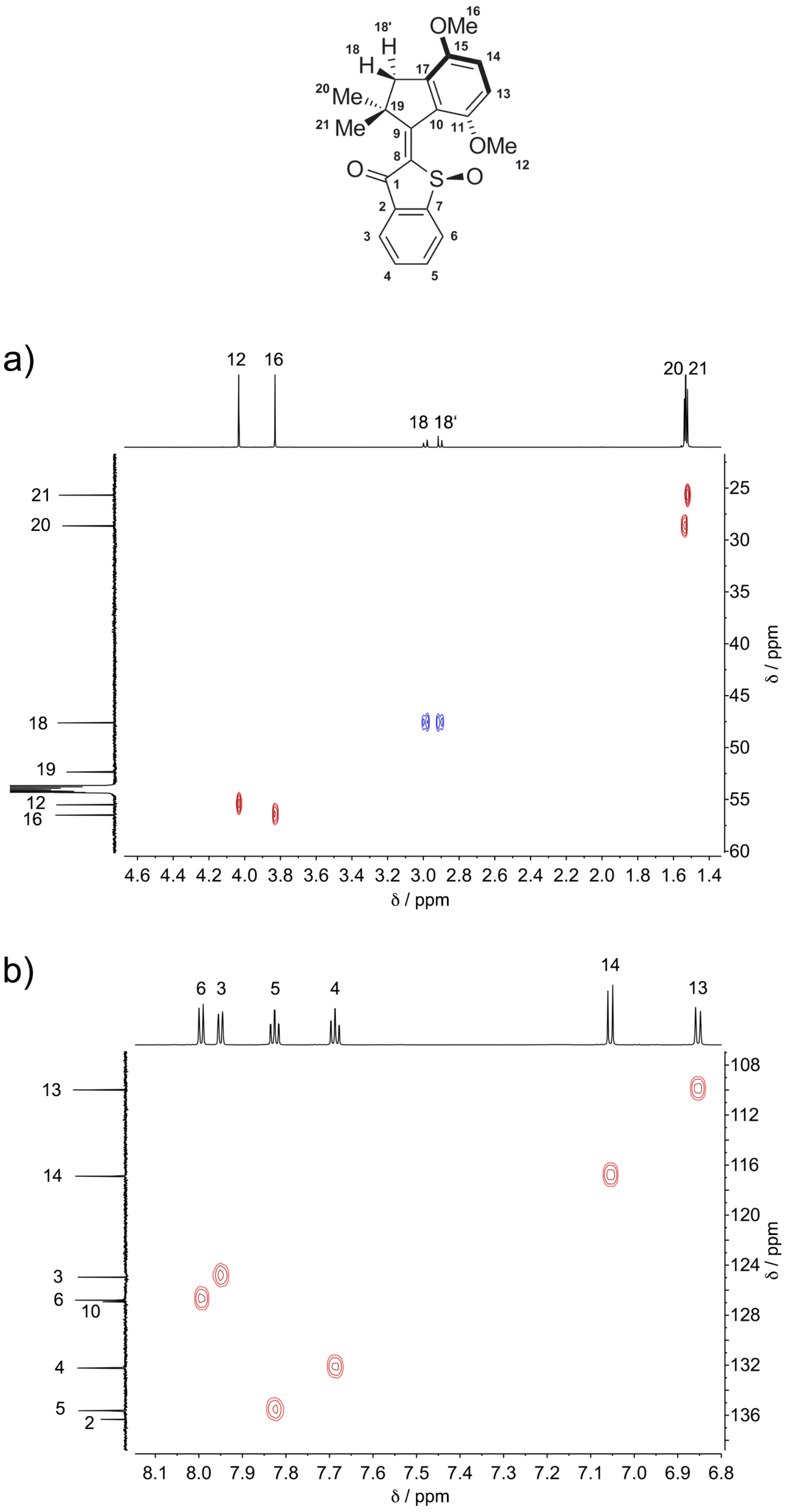

Supplementary Figure 3 | HSQC NMR spectrum of the *Z*-(*S*)-(*P*)/*Z*-(*R*)-(*M*) isomers of motor 1 (CD_2Cl_2, 800 MHz, 27 °C) and assignments of the signals to the molecular structure of the *Z*-(*S*)-(*P*) isomer. a) Aliphatic part of the spectrum. b) Aromatic part of the spectrum.

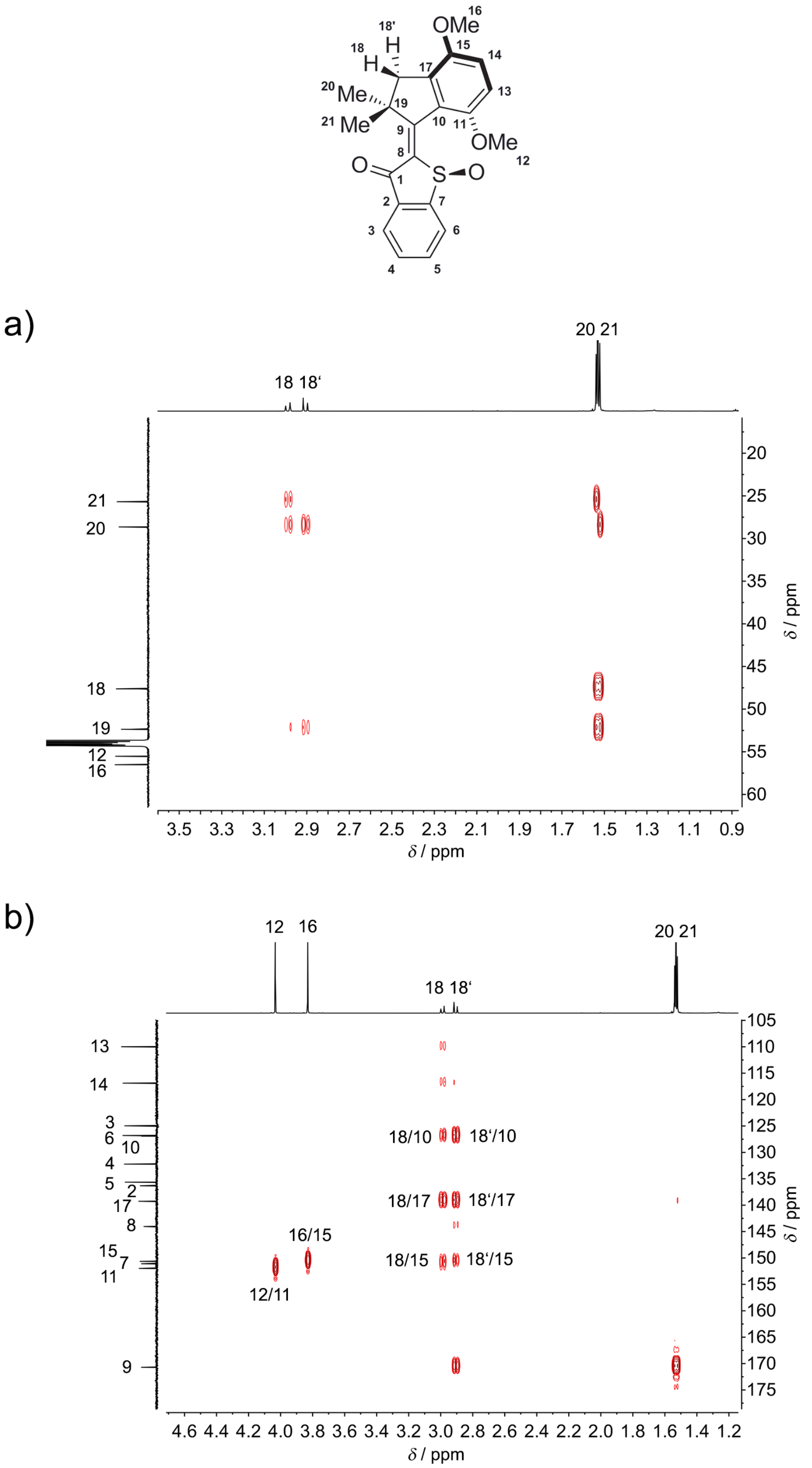

Supplementary Figure 4 | HMBC NMR spectrum of the *Z*-(*S*)-(*P*)/*Z*-(*R*)-(*M*) isomers of motor 1 (CD_2Cl_2, 800 MHz, 27 °C) and assignments of signals to the molecular structure of the *Z*-(*S*)-(*P*) isomer. a) Aliphatic part of the spectrum. The cross peak between proton 18’ and carbon 21 is not seen because the C(21)-C(19)-C(18)-H(18’) dihedral angle is close to 90.0°, similar to the 93.5° of this angle observed in the crystal structure of *Z*-(*S*)-(*P*)-**1**. b) Aliphatic-aromatic part of the spectrum. The cross peak between proton 18 and carbon 9 is not seen because the C(9)-C(19)-C(18)-H(18) dihedral angle is close to 90.0°, similar to the 96.6° of this angle observed in the crystal structure of *Z*-(*S*)-(*P*)-**1**.

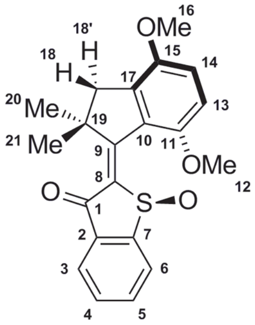

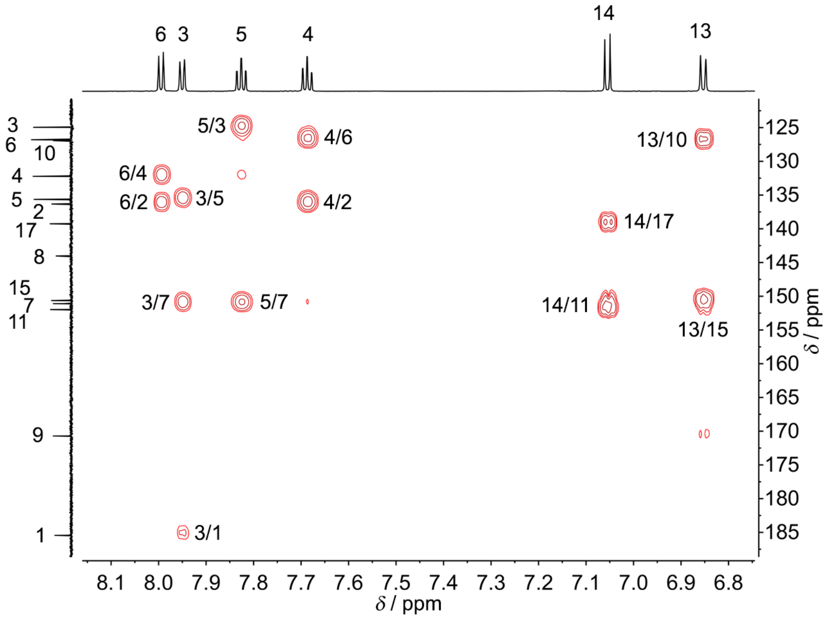

Supplementary Figure 5 | HMBC NMR spectrum of the *Z*-(*S*)-(*P*)/*Z*-(*R*)-(*M*) isomers of motor 1 (CD_2Cl_2, 800 MHz, 27 °C) and assignments of signals to the molecular structure of the *Z*-(*S*)-(*P*) isomer. The aromatic part of the spectrum is shown.

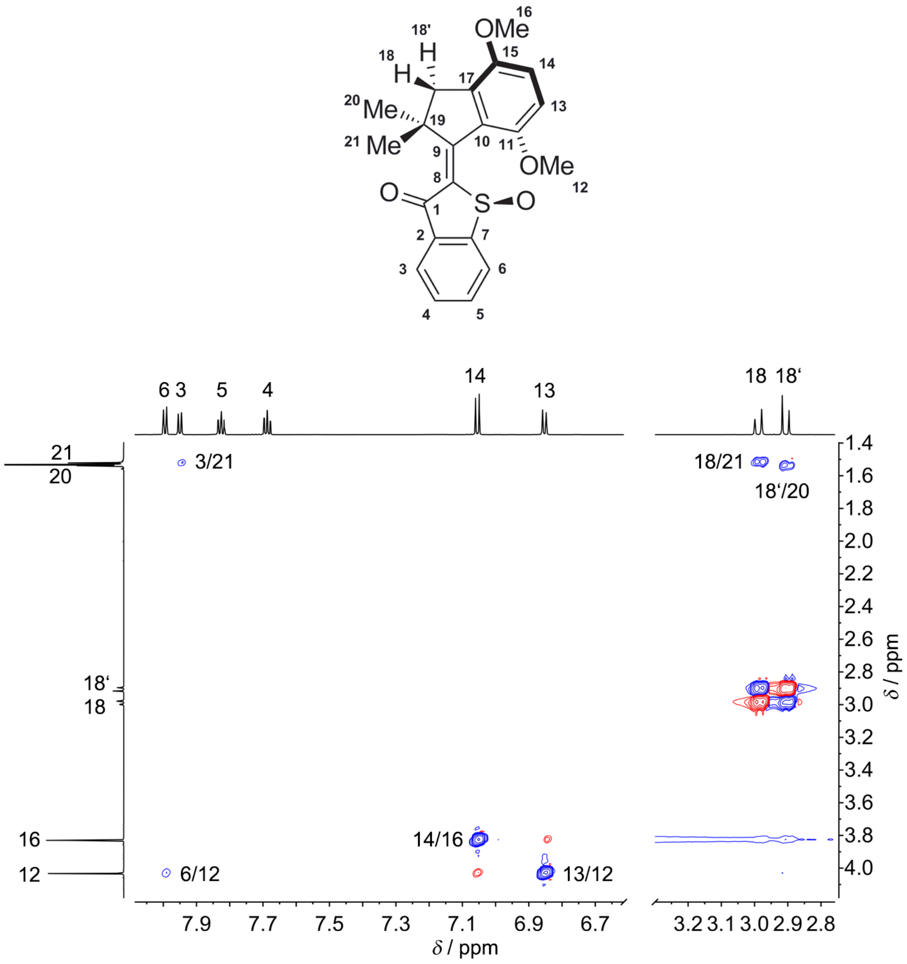

Supplementary Figure 6 | NOESY NMR spectrum of the *Z*-(*S*)-(*P*)/*Z*-(*R*)-(*M*) isomers of motor 1 (CD_2Cl_2, 800 MHz, 27 °C, 3.6 s mixing time) and assignments of signals to the molecular structure of the *Z*-(*S*)-(*P*) isomer. Indicative signals proving the *Z* configuration of the double bond are marked.

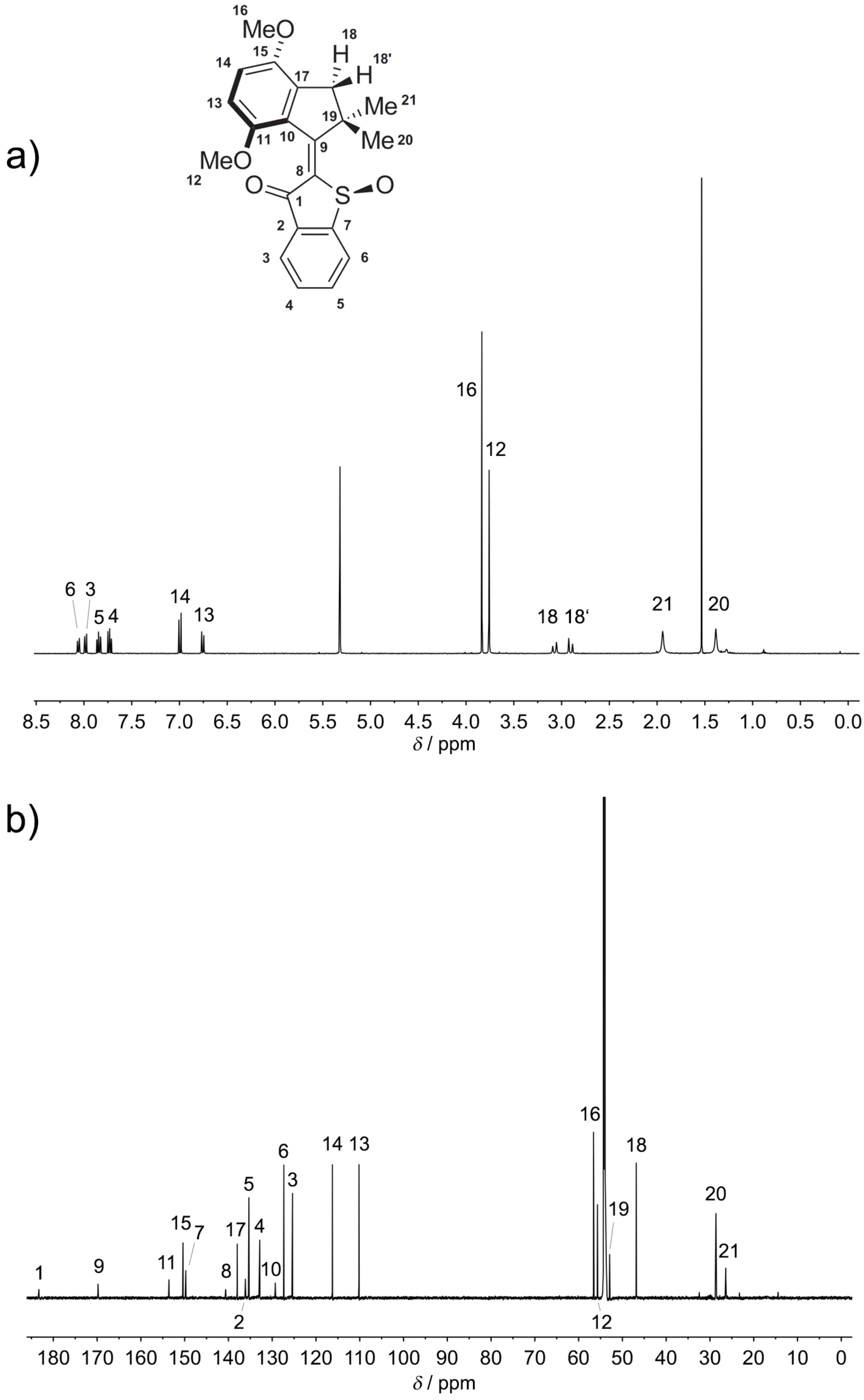

Supplementary Figure 7 | NMR spectra of the *E*-(*S*)-(*P*)/*E*-(*R*)-(*M*) isomers of motor 1 (CD_2Cl_2, 800 MHz, 27 °C) and assignments of the signals to the molecular structure of the *E*-(*S*)-(*P*) isomer. a) ^{1}H NMR spectrum. b) ^{13}C NMR spectrum.

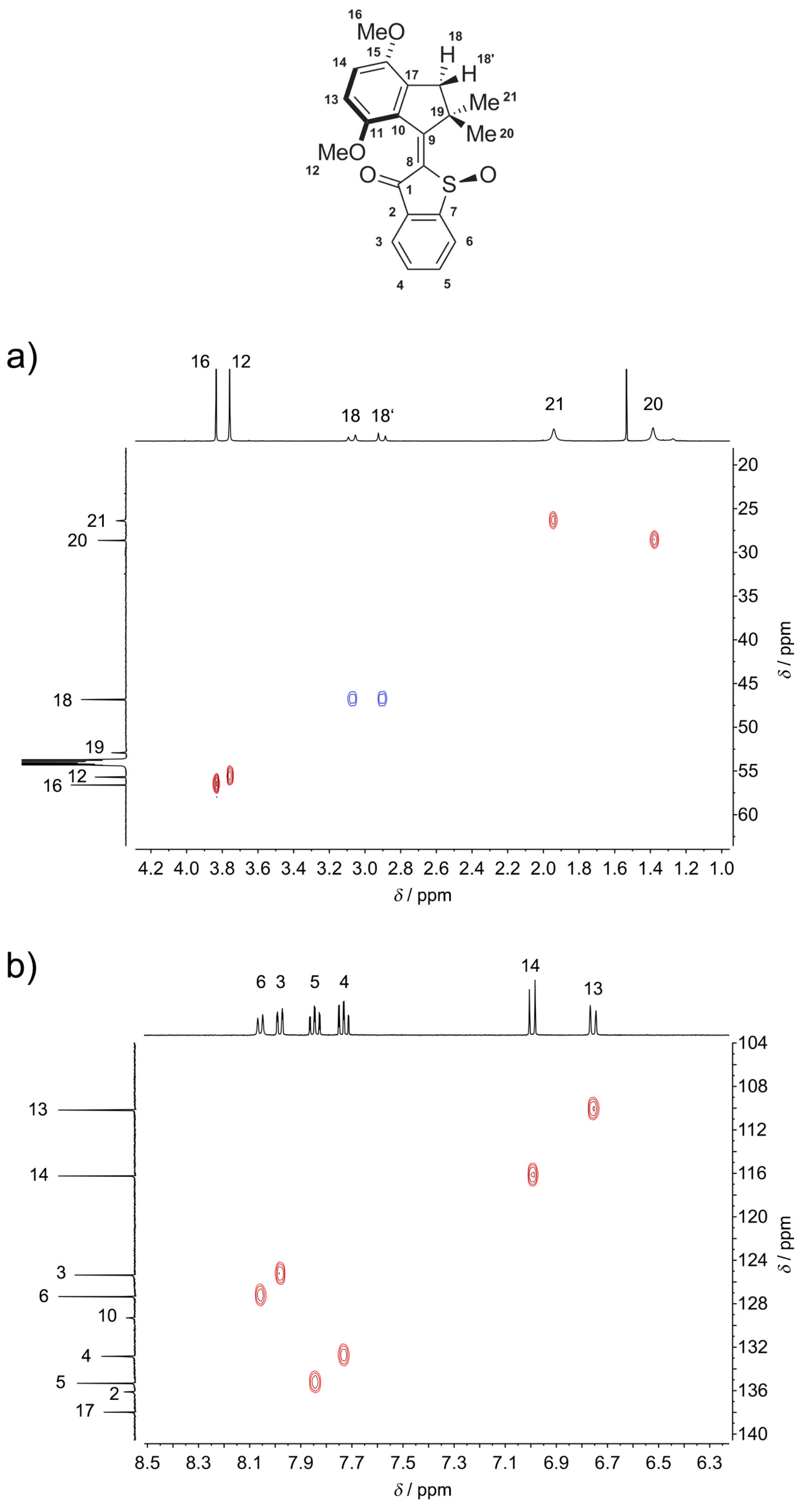

Supplementary Figure 8 | HSQC NMR spectrum of the *E*-(*S*)-(*P*)/*E*-(*R*)-(*M*) isomers of motor 1 (CD_2Cl_2, 600 MHz, 27 °C) and assignments of the signals to the molecular structure of the *E*-(*S*)-(*P*) isomer.
a) Aliphatic part of the spectrum. b) Aromatic part of the spectrum.

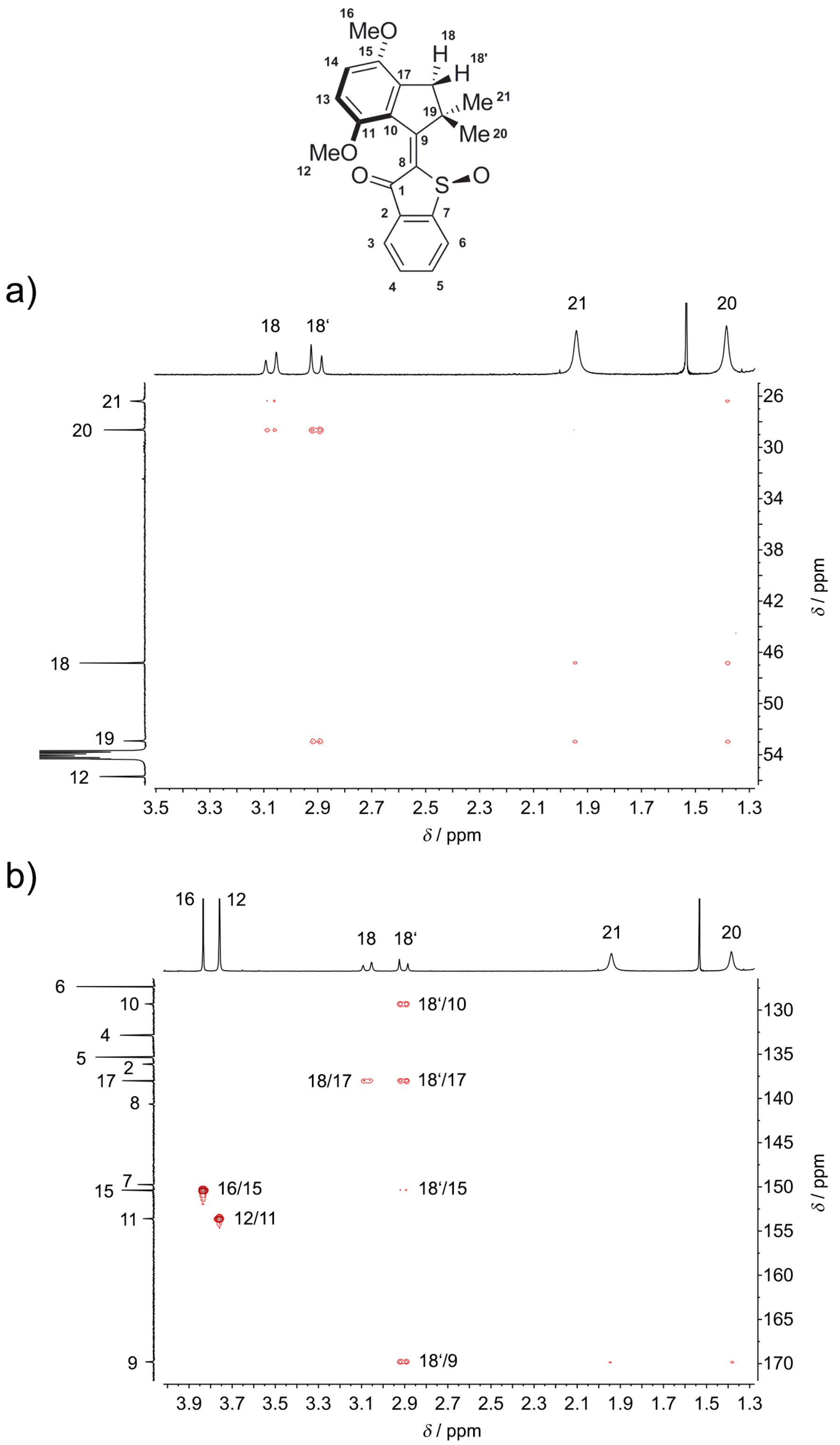

Supplementary Figure 9 | HMBC NMR spectrum of the *E*-(*S*)-(*P*)/*E*-(*R*)-(*M*) isomers of motor 1 (CD_2Cl_2, 600 MHz, 27 °C) and assignments of signals to the molecular structure of the *E*-(*S*)-(*P*) isomer. a) Upfield aliphatic part of the spectrum. The cross peak between proton 18' and carbon 21 is not seen because the C(21)-C(19)-C(18)-H(18') dihedral angle is close to 90.0°, similar to the 86.0° of this angle observed in the crystal structure of *E*-(*S*)-(*P*)-**1**. b) Aliphatic-aromatic part of the spectrum. The cross peaks between proton 18 and carbon 9, between proton 18 and carbon 15, and between proton 18 and carbon 10 are not seen because the C(9)-C(19)-C(18)-H(18), C(15)-C(17)-C(18)-H(18), and C(10)-C(17)-C(18)-H(18) dihedral angles are close to 90.0°, similar to the 90.2°, 79.7°, and 102.5° of the corresponding angles observed in the crystal structure of *E*-(*S*)-(*P*)-**1**.

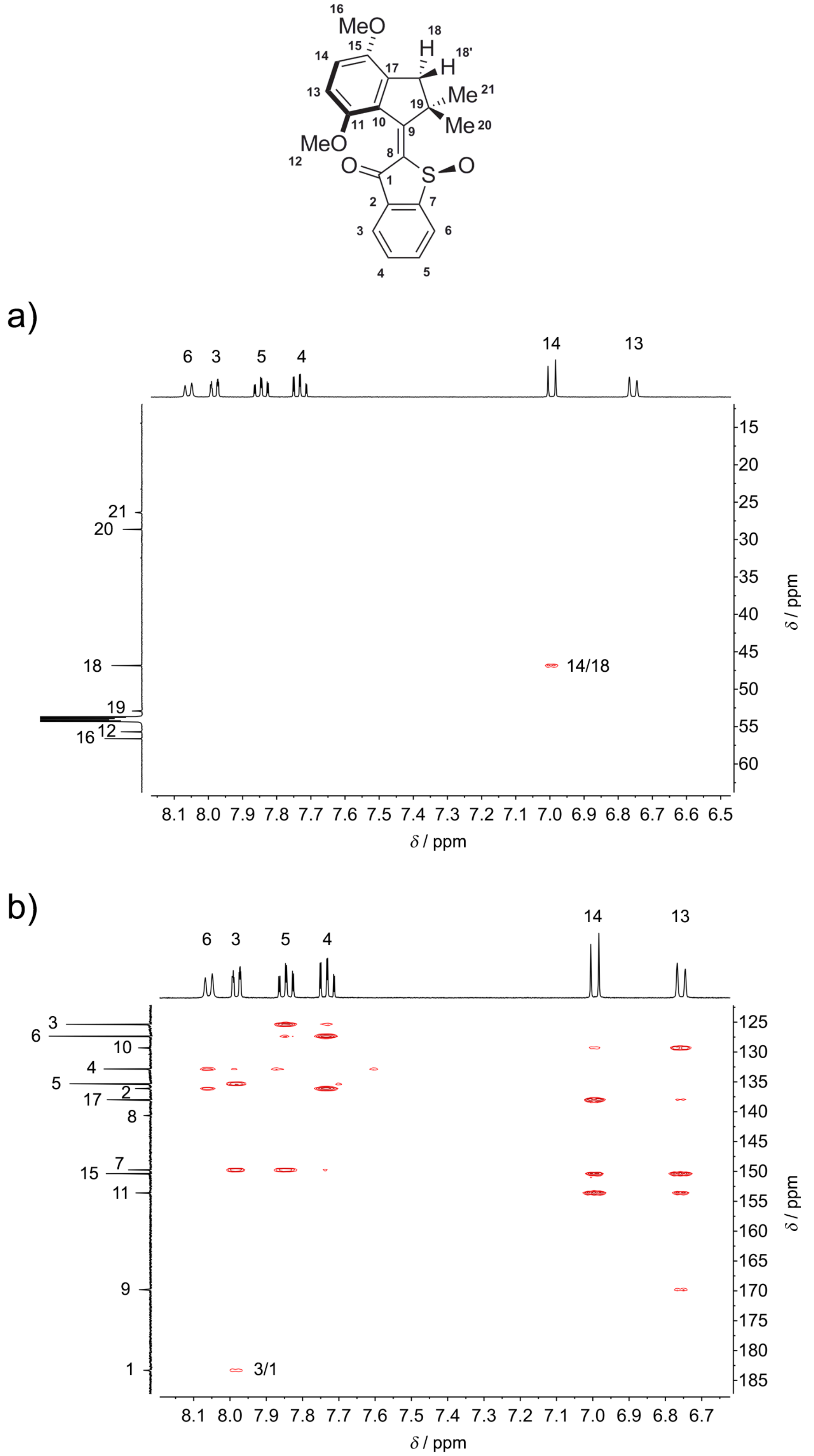

Supplementary Figure 10 | HMBC NMR spectrum of the *E*-(*S*)-(*P*)/*E*-(*R*)-(*M*) isomers of motor 1 (CD_2Cl_2, 600 MHz, 27 °C) and assignments of signals to the molecular structure of the *E*-(*S*)-(*P*) isomer. a) Upfield aromatic part of the spectrum. b) Lowfield aromatic part of the spectrum.

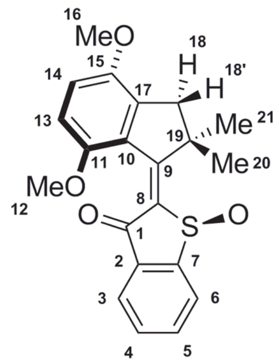

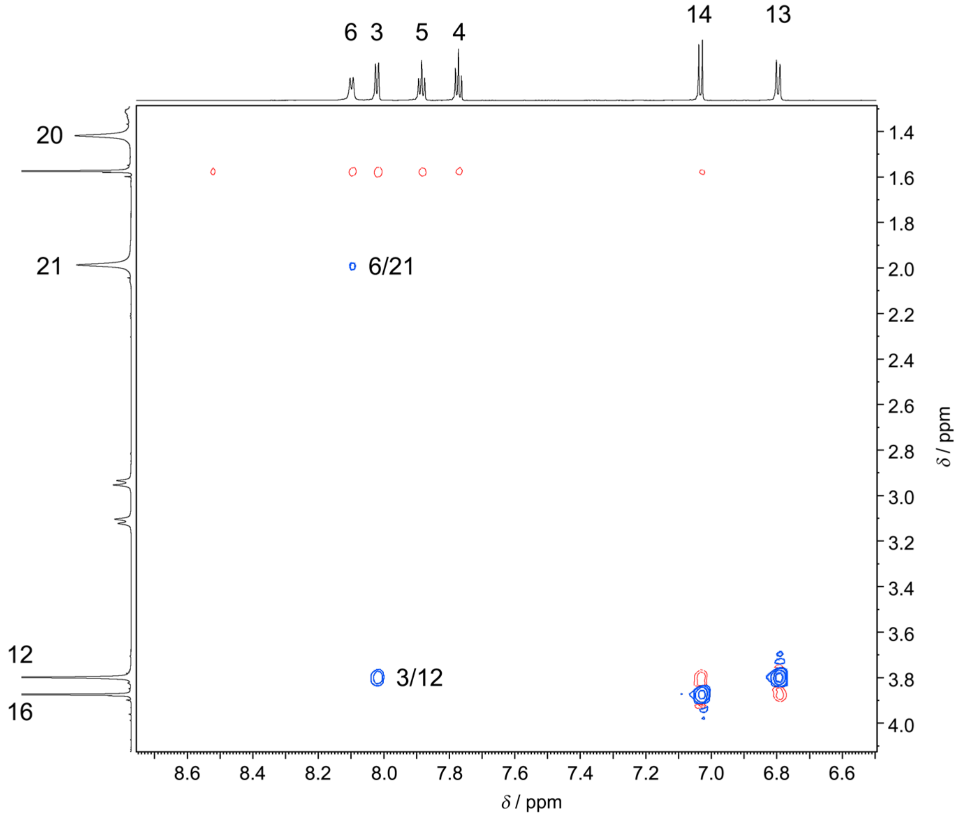

Supplementary Figure 11 | NOESY NMR spectrum of the *E*-(*S*)-(*P*)/*E*-(*R*)-(*M*) isomers of motor 1 (CD_2Cl_2, 800 MHz, 27 °C, 3.6 s mixing time) and assignments of signals to the molecular structure of the *E*-(*S*)-(*P*) isomers. Indicative signals proving the *E* configuration of the double bond are marked.

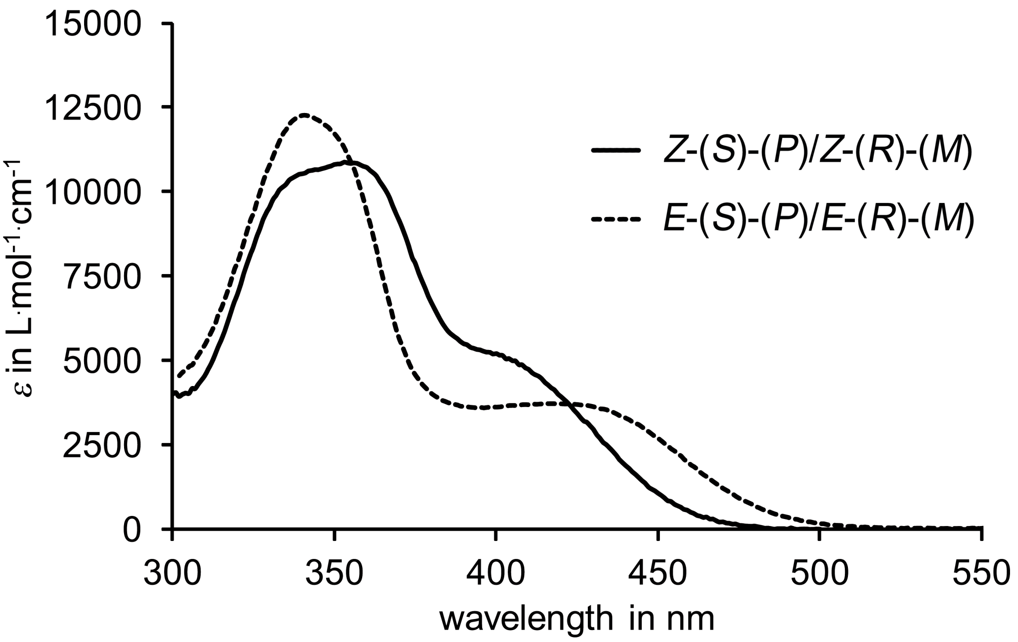

Supplementary Figure 12 | Measured extinction coefficients *ε* for the *Z*-(*S*)-(*P*)/*Z*-(*R*)-(*M*) (solid line) and *E*-(*S*)-(*P*)/*E*-(*R*)-(*M*) isomers (dashed line) of motor 1 in CH_2Cl_2.

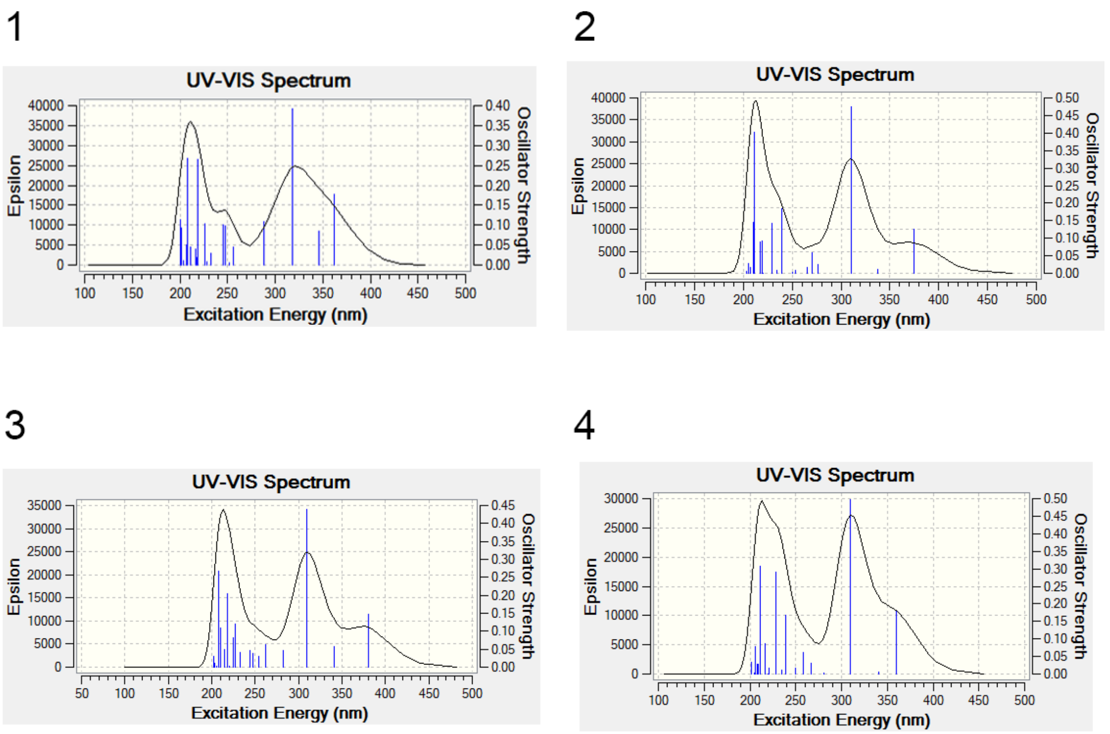

Supplementary Figure 13 | Theoretical extinction coefficients of motor 1 in CH_2Cl_2 calculated at the MPW1K level of theory using the 6-311++G(d,p) basis set. The spectra are blueshifted (ca. 50 nm) compared to the experimentally obtained extinction coefficients. 1: *Z*-(*S*)-(*P*) isomer of motor **1**. The main transition is HOMO–1 to LUMO for the long wavelength parts of the spectrum. 2: *E*-(*S*)-(*M*) isomer of motor **1**. 3: *E*-(*S*)-(*P*) isomer of motor **1**. The main transition is HOMO to LUMO for the long wavelength parts of the spectrum. 4: *Z*-(*S*)-(*M*) isomer of motor **1**.

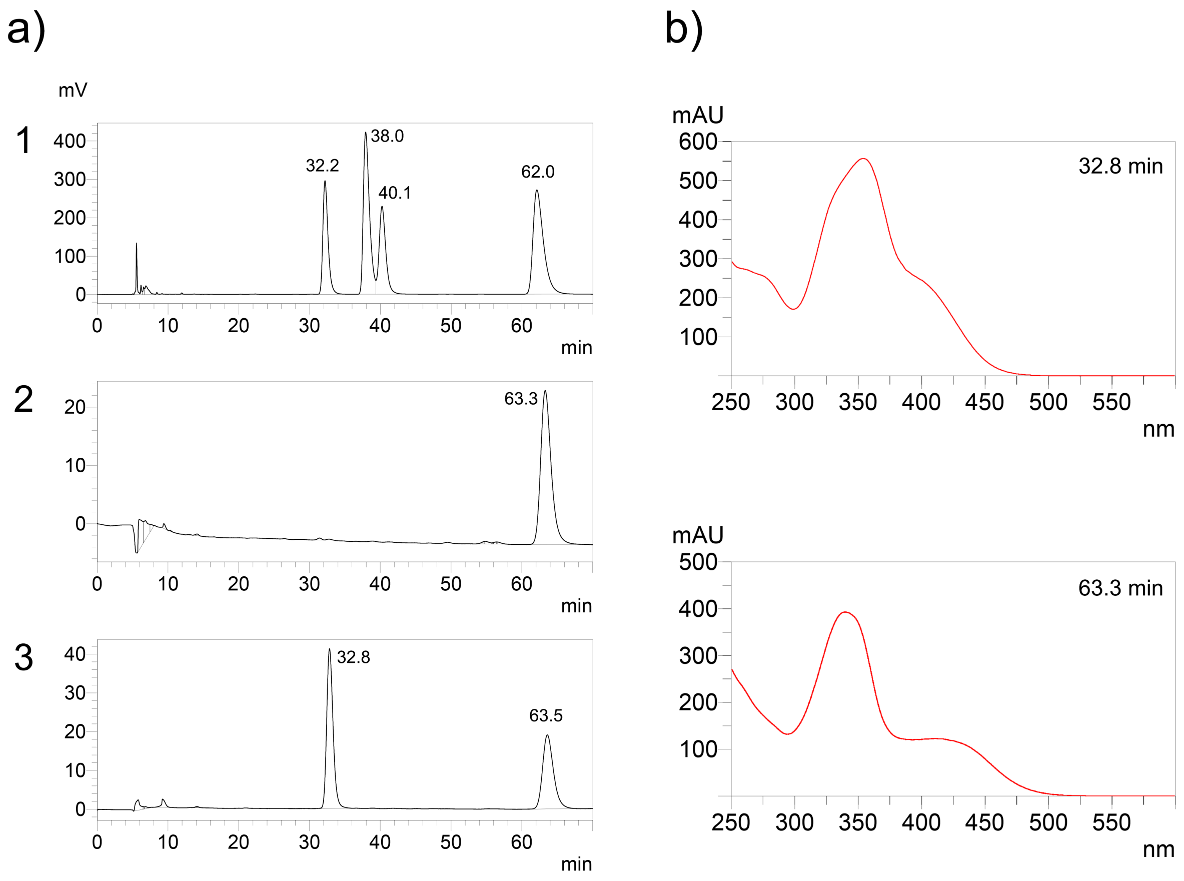

Supplementary Figure 14 | Racemization test of motor 1 under irradiation conditions. a) HPLC traces obtained from separating isomeric mixtures of motor **1** using a CHIRALPAK® IC column from Diacel (*n*-heptane : 2-PrOH = 8 : 2, at 40 °C). 1: Racemic *Z* and *E* mixture of motor **1**. All four thermally stable isomers (*Z*-(*S*)-(*P*), *Z*-(*R*)-(*M*), *E*-(*S*)-(*P*), and *E*-(*R*)-(*M*)) are separated. 2: Isolated enantiomerically pure *E* isomer (retention time 63.3 min). 3: After irradiating the isolated enantiomerically pure *E* isomer with 470 nm light only the corresponding enantiomerically pure *Z* isomer (retention time 32.8 min) is formed. b) Top: UV/Vis spectrum of the *Z* enantiomer eluted after 32.8 min. The UV/Vis spectrum of the opposite *Z* enantiomer eluted after 40.1 min is exactly the same and not shown here. Bottom: The UV/Vis spectrum of the *E* enantiomer eluted after 63.3 min (bottom). The UV/Vis spectrum of the opposite *E* enantiomer eluted after 38.0 min is exactly the same and not shown here.

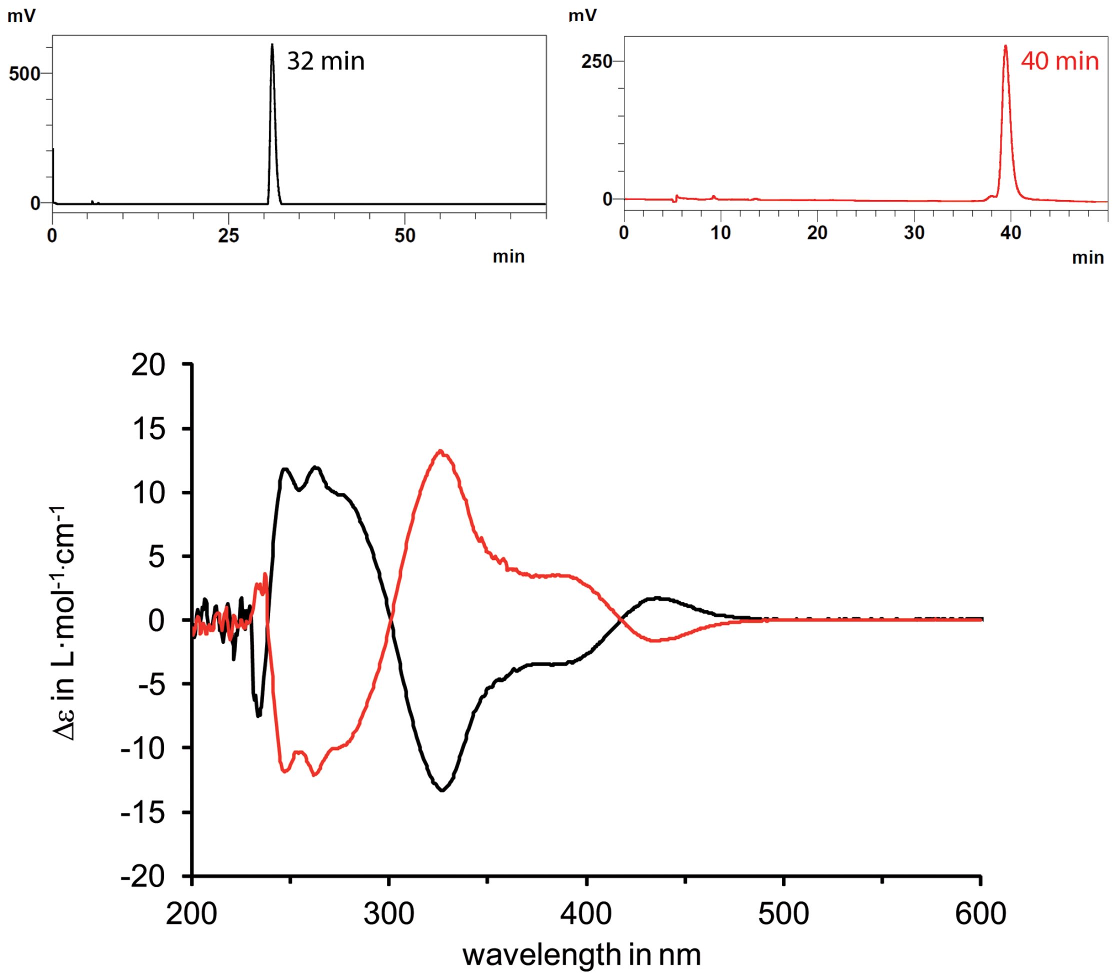

Supplementary Figure 15 | Molar CD spectra of the thermally stable *Z* isomers of motor 1. The spectra were measured in CH_2Cl_2 at 22 °C after chiral HPLC separation using a CHIRALPAK® IC column from Diacel (*n*-heptane : 2-PrOH = 8 : 2, at 40 °C) and assignment of the CD spectra to the retention times of the respective signals from HPLC separation. After measurement of CD spectra the solutions were subjected to chiral HPLC analysis again to assure isomeric purity. The thus obtained HPLC chromatograms are shown in the insets. *Z*-(*S*)-(*P*) isomer (black, belonging to the first fraction eluted after 32 min from the chiral HPLC separation) and *Z*-(*R*)-(*M*) isomer (red, belonging to the third fraction eluted after 40 min from the chiral HPLC separation). Crystal structural analysis confirmed the absolute *R* stereo configuration of the third fraction, which was also predicted by the theoretical CD calculations (see Supplementary Fig. 20).

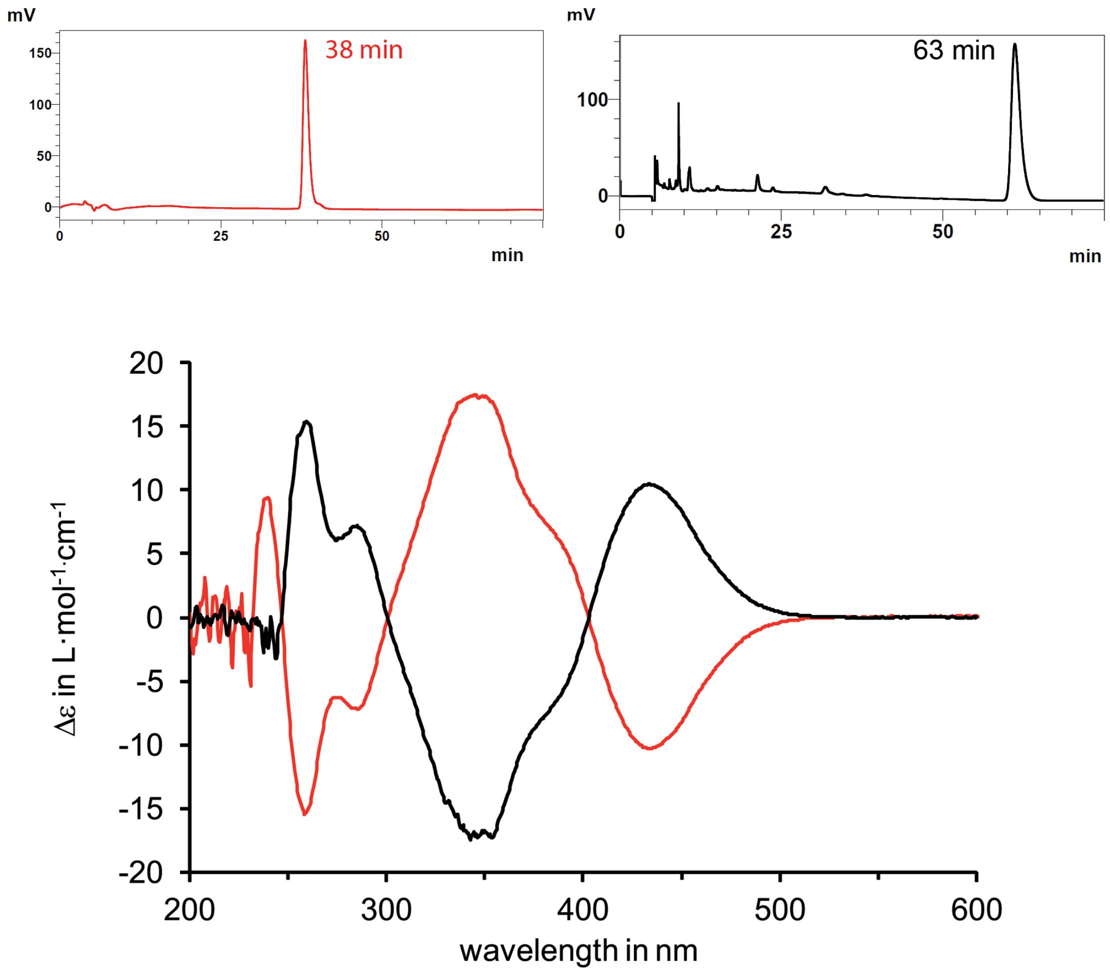

Supplementary Figure 16 | Molar CD spectra of the thermally stable *E* isomers of motor 1. The spectra were measured in CH_2Cl_2 at 22 °C after chiral HPLC separation using a CHIRALPAK® IC column from Diacel (*n*-heptane : 2-PrOH = 8 : 2, at 40 °C) and assignment of the CD spectra to the retention times of the respective signals from HPLC separation. After measurement of CD spectra the solutions were subjected to chiral HPLC analysis again to assure isomeric purity. The thus obtained HPLC chromatograms are shown in the insets. *E*-(*S*)-(*P*) isomer (black, belonging to the fourth fraction eluted after 63 min from the chiral HPLC separation) and *E*-(*R*)-(*M*) isomer (red, belonging to the second fraction eluted after 38 min from the chiral HPLC separation).

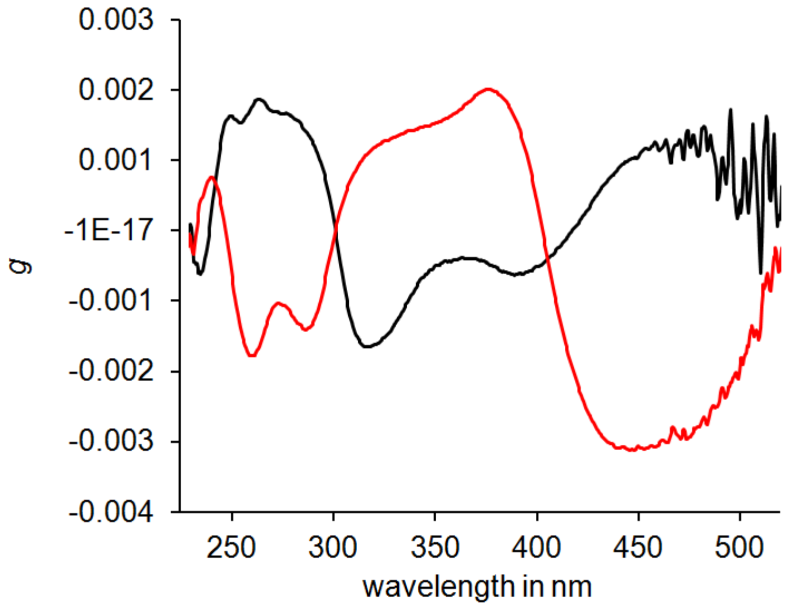

Supplementary Figure 17 | Measured *g* factors of motor 1. Only *g* factors of the *Z*-(*S*)-(*P*) isomer (black, belonging to the first fraction eluted after 32 min from the chiral HPLC separation) and *E*-(*R*)-(*M*) isomer (red, belonging to the second fraction eluted after 38 min from the chiral HPLC separation) are given. The *g* factors of the *Z*-(*R*)-(*M*) and *E*-(*S*)-(*P*) isomers are mirror symmetric and not shown for clarity reasons. The *g* factors were determined from the corresponding molar CD spectra and extinction coefficients in CH_2Cl_2 at 22 °C.

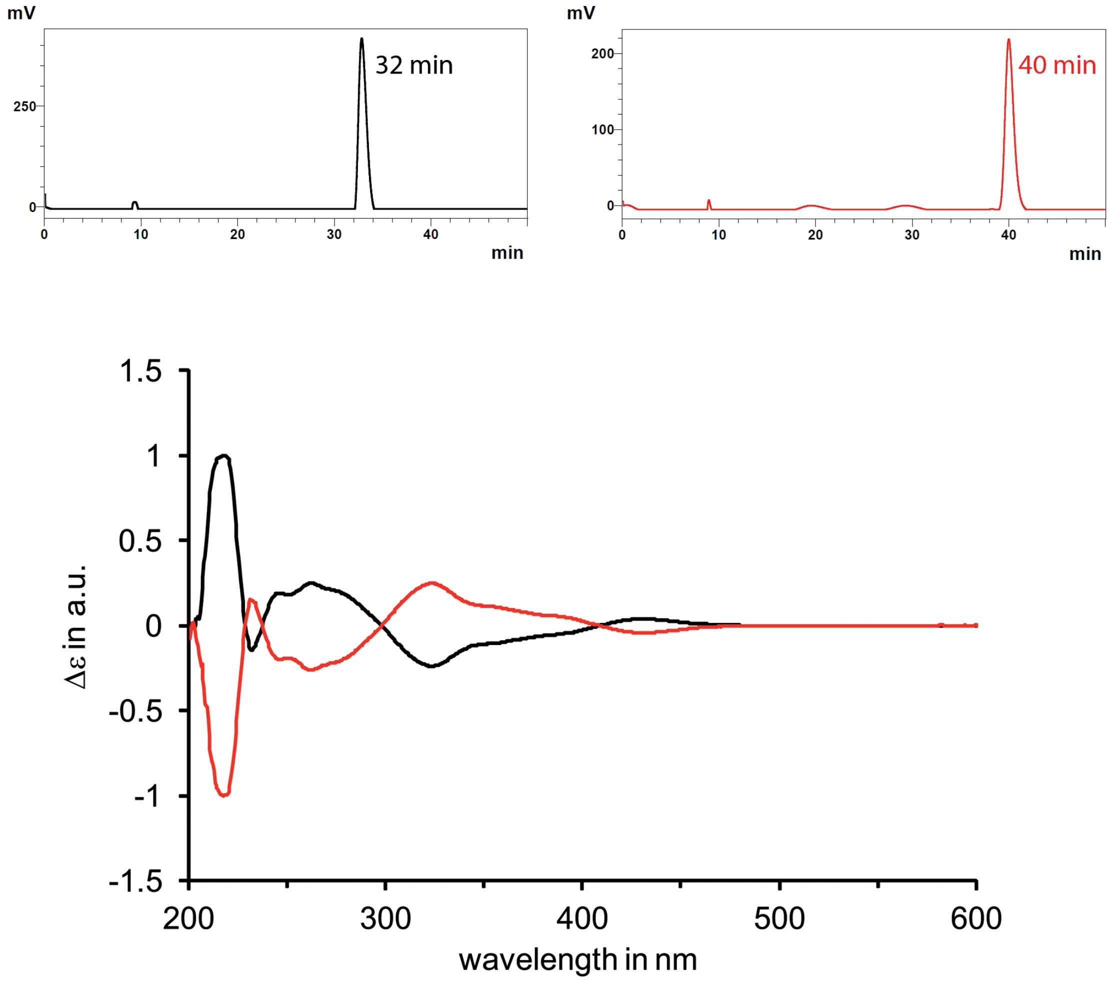

Supplementary Figure 18 | Normalized CD spectra of the thermally stable *Z* isomers of motor 1. The spectra were measured in a 8 : 2 mixture of *n*-heptane : 2-PrOH at 22 °C after chiral HPLC separation using a CHIRALPAK® IC column from Diacel (*n*-heptane : 2-PrOH = 8 : 2, at 40 °C) and assignment of the CD spectra to the retention times of the respective signals from HPLC separation. After measurement of CD spectra the solutions were subjected to chiral HPLC analysis again to assure isomeric purity. The thus obtained HPLC chromatograms are shown in the insets. *Z*-(*S*)-(*P*) isomer (black, belonging to the first fraction eluted after 32 min from the chiral HPLC separation) and *Z*-(*R*)-(*M*) isomer (red, belonging to the third fraction eluted after 40 min from the chiral HPLC separation).

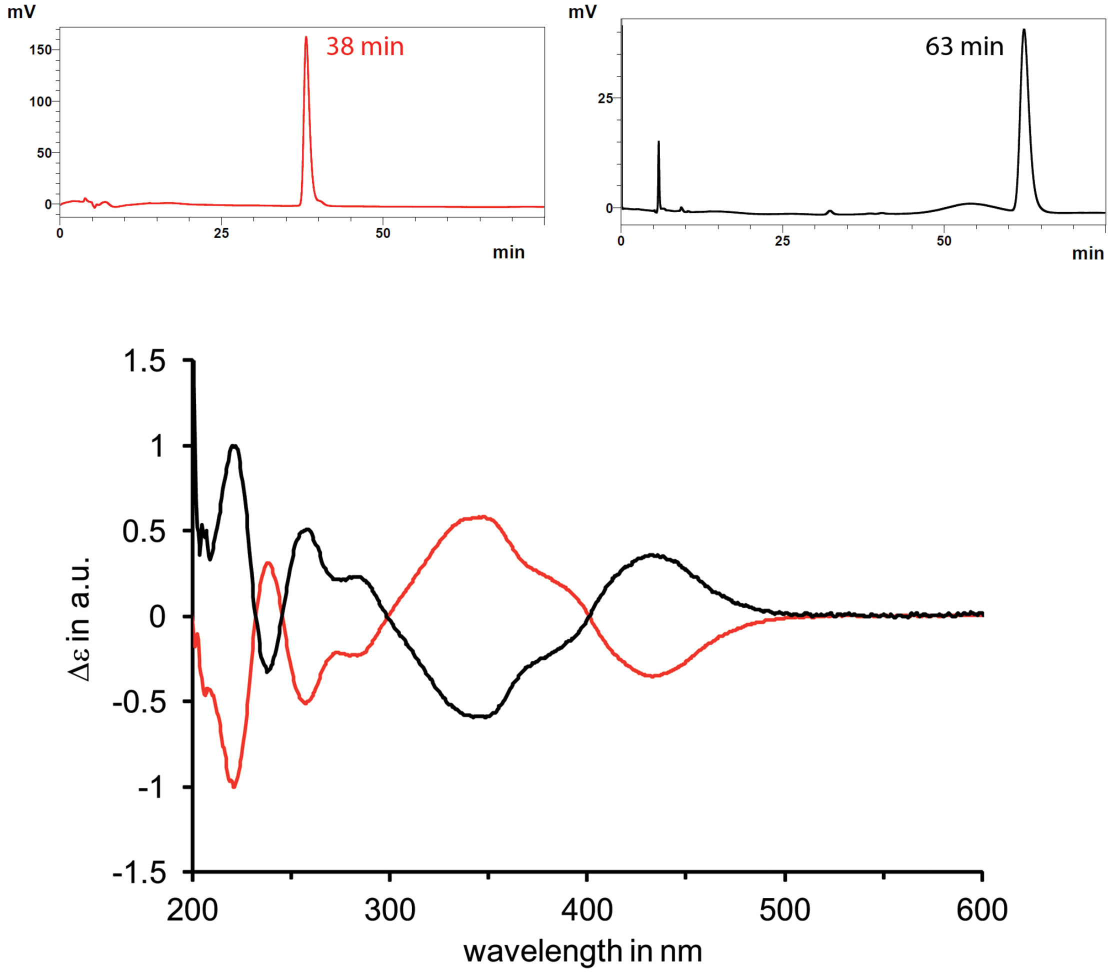

Supplementary Figure 19 | Normalized CD spectra of the thermally stable *E* isomers of motor 1. The spectra were measured in a 8 : 2 mixture of *n*-heptane : 2-PrOH at 22 °C after chiral HPLC separation using a CHIRALPAK® IC column from Diacel (*n*-heptane : 2-PrOH = 8 : 2, at 40 °C) and assignment of the CD spectra to the retention times of the respective signals from HPLC separation. After measurement of CD spectra the solutions were subjected to chiral HPLC analysis again to assure isomeric purity. The thus obtained HPLC chromatograms are shown in the insets. *E*-(*S*)-(*P*) isomer (black, belonging to the fourth fraction eluted after 63 min from the chiral HPLC separation) and *E*-(*R*)-(*M*) isomer (red, belonging to the second fraction eluted after 38 min from the chiral HPLC separation).

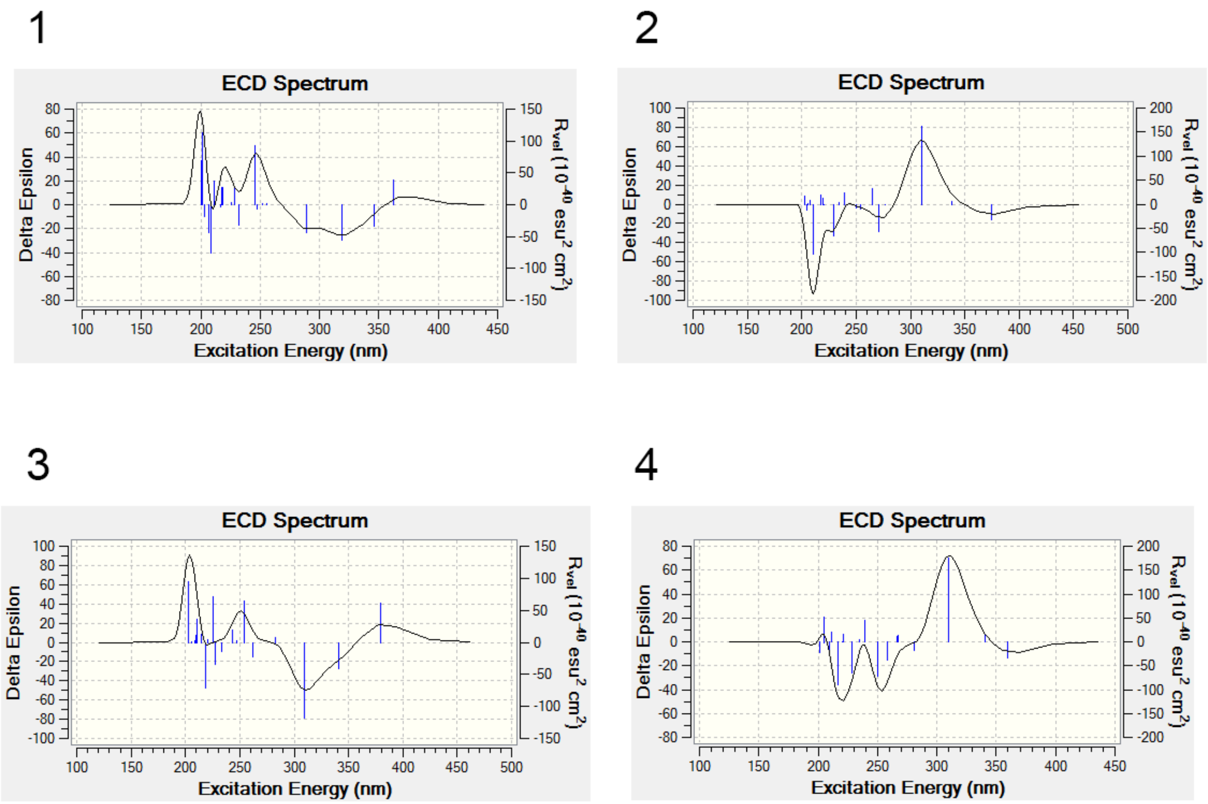

Supplementary Figure 20 | Theoretical CD spectra of motor 1 in CH_2Cl_2 calculated at the MPW1K level of theory using the 6-311++G(d,p) basis set. The spectra are blueshifted (ca. 50 nm) compared to the experimentally obtained CD spectra. 1: *Z*-(*S*)-(*P*) isomer of motor **1**. 2: *E*-(*S*)-(*M*) isomer of motor **1**. 3: *E*-(*S*)-(*P*) isomer of motor **1**. 4: *Z*-(*S*)-(*M*) isomer of motor **1**.

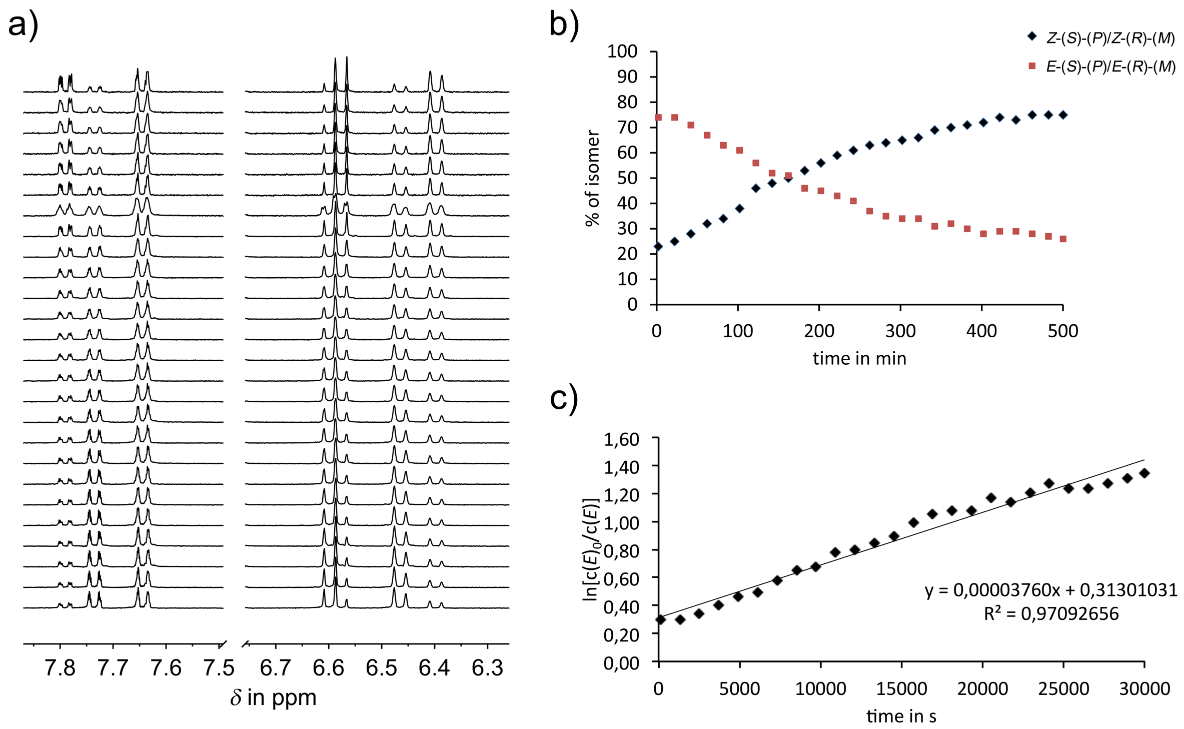

Supplementary Figure 21 | At elevated temperatures the *E*-(*S*)-(*P*)/*E*-(*R*)-(*M*) isomers of molecular motor 1 are interconverted into the *Z*-(*S*)-(*P*)/*Z*-(*R*)-(*M*) isomers. a) ^{1}H NMR spectra acquired at 100 °C (toluene-d_8, 400 MHz) during thermal conversion of *E*-(*S*)-(*P*)/*E*-(*R*)-(*M*) isomers to the *Z*-(*S*)-(*P*)/*Z*-(*R*)-(*M*) isomers in the dark. Spectra were taken in 20 min time intervals over the course of 8.3 h. Signals of the indicative aromatic signals are shown, which were integrated for kinetic analysis. b) ^{1}H NMR analysis of the kinetics of thermal *E* to *Z* isomerization of a 9.5 mM solution of motor **1** in toluene-d_8 at 100 °C. Starting point is a mixture of *Z*-(*S*)-(*P*)/*Z*-(*R*)-(*M*) and *E*-(*S*)-(*P*)/*E*-(*R*)-(*M*) isomers in a ratio of 25 : 75, respectively. This ratio is reversed after heating the solution to 100 °C for 8.3 h. c) First order kinetic analysis of the ^{1}H NMR data. The slope of the linear fit (formula given in the diagram) is the first-order rate constant $k_{(\text{therm.}E\rightarrow Z)}$ = 0.000038 s^{-1}.

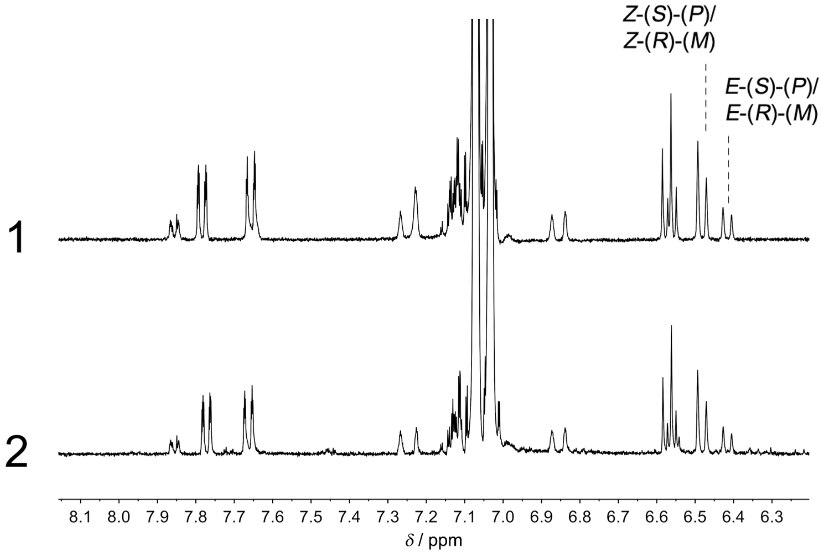

Supplementary Figure 22 | ^{1}H NMR analysis (400 MHz, 27 °C) of the equilibrium distribution of *Z*-(*S*)-(*P*)/*Z*-(*R*)-(*M*) and *E*-(*S*)-(*P*)/*E*-(*R*)-(*M*) isomers. A 5.4 mM solution of motor **1** in *o*-xylene-d_{10} was heated to 130 °C for 12 h. Signals of the aromatic region used for integration are shown. 73% of the *Z*-(*S*)-(*P*)/*Z*-(*R*)-(*M*) isomer and 27% of the *E*-(*S*)-(*P*)/*E*-(*R*)-(*M*) isomer are found consistently. 1: ^{1}H NMR spectrum recorded after heating a solution of pure *Z*-(*S*)-(*P*)/*Z*-(*R*)-(*M*) isomer to 130 °C for 23 h followed by shock-freezing to 0 °C. 2: ^{1}H NMR spectrum recorded after heating a solution of 55% *Z*-(*S*)-(*P*)/*Z*-(*R*)-(*M*) and 45% *E*-(*S*)-(*P*)/*E*-(*R*)-(*M*) isomer to 130 °C for 23 h followed by shock-freezing to 0 °C.

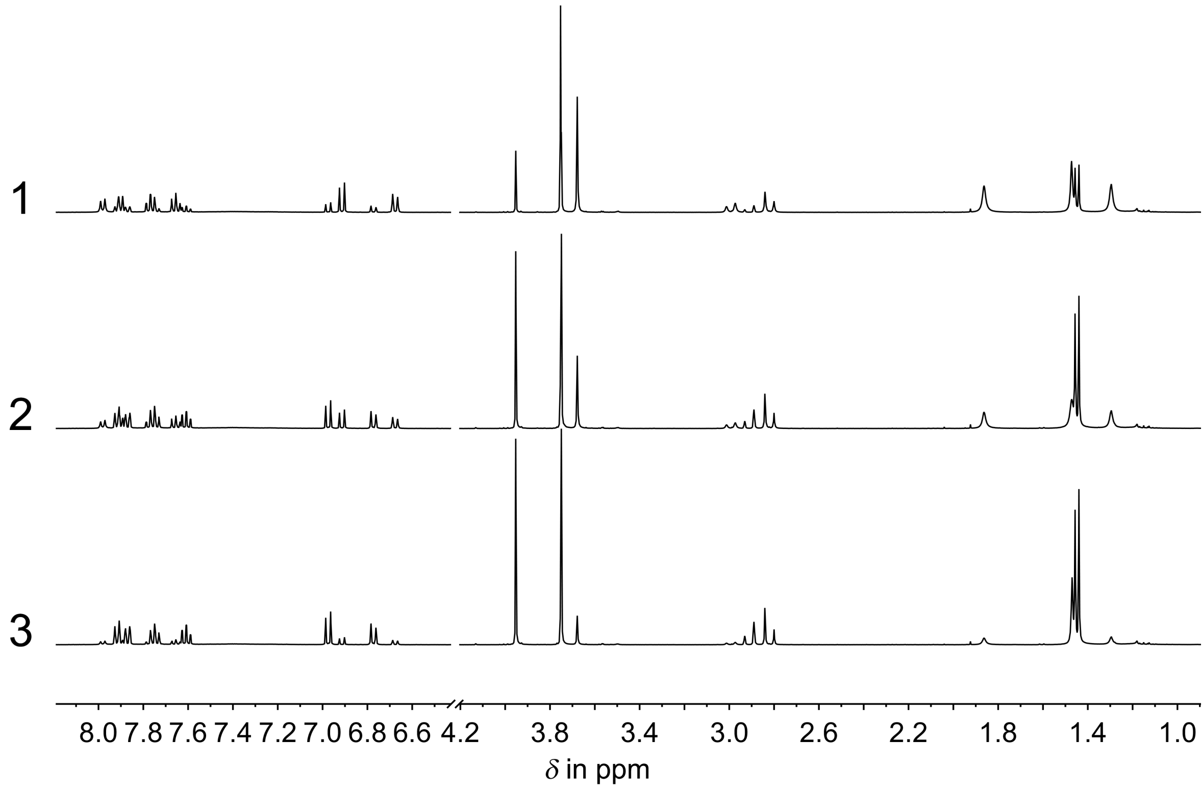

Supplementary Figure 23 | ^{1}H NMR spectra (CD_2Cl_2, 400 MHz, 27 °C) of motor 1 showing the isomer composition after irradiation at 23 °C. 1: PSS after irradiation with 405 nm light. The isomer composition is *Z*-(*S*)-(*P*)/*Z*-(*R*)-(*M*) : *E*-(*S*)-(*P*)/*E*-(*R*)-(*M*) = 22 : 78. 2: PSS after irradiation with 460 nm light. The isomer composition is *Z*-(*S*)-(*P*)/*Z*-(*R*)-(*M*) : *E*-(*S*)-(*P*)/*E*-(*R*)-(*M*) = 58 : 42. 3: PSS after irradiation with 490 nm light. The isomer composition is *Z*-(*S*)-(*P*)/*Z*-(*R*)-(*M*) : *E*-(*S*)-(*P*)/*E*-(*R*)-(*M*) = 83 : 17.

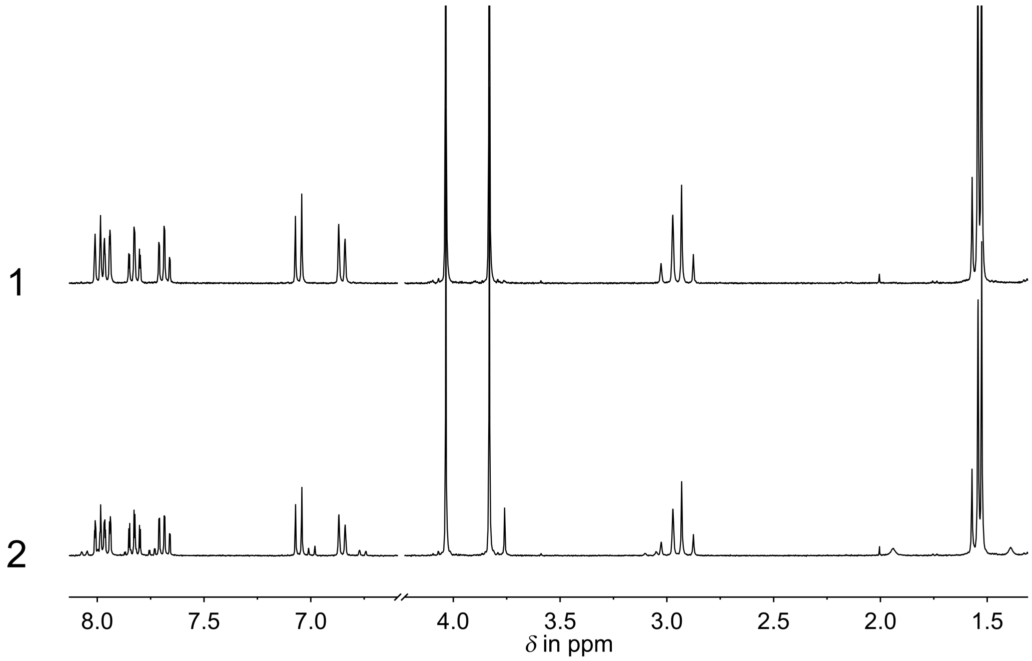

Supplementary Figure 24 | ^{1}H NMR spectra (CD_2Cl_2, 400 MHz) of the *Z*-(*S*)-(*P*)/*Z*-(*R*)-(*M*) isomers of motor 1 at a concentration of 31 mM. 1: Before irradiation. 2: After irradiation with 505 nm light. The *E*-(*S*)-(*P*)/*E*-(*R*)-(*M*) isomers are formed in 13% yield.

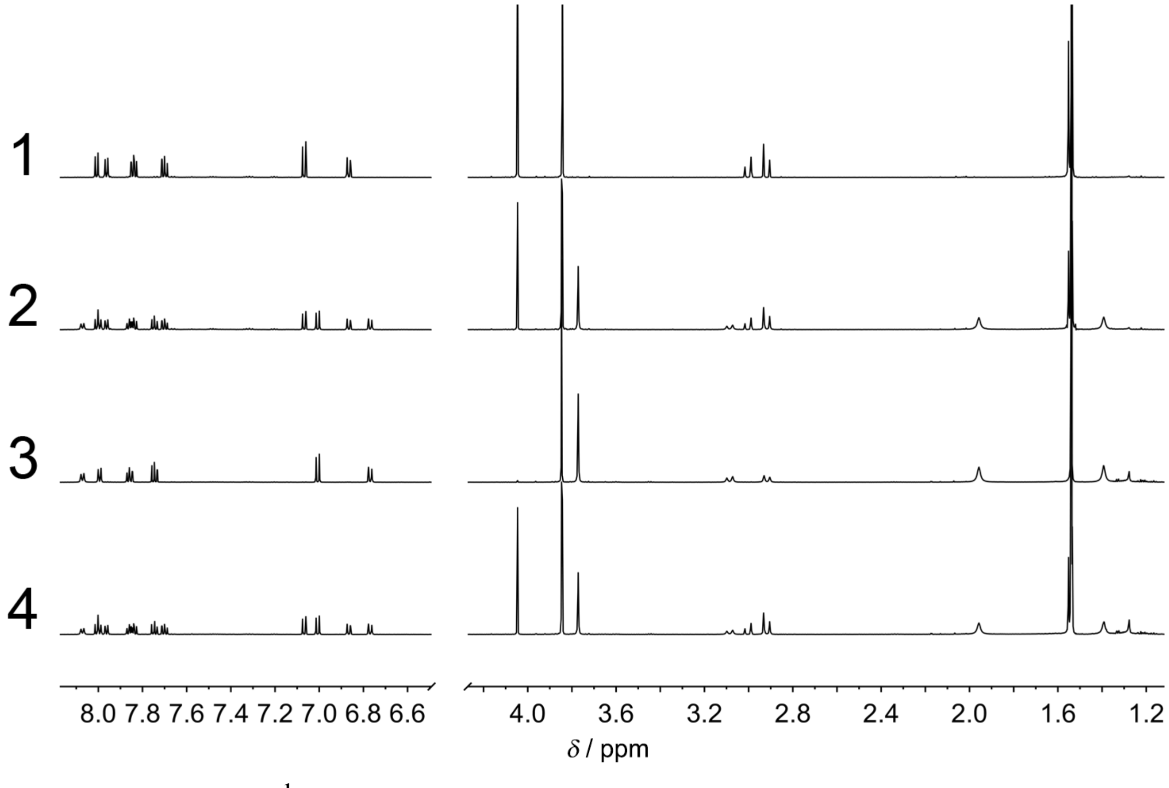

Supplementary Figure 25 | ^{1}H NMR spectra (CD_2Cl_2, 600 MHz) before and after sunlight irradiation of motor 1. The same photostationary state is reached starting from different pure isomers. 1: Pure *Z*-(*S*)-(*P*)/*Z*-(*R*)-(*M*) isomers before irradiation at a concentration of 2.5 mM. 2: After 20 min sunlight irradiation. The signals of the *E*-(*S*)-(*P*)/*E*-(*R*)-(*M*) isomers are present in 53%. 3: Pure *E*-(*S*)-(*P*)/*E*-(*R*)-(*M*) isomers before irradiation at a concentration of 2.7 mM. 4: After 20 min sunlight irradiation. The signals of the *Z*-(*S*)-(*P*)/*Z*-(*R*)-(*M*) isomers are present in 47%.

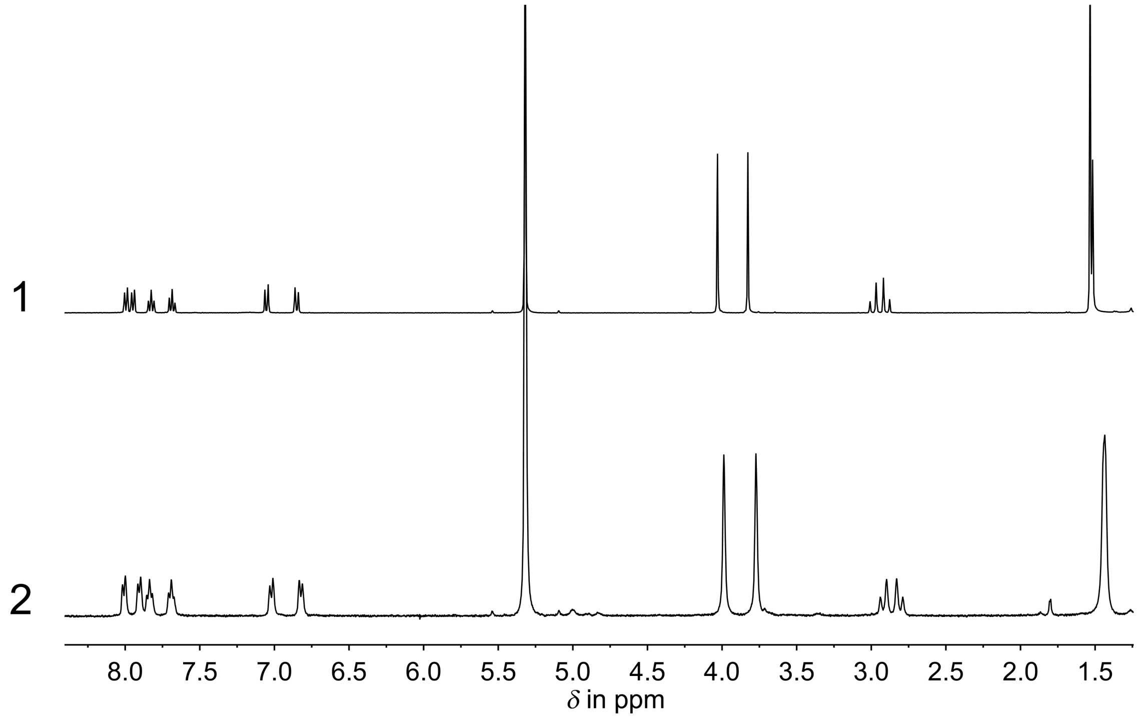

Supplementary Figure 26 | ^{1}H NMR spectra of the *Z*-(*S*)-(*P*)/*Z*-(*R*)-(*M*) isomer of motor 1 (CD_2Cl_2, 400 MHz). 1: Acquired at 22 °C. 2: Acquired at –90 °C. Only small temperature effects on chemical shifts of the signals are seen.

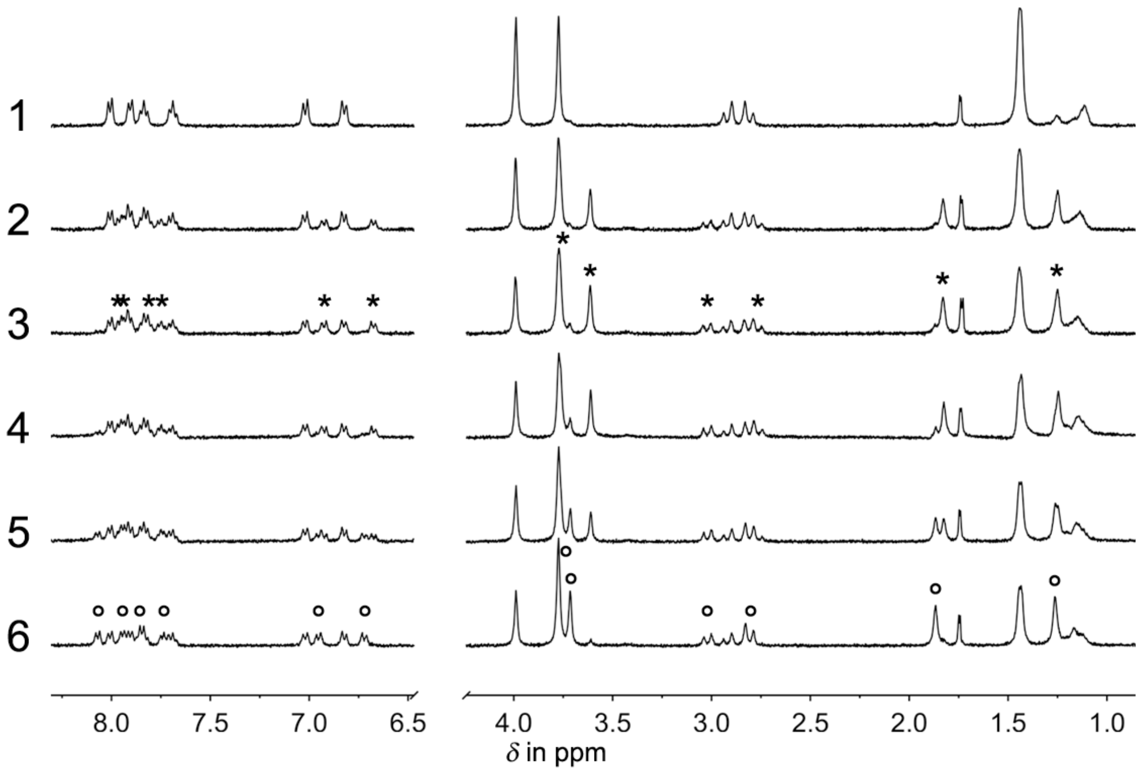

Supplementary Figure 27 | ¹H NMR spectra acquired at –90 °C (CD_2Cl_2, 400 MHz). Spectra acquired during high power 460 nm irradiation of a 1.25 mM solution of the *Z*-(*S*)-(*P*)/*Z*-(*R*)-(*M*) isomer of motor **1** (spectra 1-3) and during thermal isomerization of the intermediate *E*-(*S*)-(*M*)/*E*-(*R*)-(*P*) isomer (spectra 4-6) are shown. Signals of the *E*-(*S*)-(*M*)/*E*-(*R*)-(*P*) isomer are indicated by * and signals of the *E*-(*S*)-(*P*)/*E*-(*R*)-(*M*) isomer are indicated by °. All spectra are normalized to the intensity of the residual solvent signal. 1: ¹H NMR spectrum of *Z*-(*S*)-(*P*)/*Z*-(*R*)-(*M*) isomer before irradiation. 2: After 2 min of 460 nm irradiation. 3: After 4 min of 460 nm irradiation the PSS is reached. 4: After reaching the PSS the light is turned off for 2 min. 5: Light turned off after reaching the PSS for 10 min. 6: Light turned off after reaching the PSS for 60 min.

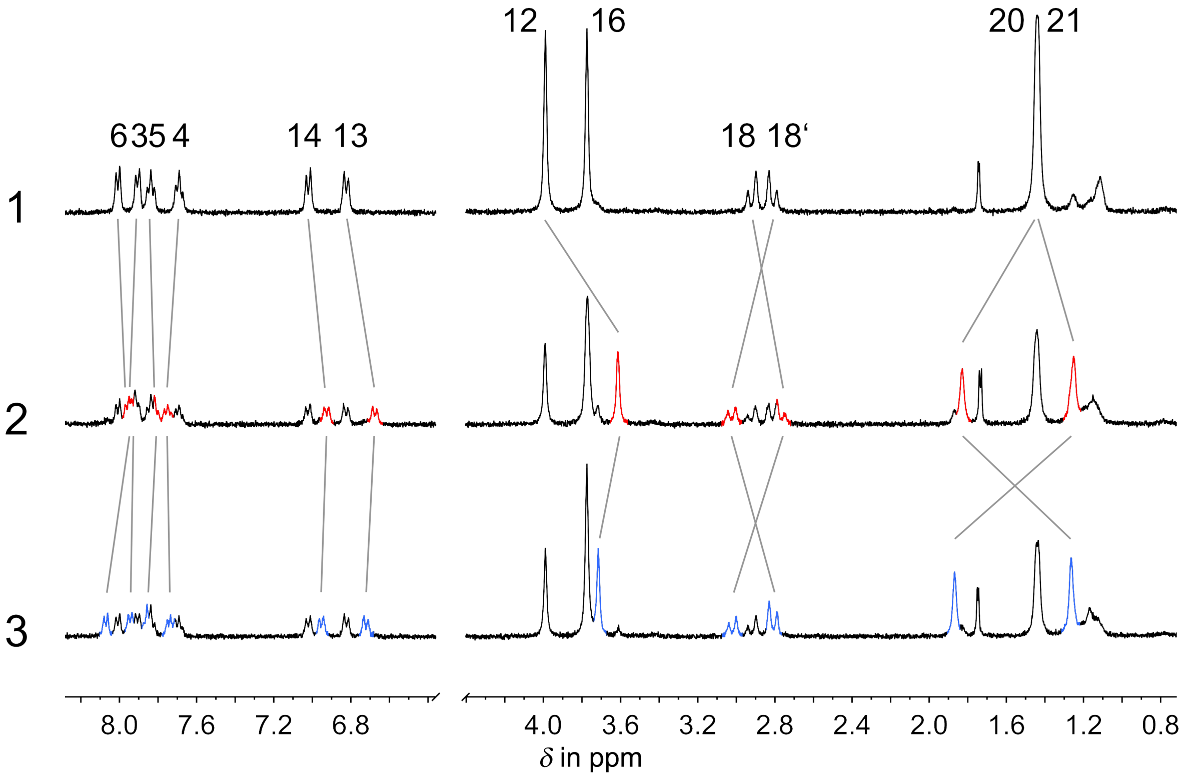

Supplementary Figure 28 | Selected ^{1}H NMR spectra of motor 1 acquired at –90 °C (CD_2Cl_2, 400 MHz) at a concentration of 1.25 mM. Grey lines indicate individual changes of signals in the spectra inferred from the calculated spectra. All spectra are normalized to the intensity of the residual solvent signal. 1: *Z*-(*S*)-(*P*)/*Z*-(*R*)-(*M*) isomer of motor **1**. 2: PSS after irradiation with high power 460 nm (new signals of an intermediate isomer are shown in red). 3: After keeping the irradiated sample in the dark for 60 min the new signals observed in the PSS are completely converted into signals of the *E*-(*S*)-(*P*)/*E*-(*R*)-(*M*) isomer (blue signals). The signals of the *Z*-(*S*)-(*P*)/*Z*-(*R*)-(*M*) isomer do not change during this thermal conversion. The chemical shifts of the red signals are more similar to the signals of the *E*-(*S*)-(*P*)/*E*-(*R*)-(*M*) isomer (blue signals) than to the signals of the *Z*-(*S*)-(*P*)/*Z*-(*R*)-(*M*) isomer. The red signals are assigned to the *E*-(*S*)-(*M*)/*E*-(*R*)-(*P*) isomer of motor **1**.

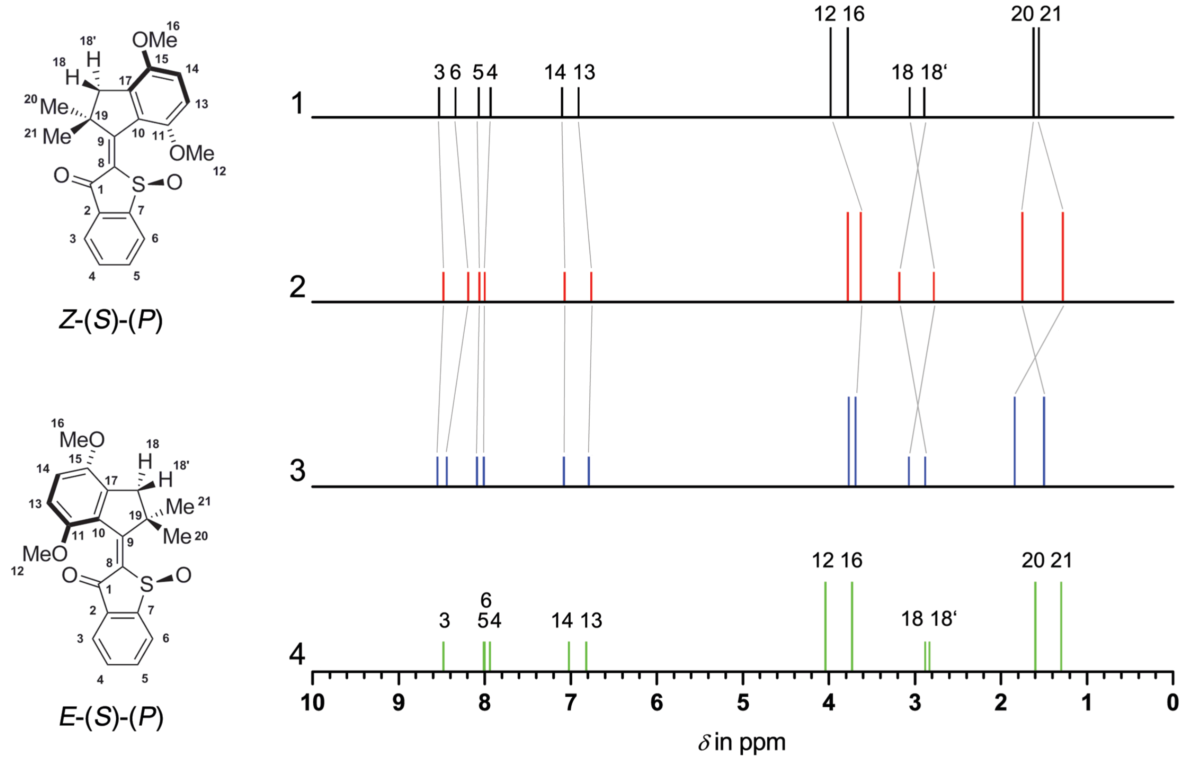

Supplementary Figure 29 | Theoretical ^{1}H NMR spectra of motor 1 calculated at the MPW1K level of theory using the 6-31+G(d,p) basis set and the structures of the *Z*-(*S*)-(*P*) and *E*-(*S*)-(*P*) isomers for assignment of the signals to the structure. Grey lines indicate individual changes of signals in the spectra. 1: *Z*-(*S*)-(*P*) isomer of motor **1**. 2: *E*-(*S*)-(*M*) isomer of motor **1**. 3: *E*-(*S*)-(*P*) isomer of motor **1**. 4: *Z*-(*S*)-(*M*) isomer of motor **1**, which could not be observed experimentally. The numerical values of all calculated spectra are compared to the experimental values in Supplementary Table 1.

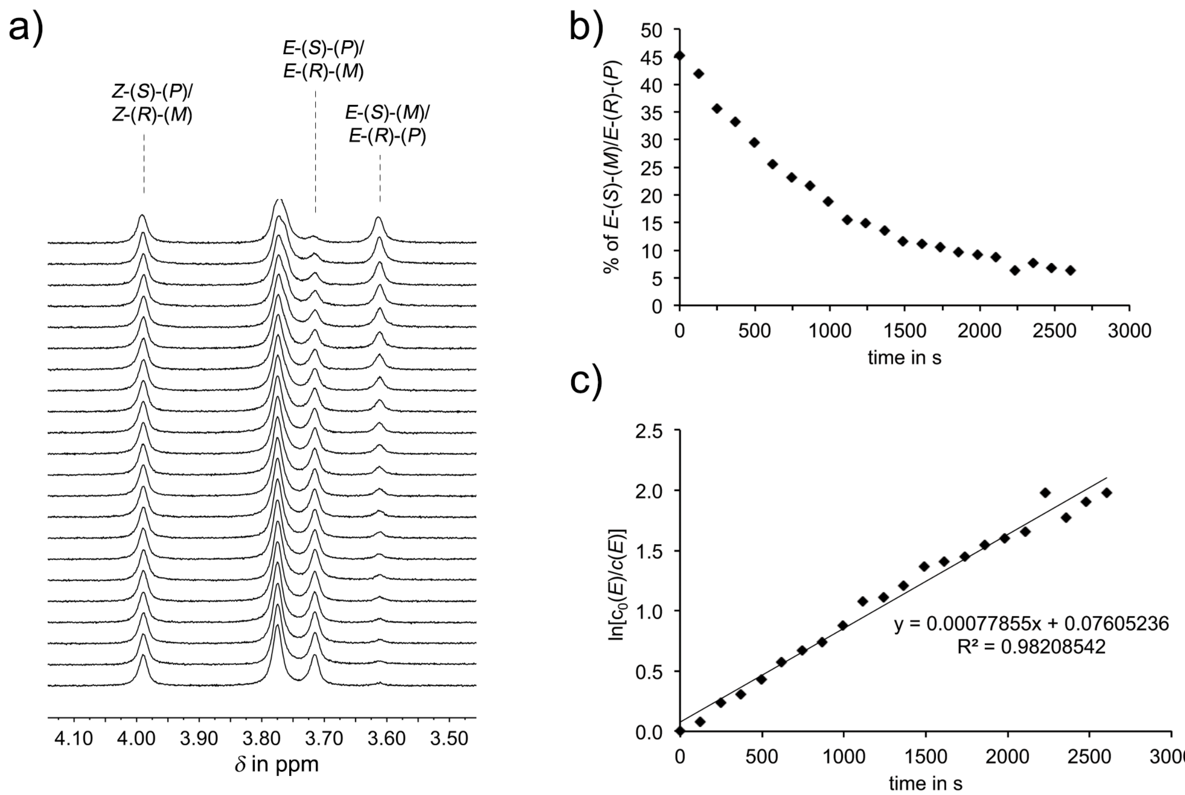

Supplementary Figure 30 | Kinetic analysis of the thermal conversion of the *E*-(*S*)-(*M*)/*E*-(*R*)-(*P*) isomers to the *E*-(*S*)-(*P*)/*E*-(*R*)-(*M*) isomers. a) ^{1}H NMR spectra acquired at –90 °C (CD_2Cl_2, 400 MHz) during thermal conversion of the photo generated *E*-(*S*)-(*M*)/*E*-(*R*)-(*P*) isomers to the *E*-(*S*)-(*P*)/*E*-(*R*)-(*M*) isomers in the dark. Spectra were taken in 2.04 min time intervals over the course of 45 min. Signals of the indicative methoxy groups are shown, which were integrated for kinetic analysis. b) Decrease of *E*-(*S*)-(*M*)/*E*-(*R*)-(*P*) isomers with time. c) First order kinetic analysis of the ^{1}H NMR data. The slope of the linear fit (formula given in the diagram) is the first order rate constant $k_{(\text{therm. } ESM\rightarrow ESP)}$ = 0.00078 s^{-1}.

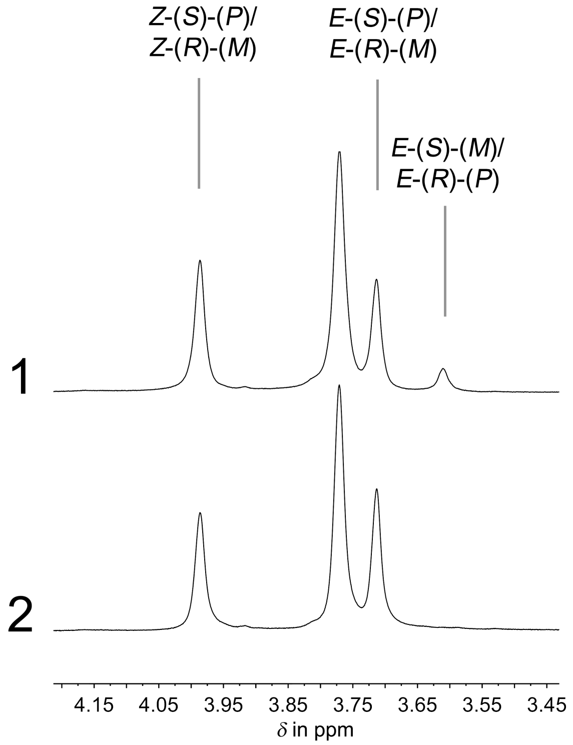

Supplementary Figure 31 | ^{1}H NMR spectra acquired at –90 °C (CD_2Cl_2, 400 MHz). Signals of the methoxy groups are shown. 1: Before thermal conversion of the photo generated *E*-(*S*)-(*M*)/*E*-(*R*)-(*P*) isomers. 2: After warming the solution to –70 °C in the dark for 5 min followed by cooling to –90 °C. No residual signals of the *E*-(*S*)-(*M*)/*E*-(*R*)-(*P*) isomers are seen after the annealing step.

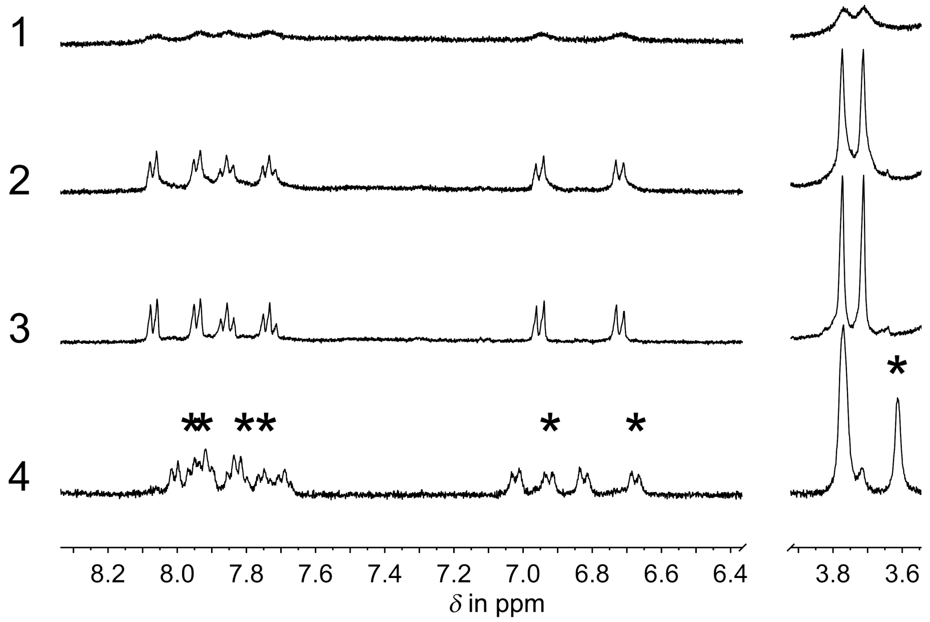

Supplementary Figure 32 | ^{1}H NMR spectra (CD_2Cl_2, 400 MHz, –90 °C) of the *E*-(*S*)-(*P*)/*E*-(*R*)-(*M*) isomers of motor 1 at a concentration of 5.8 mM after the sample was shock frozen in liquid N_2 starting from 22 °C. No signals of the *E*-(*S*)-(*M*)/*E*-(*R*)-(*P*) isomers were observed after thawing. The aromatic part of the spectra and signals of the methoxy groups are shown. 1: First ^{1}H NMR spectrum recorded 165 s after insertion into the NMR spectrometer, which was precooled to –90 °C. The sample is not thawed yet. 2: ^{1}H NMR spectrum recorded 5.00 min after insertion into the NMR spectrometer. Thawing is almost complete. 3: ^{1}H NMR spectrum recorded 7.00 min after insertion into the NMR spectrometer. 4: ^{1}H NMR spectrum showing the signals of the *E*-(*S*)-(*M*)/*E*-(*R*)-(*P*) isomers (*) obtained after irradiation at –90 °C for comparison. The residual signals belong to the *Z*-(*S*)-(*P*)/*Z*-(*R*)-(*M*) isomers.

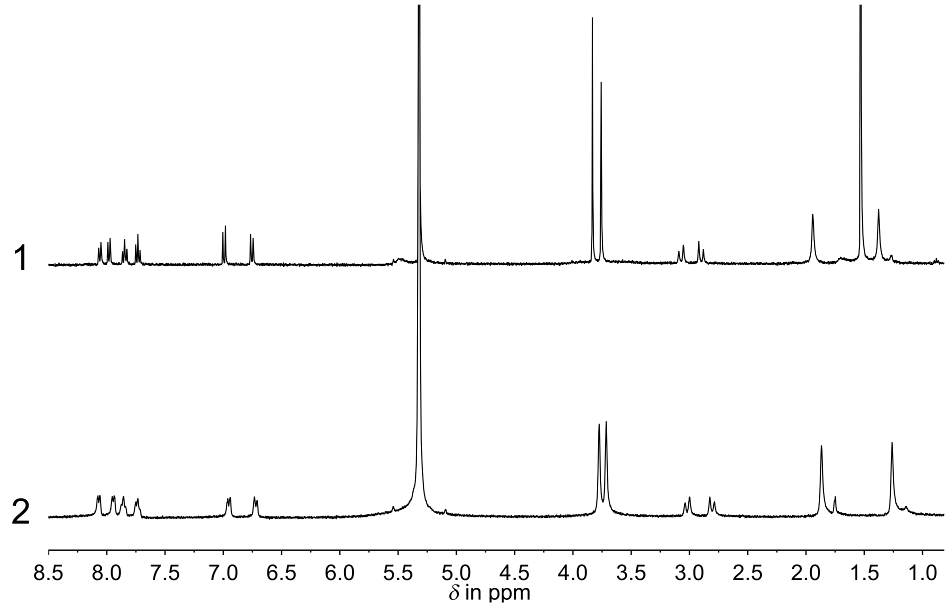

Supplementary Figure 33 | ^{1}H NMR spectra of the *E*-(*S*)-(*P*)/*E*-(*R*)-(*M*) isomer of motor 1 (CD_2Cl_2, 400 MHz). 1: Acquired at 22 °C. 2: Acquired at –90 °C. Only small temperature effects on chemical shifts of the signals are seen.

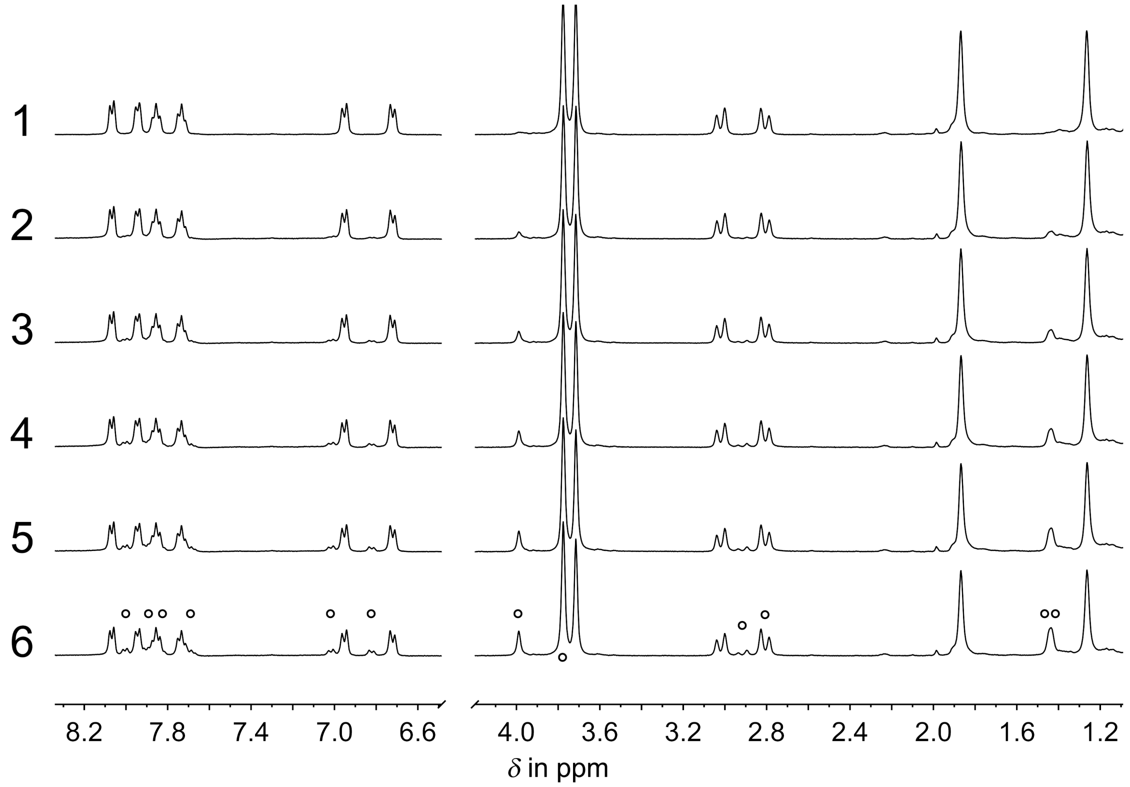

Supplementary Figure 34 | ^{1}H NMR spectra acquired at –90 °C (CD_2Cl_2, 400 MHz). Spectra were recorded during low power 460 nm irradiation of a 2.5 mM solution of *E*-(*S*)-(*P*)/*E*-(*R*)-(*M*)-**1** at time intervals of 2.04 min (spectrum 1: starting point). Signals of the *Z*-(*S*)-(*P*)/*Z*-(*R*)-(*M*) isomers are indicated by °. All spectra are normalized to the intensity of the residual solvent signal. Only the buildup of the *Z*-(*S*)-(*P*)/*Z*-(*R*)-(*M*) isomers is seen over time.

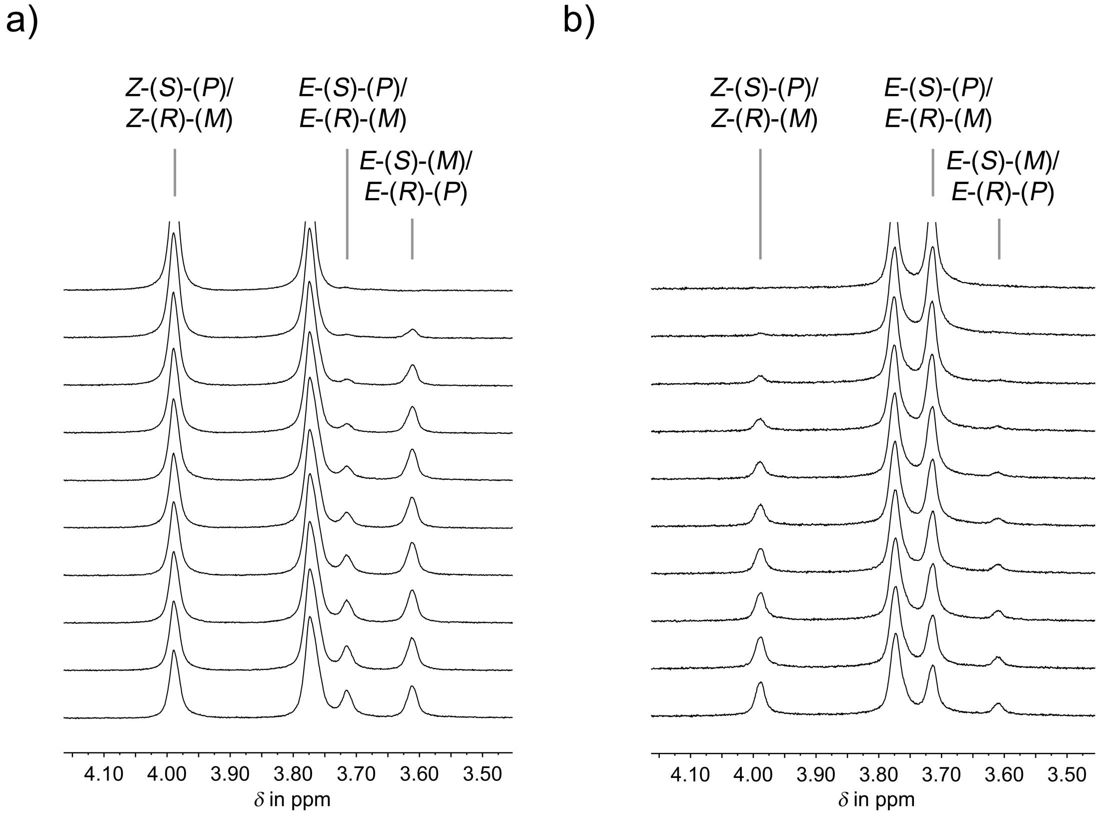

Supplementary Figure 35 | ^{1}H NMR spectra acquired at –90 °C (CD_2Cl_2, 400 MHz) during medium power 460 nm irradiation of motor 1 at time intervals of 2.04 min. Signals of the methoxy groups are shown exemplarily. a) A 1.25 mM solution of *Z*-(*S*)-(*P*)/*Z*-(*R*)-(*M*) isomers was used as starting point (top spectrum). First the signals of the *E*-(*S*)-(*M*)/*E*-(*R*)-(*P*) isomers are observed and only later the signals of the *E*-(*S*)-(*P*)/*E*-(*R*)-(*M*) isomers are starting to build up. b) A 2.7 mM solution of the *E*-(*S*)-(*P*)/*E*-(*R*)-(*M*) isomers was used as starting point (top spectrum). First the signals of the *Z*-(*S*)-(*P*)/*Z*-(*R*)-(*M*) isomers are observed and only later the signals of the *E*-(*S*)-(*M*)/*E*-(*R*)-(*P*) isomers are starting to build up.

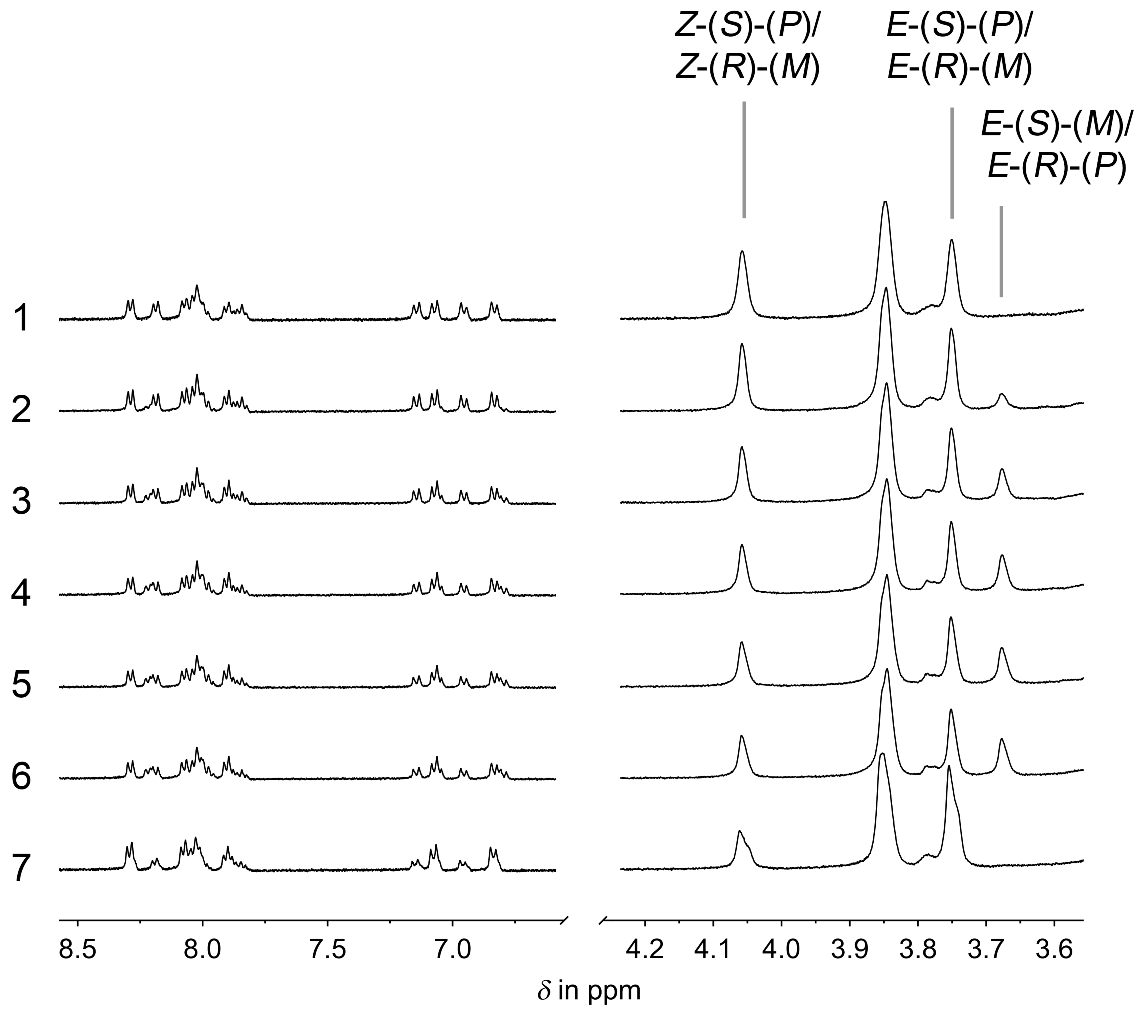

Supplementary Figure 36 | ^{1}H NMR spectra acquired at –100 °C (diethyl ether-d_{10}, 400 MHz) during 415 nm irradiation of motor 1 at time intervals of 2.04 min. Signals of the aromatic protons and methoxy groups are shown. Methoxy group signals indicative for the different isomeric species are indicated. 1: A 1 : 1 mixture of *Z*-(*S*)-(*P*)/*Z*-(*R*)-(*M*) and *E*-(*S*)-(*P*)/*E*-(*R*)-(*M*) isomers was used as starting point (top spectrum). 2-6: Upon irradiation signals of the *Z*-(*S*)-(*P*)/*Z*-(*R*)-(*M*) isomers are decreasing and signals of the *E*-(*S*)-(*M*)/*E*-(*R*)-(*P*) isomers are building up. No additional signals of a fourth species are seen. 7: After annealing - warming to –70 °C and subsequent cooling to –100 °C - the signals of the *E*-(*S*)-(*M*)/*E*-(*R*)-(*P*) isomers are converted exclusively into the signals of the *E*-(*S*)-(*P*)/*E*-(*R*)-(*M*) isomers.

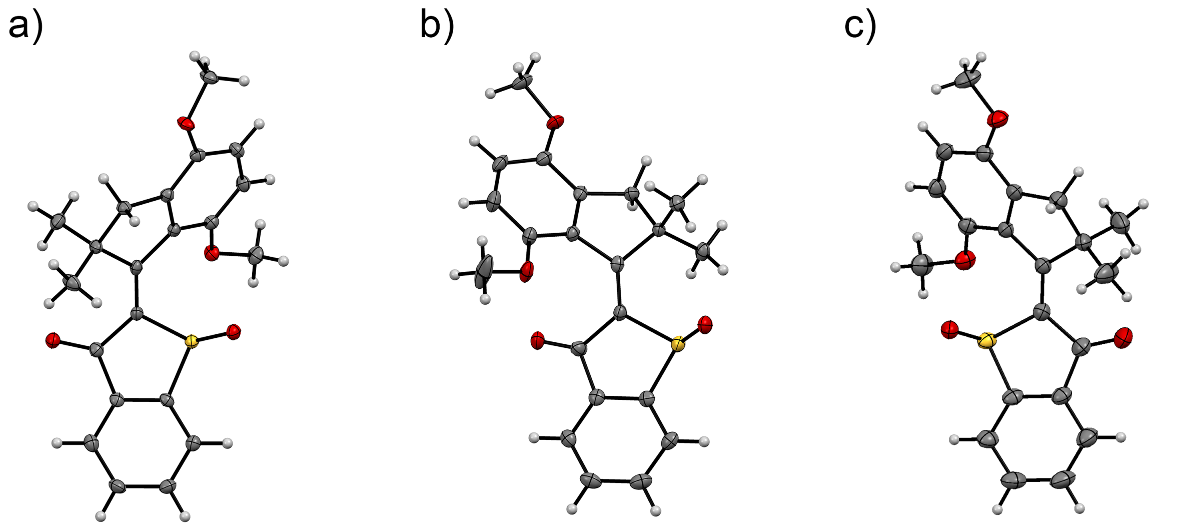

Z-(*S*)-(*P*)-**1** *E*-(*S*)-(*P*)-**1** *Z*-(*R*)-(*M*)-**1**

Supplementary Figure 37 | Crystal structures of motor 1 with probability ellipsoids set to 50%. a) Crystal structure of racemic *Z*-(*S*)-(*P*)/*Z*-(*R*)-(*M*) isomers, only the *Z*-(*S*)-(*P*) isomer is shown (CCDC 1061969). b) Crystal structure of racemic *E*-(*S*)-(*P*)/*E*-(*R*)-(*M*) isomers, only the *E*-(*S*)-(*P*) isomer is shown (CCDC 1061970). c) Crystal structure of enantiomerically pure *Z*-(*R*)-(*M*) isomer (CCDC 1406625), obtained after chiral HPLC separation of the enantiomers (retention time 38 min on a CHIRALPAK® IC column from Diacel using *n*-heptane : 2-PrOH = 8 : 2, at 40 °C).

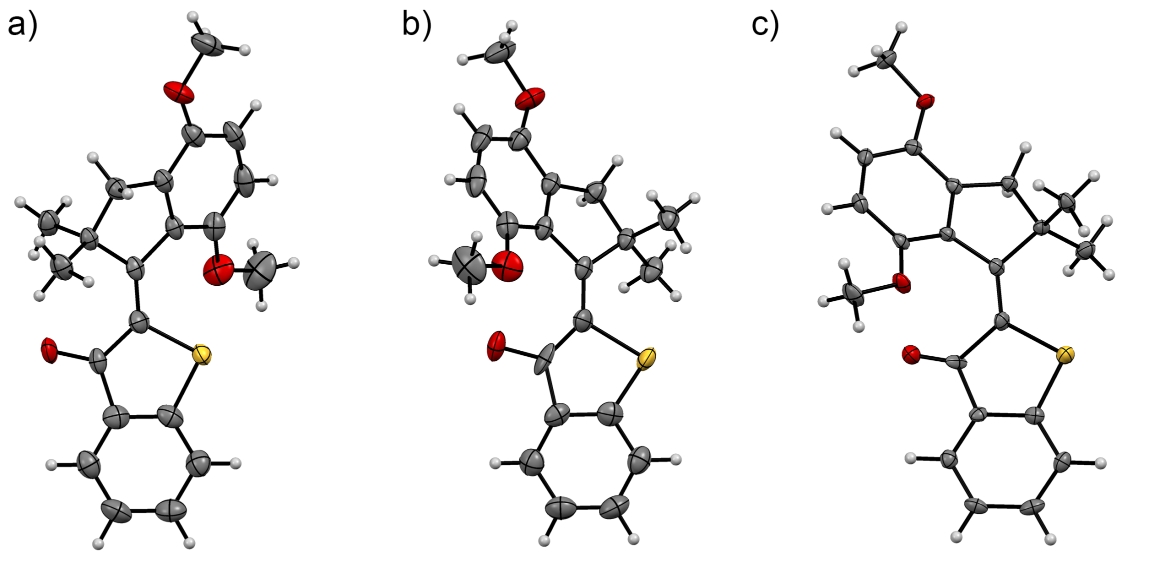

Z-(*P*) *E*-(*P*) *E*-(*P*)

Supplementary Figure 38 | Crystal structures of racemic precursor deoxy-motor 1 with probability ellipsoids set to 50%. Both *Z* and *E* isomeric forms are found in one crystal (CCDC 1408096), with 68% of the *Z* isomers and 32% of the *E* isomers. The pure *E* isomeric forms were crystallized in a second attempt (CCDC 1408097). a) Crystal structure of racemic *Z*-(*P*)/*Z*-(*M*) isomers, only the *Z*-(*P*) isomer is shown (CCDC 1408096). b) Crystal structure of racemic *E*-(*P*)/*E*-(*M*) isomers, only the *E*-(*P*) isomer is shown (CCDC 1408096). c) Crystal structure of racemic *E*-(*P*)/*E*-(*M*) isomers (CCDC 1408097).

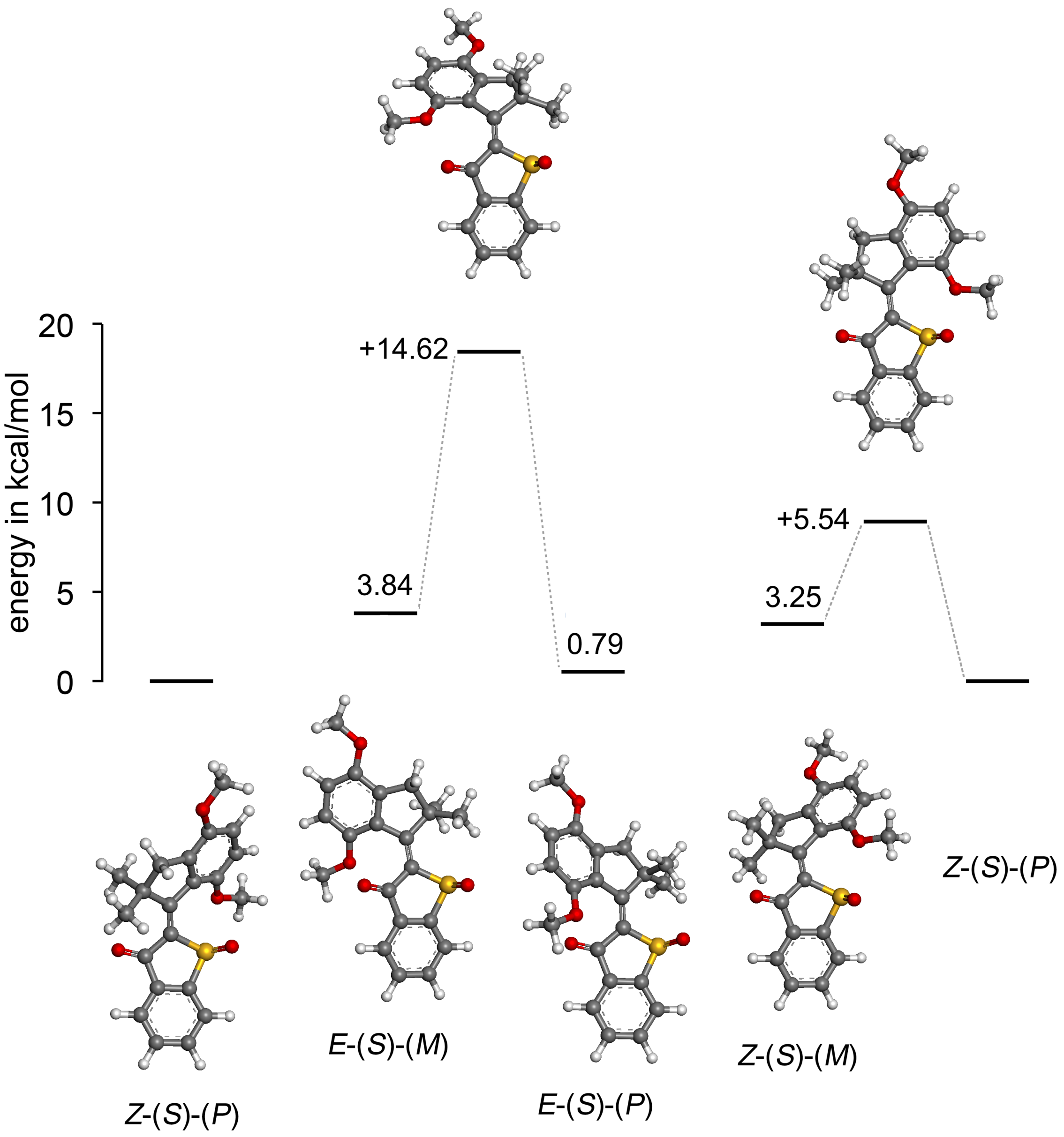

Supplementary Figure 39 | Ground state energy profile of motor 1 calculated at the DFT MPW1K level of theory using the 6-31+G(d,p) basis set. Energies are given in kcal/mol. The *S*-enantiomeric form of motor **1** has been used in the theoretical description. All optimized structures were confirmed to be stationary points by frequency analysis. Corresponding structures are given as ball and stick representations for every stationary point.

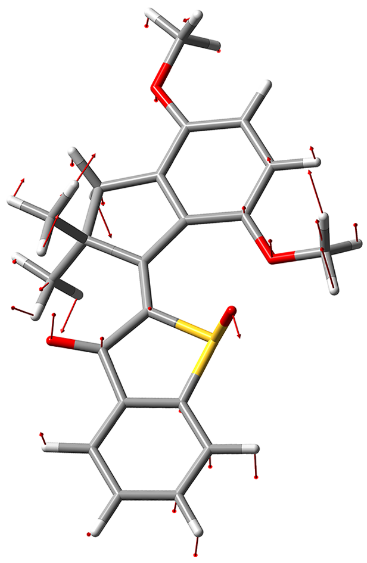

Supplementary Figure 40 | *Z*-(*S*)-(*M*)/*Z*-(*S*)-(*P*)-1 transition state geometry. DFT energy = –1511.01334787 Hartree; saddle point frequency = –34.2131 cm^{-1}. The structure is shown together with displacement vectors.

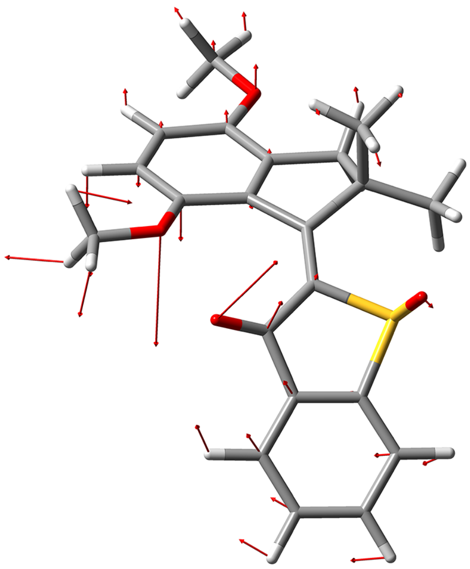

Supplementary Figure 41 | *E*-(*S*)-(*M*)/*E*-(*S*)-(*P*)-1 transition state geometry. DFT energy = –1510.99582657 Hartree; saddle point frequency = –68.1463 cm^{-1}. The structure is shown together with displacement vectors.

Proton	Z-(S)-(P)		E-(S)-(M)		E-(S)-(P)		Z-(S)-(M)
	exp.	calc.	exp.	calc.	exp.	calc.	calc.
**3**	7.91	8.53	7.93	8.48	7.94	8.55	8.48
**4**	7.69	7.93	7.75	8.00	7.74	8.01	7.94
**5**	7.84	8.07	7.82	8.06	7.86	8.09	8.01
**6**	8.01	8.34	7.96	8.19	8.07	8.44	8,00
**12**	3.99	3.98	3.61	3.63	3.72	3.69	4.04
**13**	6.82	6.91	6.68	6.76	6.72	6.79	6.82
**14**	7.02	7.10	6.92	7.07	6.95	7.08	7.02
**16**	3.77	3.78	3.77	3.78	3.77	3.77	3.73
**18**	2.92	3.06	3.023	3.18	3.018	3.07	2.83
**18'**	2.81	2.89	2.77	2.78	2.81	2.88	2.88
**20**	1.44	1.62	1.83	1.75	1.26	1.50	1.30
**21**	1.43	1.56	1.25	1.28	1.87	1.84	1.60

Supplementary Table 1 | Comparison of the ^{1}H NMR chemical shifts (δ given in ppm) of the different diastereomeric forms of motor 1 found experimentally (–90 °C, CD_2Cl_2, 400 MHz) and calculated at the MPW1K level of theory using the 6-31+G(d,p) basis set. Chemical shifts (δ) of methyl groups are averaged over the values of all three individual protons.

CCDC code	*Z*-(*S*)-(*P*)/*Z*-(*R*)-(*M*) 1 CCDC 1061969	*E*-(*S*)-(*P*)/*E*-(*R*)-(*M*) 1 CCDC 1061970	*Z*-(*R*)-(*M*) 1 CCDC 1406625
net formula	$C_{21}H_{20}O_4S$	$C_{21}H_{20}O_4S$	$C_{21}H_{20}O_4S$
M_r/g mol^{-1}	368.43	368.447	368.43
crystal size/mm	0.100 × 0.100 × 0.020	0.120 × 0.100 × 0.080	0.100 ◊ 0.100 ◊ 0.090
T/K	100(2)	100(2)	173(2)
radiation	MoKα	'Mo Kα	MoKα
diffractometer	'Bruker D8Venture'	'Bruker D8Venture'	'Bruker D8Quest'
crystal system	monoclinic	triclinic	tetragonal
space group	'P 21/n'	*P*1bar	'P 43 21 2'
a/Å	10.6790(5)	8.4090(6)	9.0715(3)
b/Å	7.4911(4)	10.5126(8)	9.0715(3)
c/Å	22.9099(12)	10.9239(8)	44.2312(13)
α/°	90	78.6505(19)	90
β/°	101.3885(15)	85.517(2)	90
γ/°	90	69.269(2)	90
V/Å^3	1796.65(16)	885.45(11)	3639.9(3)
Z	4	2	8
calc. density/g cm^{-3}	1.362	1.38196(17)	1.345
μ/mm^{-1}	0.204	0.207	0.201
absorption correction	multi-scan	multi-scan	multi-scan
transmission factor range	0.8985–0.9580	0.9167–0.9585	0.9402–0.9705
refls. measured	28133	20364	39576
R_{int}	0.0592	0.0721	0.0344
mean σ(I)/I	0.0379	0.0598	0.0173
θ range	3.269–25.35	3.07–26.42	2.292–26.43
observed refls.	2513	2546	3519
x, y (weighting scheme)	0.0380, 0.7992	0.0416, 1.2356	0.0419, 0.7182
hydrogen refinement	constr	constr	constr
Flack parameter			−0.004(16)
refls in refinement	3284	3632	3725
parameters	239	239	239
restraints	0	0	0
$R(F_{obs})$	0.0346	0.0549	0.0301
$R_w(F^2)$	0.0853	0.1248	0.0781
S	1.060	1.032	1.092
shift/error$_{max}$	0.001	0.001	0.001
max electron density/e Å^{-3}	0.328	1.213	0.160
min electron density/e Å^{-3}	−0.340	−0.415	−0.256

Supplementary Table 2 | Crystal structural data for the *Z*-(*S*)-(*P*)/*Z*-(*R*)-(*M*), *E*-(*S*)-(*P*)/*E*-(*R*)-(*M*), and *Z*-(*R*)-(*M*) isomers of motor 1.

CCDC code	***Z*-(*P*)/*Z*-(*M*) / *E*-(*P*)/*E*-(*M*) deoxy-1 CCDC 1408096**	***E*-(*P*)/*E*-(*M*) deoxy-1 CCDC 1408097**
net formula	$C_{21}H_{20}O_3S$	$C_{21}H_{20}O_3S$
M_r/g mol^{-1}	352.43	352.448
crystal size/mm	0.100 × 0.080 × 0.060	0.151 × 0.118 × 0.074
T/K	173(2)	100(2)
radiation	MoKα	'Mo Kα
diffractometer	'Bruker D8Quest'	'Bruker D8Venture'
crystal system	monoclinic	orthorhombic
space group	'P 21/c'	*Pbca*
a/Å	10.3725(4)	8.3184(9)
b/Å	9.9208(4)	14.5702(19)
c/Å	17.5116(6)	28.735(4)
α/°	90	90
β/°	96.9373(12)	90
γ/°	90	90
V/Å^3	1788.81(12)	3482.7(7)
Z	4	8
calc. density/g cm^{-3}	1.309	1.3444(3)
μ/mm^{-1}	0.198	0.203
absorption correction	multi-scan	multi-scan
transmission factor range	0.8825–0.9582	0.9158–0.9580
refls. measured	29357	22007
R_{int}	0.0478	0.0604
mean σ(*I*)/*I*	0.0246	0.0533
θ range	2.343–25.37	2.80–25.44
observed refls.	2846	2283
x, *y* (weighting scheme)	0.0291, 1.3374	0.0547, 7.5564
hydrogen refinement	constr	constr
Flack parameter		
refls in refinement	3274	3192
parameters	258	230
restraints	0	0
$R(F_{obs})$	0.0501	0.0611
$R_w(F^2)$	0.1175	0.1470
S	1.220	1.035
shift/error$_{max}$	0.001	0.001
max electron density/e Å^{-3}	0.183	1.150
min electron density/e Å^{-3}	−0.233	−0.420

Supplementary Table 3 | Crystal structural data for *Z*-(*P*)/*Z*-(*M*) / *E*-(*P*)/*E*-(*M*) deoxy-1 and *E*-(*P*)/*E*-(*M*) deoxy-1.

**bond lengths** in Å	***Z*-(*S*)-(*P*)/*Z*-(*R*)-(*M*)**		***E*-(*S*)-(*P*)**	
	exp.	calc.	exp.	calc.
S-O	1.494/1.490	1.495	1.487	1.498
C(8)-C(9)	1.358/1.356	1.353	1.351	1.350
C(1)-O(carbonyl)	1.224/1.210	1.212	1.220	1.208

**bond angles**	***Z*-(*S*)-(*P*)/*Z*-(*R*)-(*M*)**		***E*-(*S*)-(*P*)**	
	exp.	calc.	exp.	calc.
C(2)-C(1)-C(8)	110.2°/109.8°	109.9°	109.7°	109.5°
C(1)-C(8)-S	109.0°/109.8°	109.4°	110.7°	110.5°
C(7)-S-C(8)	89.0°/89.7°	89.3°	89.9°	89.9°
S-C(8)-C(9)	123.0°/122.0°	123.1°	122.0°	122.1°
C(8)-C(9)-C(10)	128.0°/127.8°	127.6°	126.8°	126.1°
C(10)-C(9)-C(19)	106.6°/106.4°	106.5°	105.6°	106.2°
C(9)-C(19)-C(18)	102.5°/103.5°	102.5°	101.4°	101.2°
C(19)-C(18)-C(17)	104.6°/105.0°	104.6°	103.9°	103.8°

**dihedral angles**	***Z*-(*S*)-(*P*)/*Z*-(*R*)-(*M*)**		***E*-(*S*)-(*P*)**	
	exp.	calc.	exp.	calc.
C(7)-C(8)-S-O(S)	108.9°/110.2°	-110.5°	110.5°	-111.2°
C(8)-C(9)-C(10)-C(11)	24.5°/22.7°	25.1°	33.3°	30.8°
S-C(8)-C(9)-C(10)	12.4°/14.3°	14.7°	171.7°	-167.8°
C(2)-C(1)-C(8)-O(carbonyl)	176.1°/178.1°	177.1°	174.2°	175.2°
O(carbonyl)-C(1)-C(8)-C(9)	30.8°/23.0°	27.4°	11.2°	17.1°
C(9)-C(19)-C(18)-H18	96.8°/99.5°	-95.4°	-90.2°	-90.5°
C(9)-C(19)-C(18)-H18'	142.1°/139.7°	145.4°	148.4°	150.0°
C(20)-C(19)-C(18)-H18	148.7°/145.1°	149.4°	155.7°	154.4°
C(21)-C(19)-C(18)-H18	27.4°/23.2°	28.6°	35.4°	33.6°
C(20)-C(19)-C(18)-H18'	27.7°/24.4°	30.2°	34.3°	35.0°
C(21)-C(19)-C(18)-H18'	93.6°/97.6°	-90.6°	-86.0°	-85.9°

**intramolecular distances** in Å	***Z*-(*S*)-(*P*)/*Z*-(*R*)-(*M*)**		***E*-(*S*)-(*P*)**	
	exp.	calc.	exp.	calc.
C(21)-H(3)	4.912/5.107	4.915	-	-
C(12)-H(6)	5.571/5.491	5.664	-	-
C(21)-H(6)	-	-	5.807	5.892
C(12)-H(3)	-	-	5.071	4.569

Supplementary Table 4 | Structural comparison of calculated (DFT MPW1K level of theory using the 6-31+G(d,p) basis set) and experimentally (crystal structure analysis) determined geometries of the *Z*-(*S*)-(*P*) (CCDC 1061969), *Z*-(*R*)-(*M*) (CCDC 1406625), and *E*-(*S*)-(*P*) (CCDC 1061970) isomers of motor 1.

Supplementary Methods

Thermodynamic and kinetic analysis of the thermal *E*-(*S*)-(*P*)/*E*-(*R*)-(*M*) to *Z*-(*S*)-(*P*)/*Z*-(*R*)-(*M*) isomerization

The thermal *E*-(*S*)-(*P*)/*E*-(*R*)-(*M*) to *Z*-(*S*)-(*P*)/*Z*-(*R*)-(*M*) isomerization of motor **1** was followed by ^{1}H NMR spectroscopy and could be analyzed using a first-order kinetics description (Supplementary Fig. 21). The first-order rate constant for the thermal *E*-(*S*)-(*P*)/*E*-(*R*)-(*M*) to *Z*-(*S*)-(*P*)/*Z*-(*R*)-(*M*) isomerization is $k_{(\text{therm.}E\rightarrow Z)}$ = 0.000038 s^{-1} at 100 °C (Supplementary Fig. 21c). The activation energy ΔG^* for the thermal *E*/*Z* isomerization can be calculated from the rate constant $k_{(\text{therm.}E\rightarrow Z)}$ of the reaction using the Eyring equation:

$$k_{(\text{therm.}E\rightarrow Z)} = \frac{k_B\ T}{h}\ \mathrm{e}^{\frac{-\Delta G*}{RT}} \qquad \text{(Equation 1)}$$

with k_B = Boltzmann constant ($1.381\cdot10^{-23}$ J·K^{-1})
T = temperature in K
h = Planck constant ($6.626\cdot10^{-34}$ J·s)
$k_{(\text{therm.}E\rightarrow Z)}$ = rate constant of the thermal *E*-(*S*)-(*P*)/*E*-(*R*)-(*M*) to *Z*-(*S*)-(*P*)/*Z*-(*R*)-(*M*) isomerization

After rearranging Supplementary Equation 1 and insertion of the numerical values of the constants $\Delta G*$ is given by:

$$\Delta G^* \ (\text{in J mol}^{-1}) = 8.314\ \cdot T\ \cdot \left[23.760 + \ln\left(\frac{T}{k(\text{therm.}E\rightarrow Z)}\right)\right] \qquad \text{(Equation 2)}$$

In this way a ΔG^* = 123.7 kJ·mol^{-1} = 29.5 kcal·mol^{-1} was obtained for the thermal *E*-(*S*)-(*P*)/*E*-(*R*)-(*M*) to *Z*-(*S*)-(*P*)/*Z*-(*R*)-(*M*) isomerization of motor **1**.

The stationary isomer composition of motor **1** at 100 °C is 75% *Z*-(*S*)-(*P*)/*Z*-(*R*)-(*M*) isomers and 25% *E*-(*S*)-(*P*)/*E*-(*R*)-(*M*) isomers. From this equilibrium ratio the relative energy difference between the two isomeric states $-\Delta G$ can be calculated using:

$$-\Delta G = R\cdot T\cdot \ln K \qquad \text{(Equation 3)}$$

with K = equilibrium constant = [*Z*-(*S*)-(*P*)/*Z*-(*R*)-(*M*)]/[*E*-(*S*)-(*P*)/*E*-(*R*)-(*M*)]
R = ideal gas constant = (8.314 J·K^{-1}·mol^{-1})
T = temperature in K

Using K = 75/25 for the equilibrium constant at 100 °C gives an energy difference between the *Z*-(*S*)-(*P*)/*Z*-(*R*)-(*M*) and *E*-(*S*)-(*P*)/*E*-(*R*)-(*M*) isomers of 0.82 kcal·mol^{-1}.

To confirm the energy difference measured at 100 °C the equilibrium distribution of isomers was measured again at 130 °C in *o*-xylene-d_{10} as solvent as shown in Supplementary Fig. 22. The stationary isomer composition of motor **1** at 130 °C is 73% *Z*-(*S*)-(*P*)/*Z*-(*R*)-(*M*) isomers and 27% *E*-(*S*)-(*P*)/*E*-(*R*)-(*M*) isomers. From this equilibrium ratio the relative energy difference between the two isomeric states was calculated to be $-\Delta G$ = 0.81 kcal·mol^{-1}, consistent with the value obtained from the 100 °C measurement. The experiment at 130 °C was repeated 4 times at a concentration of 2.5 mM, two different sets of signals were integrated, and the average value was taken for the quantification of the equilibrium between *Z*-(*S*)-(*P*)/*Z*-(*R*)-(*M*) and *E*-(*S*)-(*P*)/*E*-(*R*)-(*M*) isomers at 130 °C. Possible temperature deviations of ±5 °C during the experiments only lead to minor errors compared to the integration errors. The largest errors found are in the range of ±0.07 kcal/mol for $-\Delta G$.

Thermodynamic and kinetic analysis of the thermal *E*-(*S*)-(*M*)/*E*-(*R*)-(*P*) to *E*-(*S*)-(*P*)/*E*-(*R*)-(*M*) isomerization

The thermal *E*-(*S*)-(*M*)/*E*-(*R*)-(*P*) to *E*-(*S*)-(*P*)/*E*-(*R*)-(*M*) isomerization of motor **1** was followed by ^{1}H NMR spectroscopy and could be analyzed using a first-order kinetics description (Supplementary Fig. 30).

The first-order rate constant for the thermal *E*-(*S*)-(*M*)/*E*-(*R*)-(*P*) to *E*-(*S*)-(*P*)/*E*-(*R*)-(*M*) isomerization is $k_{(\text{therm.}ESM\rightarrow ESP)}$ = 0.00078 s^{-1} at –90 °C (Supplementary Fig. 30c). The activation energy ΔG^* for this thermal *E*/*E* isomerization can be calculated from the rate constant $k_{(\text{therm. }ESM\rightarrow ESP)}$ of the reaction using the Eyring equation:

$$k_{(\text{therm. }ESM\rightarrow ESP)} = \frac{k_B\ T}{h}\ \mathrm{e}^{\frac{-\Delta G*}{RT}} \qquad \text{(Equation 4)}$$

with k_B = Boltzmann constant ($1.381\cdot10^{-23}$ J·K^{-1})
T = temperature in K
h = Planck constant ($6.626\cdot10^{-34}$ J·s)
$k_{(\text{therm. }ESM\rightarrow ESP)}$ = rate constant of the thermal *E*-(*S*)-(*M*)/*E*-(*R*)-(*P*) to *E*-(*S*)-(*P*)/*E*-(*R*)-(*M*) isomerization

After rearranging Supplementary Equation 4 and insertion of the numerical values of the constants ΔG^* is given by:

$$\Delta G^*\ (\text{in J mol}^{-1}) =\ 8.314\ \cdot T\ \cdot\ \left[23.760 + \ln\left(\frac{T}{k(\text{therm.}ESM\rightarrow ESP)}\right)\right] \qquad \text{(Equation 5)}$$

In this way a ΔG^* = 54.8 kJ·mol^{-1} = 13.1 kcal·mol^{-1} was obtained for the thermal *E*-(*S*)-(*M*)/*E*-(*R*)-(*P*) to *E*-(*S*)-(*P*)/*E*-(*R*)-(*M*) isomerization of motor **1**.

After about 60 min in the dark almost no residual signals of the *E*-(*S*)-(*M*)/*E*-(*R*)-(*P*) isomers are seen. An additional annealing experiment (Supplementary Fig. 31) confirmed that the *E*-(*S*)-(*M*)/*E*-(*R*)-(*P*) isomers are completely (>95% given the accuracy of the NMR experiment) converted to the *E*-(*S*)-(*P*)/*E*-(*R*)-(*M*) isomers in the thermal step. The completeness of the thermal step determines the degree of unidirectionality during motor operation. Therefore, the photoinduced *Z*-(*S*)-(*P*)/*Z*-(*R*)-(*M*) to *E*-(*S*)-(*P*)/*E*-(*R*)-(*M*) isomerization of motor **1** is >95% unidirectional at –90 °C.

We also performed shock-freezing experiments to determine the thermal equilibrium between the *E*-(*S*)-(*M*)/*E*-(*R*)-(*P*) and *E*-(*S*)-(*P*)/*E*-(*R*)-(*M*) isomers at 22 °C and at 100 °C. A sample of motor **1** in CD_2Cl_2 at 22 °C was shock frozen in liquid N_2, inserted into the NMR spectrometer precooled to –90 °C, and ^{1}H NMR spectra were recorded in 2 min intervals during and after thawing of the frozen sample (see Supplementary Fig. 32). The first spectrum could be acquired after 165 s, which is considerably shorter than the half life of thermal *E*-(*S*)-(*M*)/*E*-(*R*)-(*P*) to *E*-(*S*)-(*P*)/*E*-(*R*)-(*M*) isomerization (i.e. 9.30 min) at that temperature. No signals of the *E*-(*S*)-(*M*)/*E*-(*R*)-(*P*) isomers could be observed showing that also at 22 °C unidirectionality is >95 %. A similar experiment was conducted in toluene-d_8 starting from 100 °C. Again, no signals of the *E*-(*S*)-(*M*)/*E*-(*R*)-(*P*) isomers could be obtained in this experiment, which shows that also at 100 °C unidirectionality is >95 %. This gives an energy difference between the *E*-(*S*)-(*M*)/*E*-(*R*)-(*P*) and *E*-(*S*)-(*P*)/*E*-(*R*)-(*M*) isomers of at least 2.18 kcal/mol according to the relation of the change of *Gibbs* free energy and the equilibrium constant $-\Delta G = R \cdot T \cdot \ln K$ - in good agreement with the theoretical description.

Irradiation experiments

Upon irradiation (365 nm - 505 nm) of motor **1** at 23 °C only interconversion between the *Z*-(*S*)-(*P*)/*Z*-(*R*)-(*M*) isomers and the *E*-(*S*)-(*P*)/*E*-(*R*)-(*M*) isomers is observed. The quantum yields for photoisomerization at ambient temperature are 30% for the *Z* to *E* isomerization and 12% for the *E* to *Z* isomerization.
Cooling a solution of the *Z*-(*S*)-(*P*)/*Z*-(*R*)-(*M*) isomers in CD_2Cl_2 to –90 °C without irradiation does not lead to significant changes of the signals chemical shifts (Supplementary Fig. 26). Upon irradiation of a solution of the *Z*-(*S*)-(*P*)/*Z*-(*R*)-(*M*) isomers in CD_2Cl_2 at –90 °C with high power 460 nm light a new set of signals is observed, which thermally convert into the signals of the *E*-(*S*)-(*P*)/*E*-(*R*)-(*M*) isomers (Supplementary Fig. 27 and Supplementary Fig. 28). Based on the chemical shifts (Supplementary Table 1), thermal behavior, and theoretical simulation (Supplementary Fig. 29), the new set of signals is assigned to the *E*-(*S*)-(*M*)/*E*-(*R*)-(*P*) isomers.
When using medium power 460 nm light for the irradiation of pure *Z*-(*S*)-(*P*)/*Z*-(*R*)-(*M*) isomers the signals of the *E*-(*S*)-(*M*)/*E*-(*R*)-(*P*) isomers are observed to build up first and reach their stationary point earlier than the signals of the *E*-(*S*)-(*P*)/*E*-(*R*)-(*M*) isomers, which build up later and also reach their stationary intensity at a later time (Supplementary Fig. 35a).

Upon irradiation of a solution in CD_2Cl_2 at –90 °C with low power 460 nm light no new set of signals is observed but the signals of the *Z*-(*S*)-(*P*)/*Z*-(*R*)-(*M*) isomers are building up directly (Supplementary Fig. 34). Irradiation of the *E*-(*S*)-(*P*)/*E*-(*R*)-(*M*) isomers with medium power

light leads to build-up of first the *Z*-(*S*)-(*P*)/*Z*-(*R*)-(*M*) isomers and only later of the *E*-(*S*)-(*M*)/*E*-(*R*)-(*P*) isomers (Supplementary Fig. 35b).

Therefore, the two isomeric forms *Z*-(*S*)-(*P*)/*Z*-(*R*)-(*M*) and *E*-(*S*)-(*P*)/*E*-(*R*)-(*M*) are interconverted under irradiation conditions via two different routes. The *Z*-(*S*)-(*P*)/*Z*-(*R*)-(*M*) isomers are photochemically converted to the *E*-(*S*)-(*M*)/*E*-(*R*)-(*P*) isomeric forms, which in turn convert thermally to the *E*-(*S*)-(*P*)/*E*-(*R*)-(*M*) isomers completely. At this point microscopic reversibility is broken. The *E*-(*S*)-(*P*)/*E*-(*R*)-(*M*) isomers are not back-converted to the *E*-(*S*)-(*M*)/*E*-(*R*)-(*P*) isomeric forms under irradiation conditions, instead the *Z*-(*S*)-(*P*)/*Z*-(*R*)-(*M*) isomeric state is always observed first before the *E*-(*S*)-(*M*)/*E*-(*R*)-(*P*) isomers are formed again (as a result of irradiation of the newly formed *Z*-(*S*)-(*P*)/*Z*-(*R*)-(*M*) isomers). The two different routes for the photoinduced *Z*/*E* and *E*/*Z* isomerization of motor **1**, respectively, are only consistent with a full unidirectional 360 ° rotation.

Theoretical description

Based on the crystal structure data of the *Z*-(*S*)-(*P*) and *E*-(*S*)-(*P*) enantiomers a relaxed optimization at the MPW1K level of theory with the 6-31+G(d,p) basis set has been done using the Gaussian09 Revision A.02 program package.[1] The following frequency analysis confirmed the structures as a minimum structure since no imaginary frequencies have been found. The calculated minimum structure of the *Z*-(*S*)-(*P*) isomer was used as starting point to obtain the minimum structures of the other diastereomers, which have been obtained by variation of the necessary structural parameters. In particular the dihedrals of the double bond S-C8-C9-C10 (for *E*/*Z* enantiomers) and the single bond C8-C9-C10-C11 (for (*P*)/(*M*) enantiomers) were taken into account. All structures were also confirmed as minimum structures by frequency analysis. The corresponding DFT energies of the minima structures are:

Z-(*S*)-(*P*)-**1**: DFT energy = –1511.02574853 Hartree.
E-(*S*)-(*M*)-**1**: DFT energy = –1511.01844931 Hartree.
E-(*S*)-(*P*)-**1**: DFT energy = –1511.02406327 Hartree.
Z-(*S*)-(*M*)-**1**: DFT energy = –1511.02036729 Hartree.

The same dihedral angles were also varied in a relaxed potential surface scan calculation for both transition state structures. In the obtained maximum structures the motions of all methyl groups in the minimum structures were frozen and a restricted transition state optimization was conducted. In the subsequent transition state optimization the dihedrals were unfrozen, which led to the final structures of the transition states confirmed by frequency analysis. Both transition state structures were shown to be first order saddle points on the potential surface since only one imaginary vibrational mode has been found.

For the NMR shielding calculation the same method and basis set have been used to retain consistency. The TMS reference was also optimized and confirmed as minimum structure by frequency analysis with the same method and basis set. The shifts of the hydrogen atoms of all minimum structures were obtained by calculating the difference of each hydrogen isotropic value of the minimum structures and the calculated TMS reference.

For the calculations of UV/Vis and ECD spectra a greater basis set (6-311++G (d,p)) was used. With the TD-DFT Method 20 states were calculated for each isomer. UV/Vis and ECD calculations have additionally been carried using with a solvent model to better match experimentally determined spectra, which were measured in solution. For this purpose the Polarizable Continuum Model (PCM) has been employed to mimic the CH_2Cl_2 solvent.

Supplementary References

1 Gaussian 09, Revision A.02 (Gaussian, Inc., Wallingford CT, 2009).
